# From Quantum Curves to Topological String Partition Functions II

**DOI:** 10.1007/s00023-025-01538-2

**Published:** 2025-01-29

**Authors:** Ioana Coman, Pietro Longhi, Jörg Teschner

**Affiliations:** 1 Institute of Physics, University of Amsterdam, 1098 XH Amsterdam, The Netherlands; 2 Institut für Theoretische Physik, ETH Zürich, Wolfgang-Pauli-Str. 27, 8093 Zürich, Switzerland; 3 Department of Mathematics, University of Hamburg, Bundesstrasse 55, 20146 Hamburg, Germany; 4 DESY Theory, Notkestrasse 85, 20607 Hamburg, Germany

## Abstract

We propose a geometric characterisation of the topological string partition functions associated with the local Calabi–Yau (CY) manifolds used in the geometric engineering of $d=4$, ${\mathcal {N}}=2$ supersymmetric field theories of class ${\mathcal {S}}$. A quantisation of these CY manifolds defines differential operators called quantum curves. The partition functions are extracted from the isomonodromic tau-functions associated with the quantum curves by expansions of generalised theta series type. It turns out that the partition functions are in one-to-one correspondence with preferred coordinates on the moduli spaces of quantum curves defined using the Exact WKB method. The coordinates defined in this way jump across certain loci in the moduli space. The changes of normalization of the tau-functions associated with these jumps define a natural line bundle playing a key role in the geometric characterisation of the topological string partition functions proposed here.

## Introduction

The goal of this paper is to propose a non-perturbative geometric definition of the topological string partition functions, at least for an interesting family of examples. The examples considered in our paper are Calabi–Yau (CY) manifolds ${{\mathcal {Y}}}_\Sigma$ defined by equations of the form $uv+f_\Sigma  (x,y)=0$, with $f_\Sigma  (x,y)\equiv y^2+q(x)=0$ being the equation defining a curve $\Sigma$ in the cotangent bundle of a Riemann surface *C* equipped with a quadratic differential $q=q(x)(dx)^2$. This class of local CY appears in the geometric engineering of $d=4$, ${\mathcal {N}}=2$ supersymmetric field theories of class ${{\mathcal {S}}}$ from string theory. We will in this paper define a global geometric object, a holomorphic line bundle on a certain moduli space to be specified in detail below, having a canonical section. The functions locally representing the canonical section admit series expansions whose coefficients are in some cases shown and in general conjectured to coincide with previously proposed definitions of topological string partition function.

### Previous Approaches to Topological String Partition Functions

We shall begin by briefly reviewing previous approaches to topological string partition functions. As the subject has grown very large, we will refrain from offering a guide to the literature, and discuss the most prominent directions of research in this field only very briefly.

#### Formal Series Definitions

The worldsheet-approach to string theory suggests to define the topological string partition functions as a formal series in powers of the topological string coupling $\hbar$. Expanding the coefficients of this series as a formal series in exponentials of the complexified Kähler parameters yields a formal series admitting a rigorous definition as generating function of the Gromov–Witten (GW) invariants, see e.g. [73] for a review.

The holomorphic anomaly equations [16] yield relations among the coefficients of this formal series. By supplying boundary conditions one may solve these equations recursively, but the explicit form of the solutions is unknown, in general. Mirror symmetry suggests an interpretation of the holomorphic anomaly equations in the context of the quantisation of the theory of deformations of complex structures of the mirror CY (Kodaira–Spencer theory) [16, 115].

There exist identities of formal power series relating the generating functions of GW invariants to other formal power series in exponential or trigonometric functions of $\hbar$. The coefficients of the resulting formal series are expected, and in some cases rigorously known to admit an interpretation as other types of geometric invariants known as Gopakumar–Vafa [68], or Donaldson–Thomas (DT) invariants [98, 99, 106]. There exist elaborate mathematical tools for the computation of the DT invariants known as wall-crossing formulae [89]. Combined with algebro-geometric or other input it is often possible to compute topological string partition functions with the help of the above-mentioned relations.

The resulting series sometimes admit various (partial) summations, depending on the class of Calabi–Yau manifolds. For toric CY, one can use the *topological vertex* [4] as a tool to derive power series representations having elementary functions formed out of $\hbar$ and the Kähler parameters as coefficients. These series are known to be convergent in some cases, but not in general. The *topological recursion* [47] yields a definition of the coefficients in the expansion of these topological string partition functions as a power series in $\hbar$ [21, 48].

#### Non-perturbative Approaches

All these approaches yield formal series in $\hbar$, or in functions of $\hbar$. Finding actual functions of $\hbar$ having the above-mentioned formal series as asymptotic expansions is expected to be very rewarding. This should offer profound insights into the non-perturbative effects of string theory on the side of physics, and it could reveal deep relations between different types of geometric invariants on the side of mathematics. Such hopes have motivated a long series of efforts aiming at non-perturbative definitions of topological string partition functions.

A first class of candidates are the matrix models, see [93] for a review. One considers certain families of integrals having *N* integration variables, regarding *N* as a variable. These families of integrals may admit asymptotic expansions in 1/*N* having the same form as the topological string partition functions. One may therefore interpret these families of integrals, regarded as functions of *N*, as non-perturbative definitions of the topological string partition functions restricted to lattices in the space of Kähler parameters with lattice spacing $\hbar$.

A more recent research program, often referred to as the TS/ST (topological string/spectral theory) correspondence, proposes spectral determinants of certain finite difference operators as non-perturbative topological string partition functions for toric CY, see [95] for a review. The finite difference operators are obtained by quantisation of the equations for the algebraic curves characterising the mirrors of the toric CY. The spectral determinants forming the basis of this approach are entire functions of the complex structure moduli of the mirror CY.

There exists a lot of supporting evidence for both matrix model approach and the TS/ST correspondence. Rigorous proofs do not seem to be available yet, in general.

Yet another approach starts with the theory of DT-invariants which can be used as basic data defining certain complex-symplectic manifolds from the solutions to problems of Riemann–Hilbert type [24, 25]. The geometric structures of these manifolds can be encoded in certain generating functions. Explicit computations in simple examples indicate that these generating functions can serve as non-perturbative topological string partition functions [24, 26].

#### Relations to Integrable Hierarchies and Free Fermions

Local CY manifolds like ${{\mathcal {Y}}}_{\Sigma }$ are determined by two-dimensional surfaces $\Sigma$. The deformations of the complex structures of such manifolds form the basis of the approach taken in [2], suggesting relations to integrable hierarchies, and to the theory of free fermions on $\Sigma$.

A variant of these relations of particular relevance both for the previous paper [36] and for our work has been proposed in [42]. It considers the dual partition functions

1.1 $$\begin{aligned} Z_{\textrm{D}}(\xi ,a;\hbar )&=\sum _{p\in H^2(Y,{{\mathbb {Z}}})}e^{p\xi }Z_{\textrm{top}}(a+p\hbar ,\hbar ), \end{aligned}$$

where $Z_{\textrm{top}}(a,\hbar )$ is the topological string partition function associated with ${{\mathcal {Y}}}_\Sigma$, the variables *a* being parameters determining the surface $\Sigma$. String-theoretical duality conjectures predict that $Z_{\textrm{D}}(\xi ,a;\hbar )$ can be identified with the partition function of a system of two-dimensional chiral free fermions on a non-commutative deformation of the curve $\Sigma$ [42]. It has furthermore been proposed in [42, 43] that the deformation of $\Sigma$ can be described by an object called quantum curve, in our case represented by a differential operator of the form $\hbar ^2\partial _x^2-q(x)$, obtained from the curve $\Sigma$ by replacing *y* by $-\textrm{i}\hbar \partial _x$.

### Summary of Our Approach

Motivation for the proposal made in the companion paper [36] came from the relations to chiral free fermions proposed in [42], as mentioned above. In order to define topological string partition functions from the quantum curve, it turns out to be useful to consider the dual partition function $Z_{\textrm{D}}(\xi ,a;\hbar )$, for the following reason. Although the topological string partition function can be defined at large volume by existing techniques, naive analytic continuation to other regions of moduli space would yield incorrect results. It turns out that the object having fairly simple analytic properties on moduli space is not the topological string partition function itself, but rather the dual partition function defined in (1.1). Different local definitions of the dual partition functions are related by multiplication with holomorphic transition functions. The study of these issues was initiated in [36] and completed in the present paper.

It was proposed in [36] to replace the quadratic differential *q* defining the quantum curve by a natural $\xi$-dependent deformation $q_{\xi ,\hbar }\equiv q_{\xi ,\hbar }(x)(dx)^2$ such that $q_{\xi ,\hbar }-q={{\mathcal {O}}}(\hbar )$. Using the formalism of free fermion conformal field theory (CFT) one can define partition functions $Z_{\textrm{D}}$ naturally associated with the quantum curves $q_{\xi ,\hbar }$. The conformal Ward identities imply that the partition functions $Z_{\textrm{D}}(\xi ,a;\hbar )$ coincide with well-known objects called the isomonodromic tau-functions if one identifies the variables $\xi$ and *a* with coordinates on the moduli spaces ${{\mathcal {M}}}_{{\textrm{ch}}}(C)$ of flat *SL*(2)-connections on the underlying Riemann surface *C*. The isomonodromic tau-functions are important ingredients of the theory of monodromy preserving deformations of ordinary differential equations [86]. Such deformations can often be represented as Hamiltonian flows with respect to a canonical Poisson structure. The tau-functions then serve as a generating function for the Hamiltonian functions. However, it turns out that this formalism determines the dependence of the partition functions $Z_{\textrm{D}}$ on the variables *a* and $\xi$ only incompletely.

The previous paper [36] made a first step to fix the remaining freedom. A key observation made in [36] was a direct correspondence between certain special choices of the coordinates $\xi$ on the one hand, and fully normalised partition functions $Z_{\textrm{D}}(\xi ,a;\hbar )$ admitting series expansions of the form (1.1), on the other hand. This offers a way to fix the freedom remaining after having identified $Z_{\textrm{D}}(\xi ,a;\hbar )$ as an isomonodromic tau-function by identifying the proper choices of coordinates *a* and $\xi$. In [36], it was furthermore observed in the some examples associated with surfaces *C* of genus zero that the choice of coordinates $(a,\xi )$ must depend on the choice of a chamber in the space of quadratic differentials, in general. A natural procedure for fixing this dependence can be based on the formalism called abelianisation in [74]. The functions $Z_{\textrm{top}}(a,\hbar )$ defined using (1.1) from the partition functions $Z_{\textrm{D}}(\xi ,a;\hbar )$ fully defined in this way were shown in some examples to coincide with the topological string partition functions computed using the topological vertex, chamber by chamber [36].

This paper completes the approach proposed in [36]. We use the fact that the so-called exact WKB method allows us to define an distinguished atlas of systems of coordinates $(a,\xi )$ on the moduli spaces of flat connections on Riemann surfaces. Our main result is that to each coordinate system defined in this way there exist fully normalised partition functions $Z_{\textrm{D}}(\xi ,a;\hbar )$. Two related types of coordinates are relevant, called coordinates of Fenchel–Nielsen (FN) and Fock–Goncharov (FG) types, respectively.

To this aim, we observe that the relations between any two dual partition functions $Z_{\textrm{D}}(\xi ,a;\hbar )$ and $Z_{\textrm{D}}'(\xi ',a';\hbar )$ associated with different choices of coordinates $(a,\xi )$ and $(a',\xi ')$, respectively, are very special, represented by the *difference generating functions* of the changes of coordinates from $(a,\xi )$ and $(a',\xi ')$. Difference generating functions are analogs of the usual generating functions from Poisson geometry defined by relations involving finite difference operators instead of derivatives. The fact that $Z_{\textrm{D}}(\xi ,a;\hbar )$ is related to $Z_{\textrm{D}}'(\xi ',a';\hbar )$ by multiplication with a difference generating function ensures the both $Z_{\textrm{D}}(\xi ,a;\hbar )$ and $Z_{\textrm{D}}'(\xi ',a';\hbar )$ can admit series expansions of the form (1.1). We are going to outline a proof for the existence of difference generating functions for any pair of coordinates $(a,\xi )$ and $(a',\xi ')$ on the moduli spaces ${{\mathcal {M}}}_{{\textrm{ch}}}(C)$ of flat connections on Riemann surfaces defined by the exact WKB method.

The collection of such difference generating functions defines natural line bundles on the moduli spaces ${{\mathcal {M}}}_{{\textrm{ch}}}(C)$, with $Z_{\textrm{D}}$ being local holomorphic sections of this line bundle. This geometric object is proposed to define the topological string partition functions by means of the expansions (1.1). This proposal is supported by detailed checks performed in [36] and here.

### Relations to the Existing Literature

When we submitted the first version of this paper to the arXiv, the relations between previous approaches to the definition of non-perturbative partition functions and our approach have been mostly unclear to us. By combining previously known results with some more recent developments, we may now offer a brief discussion of some of the relations. As the relations to the isomonodromic tau-functions play an important role for us, we will begin with a brief discussion of the relations between the literature on isomonodromic tau-functions and our results.

#### Relations to the Literature on Isomonodromic Tau-Functions

It has been a long-standing problem to fix the dependence of the isomonodromic tau-functions on the monodromy data in a natural way. A set of equations fixing the monodromy dependence of the tau-functions has been proposed in [79]. The geometric meaning of these equations has been clarified in the work of Bertola-Korotkin, identifying the tau-functions as generating functions of the monodromy symplectomorphisms [31].

A central ingredient of our approach is another, a priori different, definition of fully normalised isomonodromic tau-functions. Our approach in [36] and the present paper is based on the discovery of [57] that the tau-functions $Z_{\textrm{D}}(\xi ,a;\hbar )$ can be represented in the form (1.1), with $Z_{\textrm{top}}(a,\hbar )$ identified with the conformal blocks of the Virasoro algebra at $c=1$. The conjecture of [57] has been proven in [30, 60, 80, 102] by remarkably different methods.

One of the main points here is to introduce a global object on the space of monodromy data ${{\mathcal {M}}}$ which is locally represented by the partition functions $Z_{\textrm{D}}(\xi ,a;\hbar )$ admitting expansion of the form (1.1) associated with local systems of coordinates $(\xi ,a)$ defined on certain chambers in ${{\mathcal {M}}}$ forming a cover of this space. It is crucial to ensure that changes of coordinates from chamber to chamber preserve the existence of expansions of the form (1.1). We will explain in our paper how this principle can be used to fix the dependence of the tau-functions $Z_{\textrm{D}}(\xi ,a;\hbar )$ on the monodromy data.

Both [31, 79] and the approach taken in this paper define natural line bundles with a holomorphic connection on the space of monodromy data. However, the relation between these two line bundles was not clear to us at the time of the submission of the first version of our paper. The transition functions characterising the line bundle in the approach of [31, 79] are generating functions of the symplectomorphisms relating different sets of Darboux coordinates on ${{\mathcal {M}}}$. Our approach characterises the transition function as *difference* generating functions of the same symplectomorphisms, functions characterised as solutions to finite difference equations rather than the differential equations determining ordinary generating functions up to constants.

Remarkably, it turns out that the results of these two, a priori different looking approaches are essentially equivalent. One may note that the precise relation of the approach of [31, 79] to the approach proposed in our paper, based on the series expansions (1.1), follows for the typical case of $C=C_{0,4}$ from the results of [38, 102]. These results establish the precise relation between an isomonodromic tau-function admitting expansions of the form (1.1) and the generating function of the monodromy symplectomorphism. One may furthermore check by explicit computation for elementary coordinate transformations that ordinary and difference generating functions of the same symplectomorphism are closely related, as is necessary for the mutual consistency of the results mentioned above. Within the framework described in this paper one can use these results in order to establish the equivalence of the approaches [31, 79] to the one used here. These relations should be discussed in more detail.^1^

#### Comparison with Bridgeland’s Program and the TS/ST-Correspondence

At the time of submission of the first version of this paper, it was unclear to us how the approach of T. Bridgeland is related to our approach. More recent developments indicate that the approach of Bridgeland, specialised to the spaces of stability conditions on spaces of quadratic differentials defined in [23], ultimately leads to the same candidates for non-perturbative topological string partition functions as proposed here. The complex-symplectic structures from the theory of DT-invariants admit natural generating functions called $\tau$-functions in [29]. It was furthermore shown in [29] that specialising these generating functions to the spaces of quadratic differentials defined in [23] yields the isomonodromic tau-functions.

Comparing our approach with the TS/ST-correspondence is still not straightforward. Both approaches use differential operators called quantum curves as key ingredients. They differ substantially in the way how the quantum curves are used to define candidates for non-perturbative partition functions. While the TS/ST-correspondence is based on the spectral problems of the quantum curves, our approach studies isomonodromic deformations of the quantum curves.

There are not very many cases where a direct comparison of the results is possible. A large part of the literature on the TS/ST-correspondence studies the mirrors of toric CY. Some of these local CY admit a limit recovering the local CY ${{\mathcal {Y}}}_{\Sigma }$ studied here if the underlying Riemann surfaces *C* has genus $g=0$ or $g=1$. How to recover the local CY ${{\mathcal {Y}}}_{\Sigma }$ associated with surfaces *C* with $g>1$ in this way does not seem to be well-understood at the moment. The number of cases where a direct comparison with our approach is possible is therefore somewhat limited.

It has been demonstrated in [20] that the tau-function of the Painlevé III equation is related to a limit of the spectral determinants appearing in the TS/ST correspondence by specialising a part of the variables the tau-function depends on. Variants of the TS/ST-correspondence for local CY ${{\mathcal {Y}}}_{\Sigma }$ associated with some surfaces of genus $g=0$ and $g=1$ have been studied in [19, 55], exhibiting relations between the spectrum of differential operators of Mathieu and Calogero-Moser-type and isomonodromic tau-functions.

These results indicate that the TS/ST-correspondence is deeply related to the approach proposed in our paper. However, whenever one can compare results concretely, there seem to exist different types of representations of the partition functions as Fredholm determinants, directly related to the isomonodromic tau-functions in some cases, less directly in others. An important conceptual difference is the feature that the spectral determinants considered in the TS/ST correspondence are entire functions on the complex structure moduli spaces of the local CY, whereas our approach considers locally defined partitions functions representing sections of a line bundle on a fibration over the complex structure moduli spaces of such CY.

#### Non-perturbative Effects and Resurgence

The matrix model approach has offered important insights into non-perturbative effects in topological string theory, see [94, 96] for some early work in this direction, relating them to D-brane effects in some examples. In [33] it has been proposed that ideas from the theory of resurgence could allow one to compute such effects more explicitly using trans-series as an ansatz for the solution of the holomorphic anomaly equations.

Ideally, one might hope that a canonical definition of non-perturbative topological string partition functions could be found by applying a summation method like Borel summation to the formal generating series of the GW invariants. One may expect Stokes phenomena relating different locally defined summations. An instructive example illustrating this scenario is the resolved conifold. The Borel summation of the topological string partition function indeed exhibits Stokes phenomena in this case [12, 76, 107]. It has been shown in [12] that the Stokes jumps coincide with the difference generating functions of the corresponding changes of coordinates in the Riemann–Hilbert-type problem defined by Bridgeland [26] in this case.

The trans-series solutions of the holomorphic anomaly equation have recently been used to find exact results for the Stokes jumps in a large class of examples including even topological string theories on compact CY [59, 66]. Based on these results, an explicit general formula for was found in [83] for the Stokes jumps of the dual partition functions.

Concerning the relation between the results of [83] and the approach taken in our paper, one may note that the coordinates $(\xi ,a)$ defined by exact WKB are known as quantum periods in the literature on topological string theory. The Stokes jumps of these coordinates are represented by formulae known from the theory of cluster algebras [5, 39]. These jumps get interpreted as formulae for changes of coordinates in our approach. The consistency of the expansions (1.1) with the Stokes jumps of the quantum periods requires that the Stokes jumps of $Z_{\textrm{D}}(\xi ,a;\hbar )$ must be given by the difference generating functions of the corresponding changes of coordinates. The results of [83] establish the resulting characterisation of the Stokes jumps more directly.

The results of [83] may therefore be taken as support for the conjecture that the geometric characterisation of dual partition functions based on the symplectic geometry of the string theory moduli spaces proposed here can be generalised to much larger classes of CY manifolds.

### Contents

We start by introducing a somewhat more general class of quantum curves compared to [36] in Sect. 2, having irregular singularities of the simplest type. In Sect. 3, we begin by revisiting known results on expansions of the form (1.1) for the examples with regular and irregular singularities from the perspective of [36]. We show that the variables appearing in some of these expansions admit a geometric interpretation as coordinates of Fock–Goncharov (FG) type associated with triangulations of the surface *C* rather than the coordinates of Fenchel–Nielsen (FN) type associated with pants decompositions of *C* encountered in [36].

Section 4 discusses the relations between the expansions of the form (1.1) associated with different types of coordinates in the two basic examples $C=C_{0,2}$ with two irregular singularities, and $C=C_{0,4}$ with four regular singularities. It turns out that the change of coordinates formulae for the case of $C=C_{0,2}$ can serve as a building block for more complicated cases like $C=C_{0,4}$, a result that will be crucial later.

Both types of coordinates can be naturally described using abelianisation. At the beginning of Sect. 5, we review how the relevant coordinates can be characterised in terms of the Exact WKB method using the Borel summation of the WKB expansions of the monodromy data called Voros symbols. We then demonstrate in a basic example that the coordinates of FN type can be understood as limits of coordinates of FG type. Section 6 describes how one can associate normalised tau-functions to the coordinates defined using the Exact WKB method.

The experience gathered from the examples studied up to this point then allows us to outline how the characterisation of the normalised partition functions should work more generally. Sections 7–9 describe the generalisation of our proposal for the cases associated with Riemann surfaces $C_{g,n}$ of arbitrary genus *g* and *n* punctures.

We conclude by presenting a summary and discussing relations to several other directions of research in Sect. 10.

## Quantum Curves

In this section, we will review how to define the deformed quantum curves $\hbar ^2\partial _x^2-q_{\xi ,\hbar }(x)$ playing a basic role in our approach. Apart from clarifying some aspects of the framework presented in [36] that will become relevant later, we will furthermore explain how to generalise this approach to a simple case with irregular singularities by taking a certain collision limit. This case will later exhibit important new features. We will then briefly explain how CFT allows one to define the corresponding partition functions both in the regular and the irregular cases.

### Classical Curves

The classical Seiberg–Witten curve $\Sigma$ for class ${\mathcal {S}}$-theories associated with Riemann surfaces $C_{0,n}={\mathbb {P}}^1\backslash \{z_1,\ldots ,z_n\}$ of genus zero with *n* regular punctures is defined as a double cover of the *n*-punctured sphere defined by the equation

2.1 $$\begin{aligned} \Sigma =\{(x,y)\subset T^*C_{0,n} \, | \, y^2+q(x) = 0\} ~,~\text {where}~~ q(x)=\sum _{r=1}^n\left( \frac{a_r}{(x-z_r)^2}+\frac{E_r}{x-z_r}\right) ~. \end{aligned}$$

The parameters $E_r$ satisfy three linear equations ensuring that the quadratic differential $q(x)d^2x$ is regular at $x\rightarrow \infty$. For the case $n=4$ one thereby finds that only one of these parameters is independent. Taking into account these constraints, the Seiberg–Witten curve $\Sigma$ corresponding to the *SU*(2) SYM theory with four flavours considered in [36] can be represented as

2.2 $$\begin{aligned} q(x) =&\frac{a_1^2}{(x-z_1)^2} + \frac{a_2^2}{(x-z_2)^2} + \frac{a_3^2}{(x-z_3)^2} - \frac{\kappa }{(x-z_1)(x-z_3)} \nonumber \\&+ \frac{(z_2-z_1)(z_2-z_3)}{(x-z_1)(x-z_2)(x-z_3)}H ~, \end{aligned}$$

where $\kappa =a_1^2+a_2^2+a_3^2-a_4^2$, having moved the puncture $z_4$ to infinity for later convenience.

Irregular singularities can then be created by colliding regular singular points. To this aim, it is convenient to set $z_1=-\frac{1}{2}\epsilon \Lambda ^4$, $z_2=\frac{1}{2}\epsilon \Lambda ^4$. Writing the first two terms in (2.2) as

$$\begin{aligned} \frac{a_1^2}{(x+\frac{\epsilon }{2}\Lambda ^4)^2} + \frac{a_2^2}{(x-\frac{\epsilon }{2}\Lambda ^4)^2}&=\frac{\frac{\epsilon ^2}{4}(a_1^2+a_2^2)\Lambda ^8+\epsilon (a_2^2-a_1^2)\Lambda ^4x+(a_1^2+a_2^2)x^2}{(x^2-\frac{\epsilon ^2}{4}\Lambda ^8)^2}, \end{aligned}$$

and considering $\epsilon$-dependent parameters $a_1^2=-\frac{1}{2\epsilon \Lambda ^2}$, $a_2^2=\frac{1}{2\epsilon \Lambda ^2}$ and $H=\frac{1}{\epsilon \Lambda ^4}U$, one finds that the limit $\epsilon \rightarrow 0$ of (2.2) exists and can be represented as

2.3 $$\begin{aligned} q'(x) = \frac{\Lambda ^2}{x^3} + \frac{a_3^2}{(x-z_3)^2} - \frac{\kappa }{x(x-z_3)} - \frac{z_3}{x^2(x-z_3)}U ~. \end{aligned}$$

In a very similar way one may define a limit $z_3\rightarrow \infty$ further simplifying (2.3) to

2.4 $$\begin{aligned} q''(x) =\frac{\Lambda ^2}{x^3} + \frac{U}{x^2} + \frac{\Lambda ^2}{x} ~. \end{aligned}$$

This is known to be the quadratic differential which defines the Seiberg–Witten curve for the pure *SU*(2) theory.

### Quantisation

Having discussed the classical curve, we briefly review its quantisation following [36]. A non-commutative deformation of the algebra of functions on the Seiberg–Witten curve is defined by introducing the commutation relation $[y,x]=-{\textrm{i}}{\hbar }$ represented by $y\rightarrow -\textrm{i}{\hbar }\partial _x$. The equation defining $\Sigma$ becomes replaced by a second-order differential equation

2.5 $$\begin{aligned} {\mathcal {D}}_q\chi (x)=0~,~\text {where}~~ {\mathcal {D}}_q=\hbar ^2\partial _x^2 -q (x)~. \end{aligned}$$

Such differential equations are closely related to the equations defining flat sections of connections of the form $$dx\left( \hbar \partial _x-\left( {\begin{smallmatrix} 0 &  q\\ 1 &  0\end{smallmatrix}}\right) \right) $$ called opers. The holonomies of opers define a half-dimensional subspace in the character variety ${\mathcal {M}}_{\textrm{ch}}(C)=\textrm{Hom}(\pi _1(C),\textrm{SL}(2,{\mathbb {C}}))/\textrm{SL}(2,{\mathbb {C}})$ called the variety of opers.

As noted above, it is useful to consider deformations $q_{\xi ,\hbar }(x)=q(x)+{\mathcal {O}}(\hbar )$ of the function *q*(*x*) in (2.5) introducing additional parameters $\xi$. This can be done by introducing additional poles of a very special form called apparent singularities. A second-order pole of $q_{\xi ,\hbar } (x)$ at $x=u$ is called apparent singularity if $q_{\xi ,\hbar }(x)=\frac{3\hbar ^2}{4(x-u)^2}-\hbar \frac{v}{x-u}+q_{u}+{\mathcal {O}}(x-u)$, with $q_u\equiv q(u)$ and *v* related by $v^2=q_u$. This ensures that the differential equation $(\hbar ^2\partial _x^2 -q_{\xi ,\hbar }(x))\chi (x)=0$ has two linearly independent solutions $\chi _\pm  (x)$ of the form $\chi _\pm  (x)=(x-u)^{\frac{1}{2}\pm 1}(1+{{\mathcal {O}}}(x-u))$, implying that the monodromy around $x=u$ is trivial in $\textrm{PSL}(2,{{\mathbb {C}}})$. Each apparent singularity introduces one new parameter as *u* and *v* are related by $v^2=q_u$. The parameters associated with the apparent singularities will be related to the parameters $\xi$ by the Riemann–Hilbert correspondence discussed later. This being understood we will mostly use the notation $q_\hbar$ for $q_{\xi ,\hbar }$.

In the case $C=C_{0,4}$ with $z_1=0$, $z_2=z$, $z_3=1$ one would thereby be led to consider $\hbar$-corrections of the form^2^

2.6 $$\begin{aligned} \begin{aligned} q_\hbar (x)=&\, \frac{a_1^2}{x^2} + \frac{a_2^2}{(x-z)^2} + \frac{a_3^2}{(x-1)^2} - \frac{a_1^2+a_2^2+a_3^2-a_4^2}{x(x-1)}+ \frac{z(z-1)}{x(x-1)(x-z)}H \\&-\hbar \frac{u(u-1)}{x(x-1)(x-u)}v+\frac{3}{4}\frac{\hbar ^2}{(x-u)^2} ~. \end{aligned} \nonumber \\ \end{aligned}$$

It is elementary to show that any function $q_\hbar (x)$ defining a quadratic differential $q_\hbar \equiv q_\hbar (x)(dx)^2$ with regular singularities at $x=0,z,1,\infty$ and an apparent singularity at $x=u$ can be represented in the form (2.6). The constraint $v^2=q_u$ determines *H* as a function of the remaining parameters. Considering $a_i$, $i=1,2,3,4$ as fixed parameters one is left with three free parameters *u*, *v*, *z*.

In the case $C=C_{0,2}$ with two poles of third order at 0 and $\infty$, one could similarly consider meromorphic quadratic differentials of the form

2.7 $$\begin{aligned} q_\hbar (x) =\frac{\Lambda ^2}{x^3} + \frac{U}{x^2} + \frac{\Lambda ^2}{x} -\hbar \, Q_u(x)v +\frac{3}{4} \ \frac{\hbar ^2}{\left( x - u\right) ^2} ~, \end{aligned}$$

where $Q_u(x)(dx)^2$ is a quadratic differential on $C_{0,2}$ having a simple pole with residue equal to one at $x=u$, and double poles at 0 and $\infty$. This determines $Q_u(x)$ uniquely up to addition of terms of the form $\delta /x^2$. One may fix this freedom by demanding that $\lim _{x\rightarrow u}(Q_u(x)-\frac{1}{x-u})=0$. The function $Q_u(x)$ uniquely determined by these conditions can be represented as $Q_u(x)=\frac{ u(2x - u) }{x^2 (x- u) }=\frac{ 1}{x- u}-\frac{x-u}{x^2}$. The equation $v^2=q_u$ characterising an apparent singularity at $x=u$ is then equivalent to

2.8 $$\begin{aligned} v^2 =\frac{\Lambda ^2}{u^3} + \frac{U}{u^2} + \frac{\Lambda ^2}{u} . \end{aligned}$$

In this way, one arrives at a standard form for the function $q_\hbar (x)$ representing the quantum curve under consideration,

2.9 $$\begin{aligned} q_\hbar (x) = \frac{\Lambda ^2}{x^3} + \frac{U(u,v)}{x^2} +\frac{\Lambda ^2}{x} \, - \hbar \ \frac{ u \ \left( 2x - u \right) }{x^2 \left( x- u\right) } \ v \, + \frac{3}{4} \ \frac{\hbar ^2}{\left( x - u\right) ^2} ~, \end{aligned}$$

with *U*(*u*, *v*) being determined by (2.8). In total one is left with three free parameters $u,v,\Lambda$. The parameter $\Lambda$ takes the role of the complex structure parameter *z* of $C_{0,4}$.

It is worth noting that the terms which are regular at $x=u$ are $\hbar$-independent in the parameterisation (2.9). From the point of view of quantisation of the classical curve $\Sigma$, there is no obvious reason to exclude corrections to (2.9) which are higher order in $\hbar$. One may, in particular, consider the possibility to replace *v* with a function $v(\hbar )$ having a convergent power series expansion $v(\hbar )=\sum _{k=0}^\infty v_k\hbar ^k$. This would introduce an infinite set $\{v_k;0\le k \in {\mathbb {Z}}\}$ of new parameters. However, the generality gained by this replacement appears to be inessential in the sense that one can always reach the standard form (2.9) by a $\hbar$-dependent change of parameters $v=v(\hbar )$. We will see shortly, on the other hand, that alternative choices of parameters for the quantum curves can be useful. The choice of a possibly $\hbar$-dependent parameterisation of the quantum curves can be interpreted as a choice of a particular quantisation scheme.

### Representing Quantum Curves in Terms of Holomorphic Connections

It is also useful to recall that opers with apparent singularities can be related to generic holomorphic $sl_2 ({\mathbb {C}})$ $\hbar$-connections, in a local trivialisation represented as

2.10 $$\begin{aligned} \nabla _\hbar = dx\left( \hbar \partial _x - A (x)\right) ~,\quad \text {where}~~ A (x) = \left( \begin{matrix} A_0 & \quad A_+ \\ A_- & \quad -A_0 \end{matrix} \right) ~. \end{aligned}$$

by means of a gauge transformation which is branched at the zeros of $A_-(x)$,

2.11 $$\begin{aligned} \nabla _\text {Op} = h^{-1} \cdot \nabla \cdot h = dx \left( \hbar \partial _x - \left( \begin{matrix} 0 & \quad q_\hbar (x) \\ 1 & \quad 0 \end{matrix} \right) \right) ~ . \end{aligned}$$

A gauge transformation *h* which achieves this can be represented as

2.12 $$\begin{aligned} h=\bigg (\begin{matrix} 1/\sqrt{A_-} & \quad 0\\ 0 & \quad \sqrt{A_-}\end{matrix}\bigg )\left( \begin{matrix} 1 & \quad \alpha \\ 0 & \quad 1\end{matrix}\right) , \quad \alpha (x)=\frac{\hbar }{2}\frac{\partial _xA_-}{A_-} + A_0(x). \end{aligned}$$

The function $q_\hbar (x)$ representing the only nontrivial matrix element in (2.11) is found to be

2.13 $$\begin{aligned} q_\hbar (x) = A_0^2+A_+A_- - \hbar \left( A_0' - \frac{A_0A_-'}{A_-}\right) + \hbar ^2\bigg (\frac{3}{4}\left( \frac{A_-'}{A_-}\right) ^2- \frac{A_-''}{2A_-}\bigg ) ~. \end{aligned}$$

For $C=C_{0,n}={\mathbb {P}}^1\setminus \{z_1,\dots ,z_{n-1},\infty \}$ one considers connections of the form

2.14 $$\begin{aligned} A(x)=\sum _{r=1}^{n-1}\frac{A_r}{x-z_r}. \end{aligned}$$

In the case $C=C_{0,2}$ with two irregular singularities of the type introduced above, we may consider a connection of the form

2.15 $$\begin{aligned} A(x)= \left( \begin{array}{cc} \frac{w}{x } & \quad -\Lambda ^2 \left( \frac{1}{ux }-1\right) \\ \frac{1}{x}\left( 1 - \frac{u}{x} \right) & \quad - \frac{w}{x } \\ \end{array} \right) ~, \end{aligned}$$

The resulting function $q_\hbar (x)$ can be brought into the standard form (2.9) by relating the parameters, respectively, as

2.16 $$\begin{aligned} U= w^2- \Lambda ^2 (u+u^{-1}) ~, \qquad v\equiv v(w) = -\frac{w}{u} - \frac{\hbar }{u}. \end{aligned}$$

In the case $C=C_{0,4}$, it can also be shown by explicit computations (see, for instance, Appendix A in [35] for further details) that all quantum curves of the form (2.6) can be obtained from holomorphic $\hbar$-connections by singular gauge transformations of the type considered above.

### Representing Quantum Curves Through the Riemann–Hilbert Problem

Having recalled the relation between quantum curves and holomorphic $\hbar$-connections, this brings us naturally to a third and especially useful way to represent quantum curves, through the Riemann–Hilbert correspondence. The Riemann–Hilbert problem is to find a multivalued analytic matrix function $\Psi (y)$ on *C* having an analytic continuation $(\gamma .\Psi )(y)$ along $\gamma \in \pi _1(C)$ represented in the form $(\gamma .\Psi )(y)=\Psi (y)\rho (\gamma )$ for a given representation $\rho :\pi _1(C)\rightarrow \textrm{GL}(2,{{\mathbb {C}}})$. The connection to the representation of quantum curves discussed in Sect. 2.3 comes through the observation that a solution $\Psi (y)$ to the Riemann–Hilbert problem defines a holomorphic connection on *C* as

2.17 $$\begin{aligned} A(y)=(\partial _y\Psi (y))(\Psi (y))^{-1}. \end{aligned}$$

In the case $C=C_{0,n}$, we may take closed curves $\gamma _r$ around $y=z_r$ as generators for $\pi _1(C)$. In this case, we will consider the cases where the matrices $M_r=\rho (\gamma _r)$ are diagonalizable, $M_r={\textsf{C}}_r^{-1}e^{2\pi \textrm{i}{\textsf{D}}_r}{\textsf{C}}_r$, for a fixed choice of diagonal matrices ${\textsf{D}}_r$.

The solution to this problem is unique up to left multiplication with single valued matrix functions. In order to fix this ambiguity, we need to specify the singular behaviour of $\Psi (y)$ at $y=z_r$, leading to the following refined version of the Riemann–Hilbert problem:

*Find a matrix function* $\Psi (y)$ *such that the following conditions are satisfied.*

*i)*: $\Psi (y)$ *is multivalued, analytic and invertible on* $C_{0,n}$, *and satisfies* $\Psi (y_0)\,=\,1\,$.
*ii)*: $\Psi (y)$ *can be represented in the neighbourhood of* $z_k$ *in the form*
  2.18 $$\begin{aligned} \Psi (y)\,=\,F^{(k)}(y)\cdot (y-z_k)^{{\textsf{D}}_k}\cdot {\textsf{C}}_k\,,\qquad k=1,\dots ,n, \end{aligned}$$
  *with* $F^{(k)}(y)$ *holomorphic and invertible at* $y=z_k$, ${\textsf{C}}_k\in \textrm{GL}(2,{{\mathbb {C}}})$, *and* ${\textsf{D}}_k$ *being diagonal matrices for* $k=1,\dots ,n$.

It is known that generic representations $\rho :\pi _1(C_{0,n})\rightarrow \textrm{GL}(2,{{\mathbb {C}}})$ can be realised as monodromy representation of such a Fuchsian system, which means that a solution to the Riemann–Hilbert problem formulated above will generically exist.

If a solution $\Psi (y)$ to the Riemann–Hilbert problem formulated above exists, it is for fixed positions $z_1,\dots ,z_n$ uniquely determined by the monodromy data ${\textsf{C}}=({\textsf{C}}_1,\dots ,{\textsf{C}}_n)$ and the diagonal matrices ${\textsf{D}}=({\textsf{D}}_1,\dots ,{\textsf{D}}_n)$. Changes of the positions $z_1,\dots ,z_n$ lead to a change of $\Psi (y)$ that can be represented by a system of partial differential equations as follows. One may deduce from *ii)* that the variations of $\Psi (y)$ with respect to $z_r$ with fixed ${\textsf{C}}$, ${\textsf{D}}$ can be represented as

2.19 $$\begin{aligned} \partial _{z_r} \Psi (y)=A_r(y)\Psi (y),\qquad A_r(y):=\,-\frac{A_r}{y-z_r} ~. \end{aligned}$$

The consistency of the differential equations (2.19) for different values of *r*, and the consistency with (2.17) imply nonlinear partial differential equations for the matrices $A_r$ known as the Schlesinger equations. Integrating (2.19) allows one to represent a finite variation of the positions $z_1,\dots ,z_n$ as a gauge transformation acting on $\Psi (y)$ from the left.

Representing quantum curves in terms of $\Psi$ through the Riemann–Hilbert correspondence offers a convenient way to represent the Schlesinger system in Hamiltonian form. The Schlesinger equations represent Hamiltonian flows generated by the Hamiltonians

2.20 $$\begin{aligned} H_r:=\frac{1}{2}\;\underset{x=z_r}{\textrm{Res}}\operatorname {tr}A^2(x)=\sum _{s\ne r} \frac{{\textrm{tr}}(A_rA_s)}{z_r-z_s}\,, \end{aligned}$$

using the Poisson structure

2.21 $$\begin{aligned} \big \{\,A\left( x\right) \,\begin{array}{c} \otimes \\ , \end{array}\,A\left( x'\right) \,\big \}\,=\, \left[ \,\frac{{\mathcal {P}}}{x-x'}\,,\,A\left( x\right) \otimes 1+1\otimes A\left( x'\right) \,\right] , \end{aligned}$$

where ${\mathcal {P}}$ denotes the permutation matrix. The generating function of these time-dependent hamiltonians is the isomonodromy tau function, defined by the equation

2.22 $$\begin{aligned} H_r = \partial _{z_r} \log {{{\mathcal {T}}}}(\mu , {\textbf{z}}) ~\,, \end{aligned}$$

whereas $\mu$, the monodromy data, acquires the role of integrals of motion. An important property of this definition is that the normalisation of ${{{\mathcal {T}}}}$ is ambiguous, admitting a rescaling by arbitrary functions of $\mu$.

All this has a well-known analog in the case $C=C_{0,2}$ considered above. The Schlesinger system will be replaced by the Painlevé III equation in this case.^3^

### Free Fermion Partition Functions as Tau-Functions

CFT offers a useful framework for describing how to associate free fermion partition functions to quantum curves. We will now quickly review and generalise the corresponding discussion in [36] before we specialise to another instructive example, related to the Painlevé III equation. Let us first recall the general framework underlying the relation between free fermions and tau functions. This relation hinges on two key facts: (i) a solution $\Psi$ to the Riemann–Hilbert problem determines a certain state ${\mathfrak {f}}_\Psi$ in the free fermion Fock space and (ii) the state ${\mathfrak {f}}_\Psi$ is characterized by certain relations taking the form of conformal Ward identities, leading to a direct connection with conformal blocks.

Consider a system of *N* free fermions (useful background suitable for our purposes can be found in [36, 111])

2.23 $$\begin{aligned} \begin{aligned} \psi (z) = \sum _{n\in {\mathbb {Z}}} \psi _n \, z^{-n-1}&\qquad {\bar{\psi }}(z) = \sum _{n\in {\mathbb {Z}}} {\bar{\psi }}_n \, z^{-n} \\ \psi _n = (\psi _{1,n},\dots , \psi _{N,n})&\qquad {\bar{\psi }}_n = (\bar{\psi }_{1,n},\dots , {\bar{\psi }}_{N,n})^T \end{aligned} \end{aligned}$$

and let ${\mathcal {F}}$ denote the Fock space built from a highest weight vector ${\mathfrak {f}}_0$.

A solution $\Psi$ to the Riemann–Hilbert problem on *C* defines a state ${\mathfrak {f}}\in {\mathcal {F}}$ as follows. Let *P* be a point on *C* where $\Psi$ is non-singular, and let 1/*x* be a local coordinate in a neighbourhood of *P* vanishing at *P*. Let $A_{kl}$ be the $N\times N$ matrices defined by the expansion

2.24 $$\begin{aligned} G_\Psi (x,y) = \frac{\Psi (x)^{-1} \cdot \Psi (y)}{x-y} = \frac{1}{x-y} + \sum _{l\ge 0}\sum _{k>0} y^{-l-1} x^{-k} \, A_{kl} \,. \end{aligned}$$

These uniquely define a state in the Fock space

2.25 $$\begin{aligned} {\mathfrak {f}}_\Psi = N_\Psi \, \exp \left( -\sum _{k>0}\sum _{l\ge 0} \psi _{-k} \cdot A_{kl}\cdot {\bar{\psi }}_l \right) \, {\mathfrak {f}}_0 \end{aligned}$$

establishing a map $\Psi \mapsto {\mathfrak {f}}_\Psi$ that determines a state uniquely up to normalisation $N_\Psi \in {\mathbb {C}}$..

By construction $G_\Psi$ is the $N\times N$ matrix-valued correlator

2.26 $$\begin{aligned} \frac{\langle {\mathfrak {f}}_0 , {\bar{\psi }}(x) \psi (y) \, {\mathfrak {f}}_\Psi \rangle }{\langle {\mathfrak {f}}_0 , {\mathfrak {f}}_\Psi \rangle } = G_\Psi (x,y)\,. \end{aligned}$$

where $\langle \,\cdot \,, \,\cdot \,\rangle$ denotes the natural pairing ${\mathcal {F}}^*\otimes {\mathcal {F}}\rightarrow {\mathbb {C}}$ induced by expectation values in CFT. Notice that fixing *x* and varying *y* around punctures of *C* induces the right-monodromy action on $G_\Psi$ prescribed by the Riemann–Hilbert problem for $\Psi (y)$. Similarly, fixing *y* and varying *x* induces an (inverse) left-monodromy action for $\Psi (x)^{-1}$. On the other hand, $G_\Psi$ also has a pole with trivial monodromy at $x=y$.

The state ${\mathfrak {f}}_\Psi$ characterized in this way by $G_\Psi$ satisfies Ward identities of the free fermion VOA

2.27 $$\begin{aligned} \left( \frac{1}{2\pi \textrm{i}} \oint _{{\mathcal {C}}} \psi (z) \cdot {\bar{g}}(z) \right) {\mathfrak {f}}_\Psi = 0 \qquad \left( \frac{1}{2\pi \textrm{i}} \oint _{{\mathcal {C}}} g(z) \cdot {\bar{\psi }}(z) \right) {\mathfrak {f}}_\Psi = 0 \end{aligned}$$

where *g*(*z*) and ${\bar{g}}(z)$ are row and column vectors of the form

2.28 $$\begin{aligned} g(z) = v(z) \cdot \Psi (z) \qquad {\bar{g}}(z) = \Psi (z)^{-1}\cdot \bar{v}(z) \end{aligned}$$

and $v(z), {\bar{v}}(z)$ are row and column vectors of arbitrary meromorphic functions having poles only at $z=\infty$. It follows that $g(z), {\bar{g}}(z)$ solve the monodromy constraints imposed by the Riemann–Hilbert problem at punctures, but may have poles at infinity.

Conditions (2.27) define the fermion VOA conformal blocks, and uniqueness of ${\mathfrak {f}}_\Psi$ implies that the vector space of the VOA conformal blocks has dimension one. On the other hand, the free fermion extended symmetry includes in particular a Virasoro subalgebra generated by the modes $L_n$ of the energy–momentum tensor $T(x)=\sum _{n\in {{\mathbb {Z}}}}x^{-n-2}L_n$ constructed as a bilinear expression of the free fermion fields. It follows that free fermion conformal blocks are special conformal blocks for the Virasoro algebra.

Taking $C=C_{0,n}$ a sphere with *n* punctures, we may consider variations of the complex structure on *C* parameterized by variation of positions of punctures $z_r$. Virasoro conformal blocks are defined to be flat sections of a connection on a vector bundle over ${\mathcal {M}}_C$, the moduli space of complex structures on *C*, defined by a parallel transport equation of the form [54]

2.29 $$\begin{aligned} \left( \partial _{z_r} - {\textsf{H}}_r \right) \, {\mathfrak {f}}_\Psi ({\textbf{z}}) = 0, \end{aligned}$$

with operators ${\textsf{H}}_r$ represented by specific linear combinations of Virasoro generators. The space of free fermion VOA conformal blocks is preserved by this parallel transport, defining a line sub-bundle with fibre spanned by ${\mathfrak {f}}_\Psi$.

The chiral partition functions $Z_C(\Psi )=\langle {\mathfrak {f}}_0 , {\mathfrak {f}}_\Psi  \rangle$ associated with a flat section ${\mathfrak {f}}_\Psi$ of the connection defined in (2.29) will for $C=C_{0,n}$ satisfy the differential equation

2.30a $$\begin{aligned} \left( \sum _{r=1}^n\left( \frac{\Delta _r}{(x-z_r)^2}+\frac{1}{x-z_r}\frac{\partial }{\partial z_r}\right) \!\right) Z_C(\Psi ) =\langle \!\langle T(x)\rangle \!\rangle _\Psi   \,Z_C(\Psi ), \end{aligned}$$

with $\langle \!\langle T(x)\rangle \!\rangle _\Psi$ being the normalised expectation value of the energy–momentum tensor,

2.30b $$\begin{aligned} \langle \!\langle T(x)\rangle \!\rangle _\Psi   = \frac{\langle {\mathfrak {f}}_0 , T(x) {\mathfrak {f}}_\Psi  \rangle }{\langle {\mathfrak {f}}_0 , {\mathfrak {f}}_\Psi  \rangle }. \end{aligned}$$

Finally, it can be shown using Ward identities and the above definition of ${\mathfrak {f}}_\Psi$, that the normalized expectation value of the energy–momentum tensor is related to the generating function $\textrm{tr}(A^2)$ of the isomonodromic deformation Hamiltonians as follows:

2.30c $$\begin{aligned} \langle \!\langle T(x)\rangle \!\rangle = \textrm{tr}\big (A^2(x)\big )=\sum _{r=1}^n\left( \frac{\Delta _r}{(x-z_r)^2}+\frac{H_r}{x-z_r}\right) . \end{aligned}$$

It then follows immediately from (2.30) that the free fermion partition function satisfies $\frac{\partial }{\partial z_r}Z_C(\Psi )=H_r Z_C(\Psi )$, relating $Z_C(\Psi )$ to the isomonodromic tau-function ${{\mathcal {T}}}({\textbf{z}})$.

By considering the collision limit of (2.30) one finds in the case of Painlevé III

2.31 $$\begin{aligned} \Lambda \frac{\partial }{\partial \Lambda }Z_C(\Psi )= 4\,U\, Z_C(\Psi ). \end{aligned}$$

This is equivalent to the defining equation for the tau-function of Painlevé III (see e.g. [61]).

Equations (2.30) do not entirely fix the relation between free fermion partition functions and the tau function. There are in fact two important problems that are left open by the above discussion. The first is to find the precise relation between the variables $(\xi ,a)$ that appear in the free fermion partition functions $Z_{\textrm{ff}}(\xi ,a;\hbar )$, and the parameters of the quantum curves. One may furthermore note that the relation between $Z_{\textrm{ff}}$ and the isomonodromic tau-function only fixes the dependence on the variables ${\textbf{z}}=(z_1,\dots ,z_n)$. This leaves the problem open how to fix the dependence on the monodromy data $\mu$. We will soon see that the variables $(\xi ,a)$ are naturally identified with suitable functions of the monodromy data.

## Theta Series

As summarised in the introduction, our starting point is the observation [36] that there exists a small family of normalisations for the tau-functions of Painlevé VI distinguished by the property that the normalised tau-functions admit series expansions having the form (1.1). The variables $\xi$ and *a* appearing in this expansion can be identified with certain special coordinates on the moduli space ${{\mathcal {M}}}_{{\textrm{ch}}}(C_{0,4})$ of flat connections on the Riemann surface $C_{0,4}$.

In order to gain insight on how to generalise this definition of normalised tau-functions to other Riemann surfaces it turns out to be instructive to compare the resulting picture to the case of the two-punctured sphere $C=C_{0,2}$ with two irregular singularities of the type defined in the previous section. The equation describing isomonodromic deformations of holomorphic $\textrm{SL}(2)$-connections is then equivalent to the Painlevé III equation. Using results of [61, 82], we will again find a correspondence between normalised tau-functions having expansions of theta series form and certain coordinates on the space of monodromy data. This example will reveal a new feature. There can exist theta series expansions of the tau-function for Painlevé III in which the role of the Fenchel–Nielsen-type coordinates is taken by Fock–Goncharov coordinates. This suggests that it is natural to consider both types of coordinates for the purpose of defining fully normalised tau-functions.

### Fenchel–Nielsen-Type Coordinates

As a preparation, we shall now review a definition of Fenchel–Nielsen (FN)-type coordinates based on the gluing construction of Riemann surfaces. Our main goal is to describe the choices entering into the definition of these coordinates in the explicit form that will be used later.

#### Gluing

Let $C_i$, $i=1,2$, be two Riemann surfaces with punctures $P_i$ embedded in discs $D_i$ having coordinates $x_i$ vanishing at $P_i$ such that $D_i=\{Q_i\in C;0<|x_i(Q_i)|<1\}$, for $i=1,2$, respectively. Out of these data one may define families of Riemann surfaces $C_z$, $z\in {\mathbb {C}}$ being a complex parameter, by identifying pairs of points $(Q_1,Q_2)$ in the annuli $A_i=\{Q_i\in D_i;|z|<|x_i(Q_i)|<1\}$ satisfying $x_1(Q_1)x_2(Q_2)=z$ to get an annulus $A\subset C_z$ with coordinate $x\equiv x_2$.

It is easy to extend the gluing operation to punctured surfaces $C_i$ with flat bundles. Let $\rho _i:\pi _1(C_i)\rightarrow \textrm{SL}(2,{\mathbb {C}})$ be representations of $\pi _1(C_i)$ characterising flat bundles on $C_i$ for $i=1,2$, respectively. We will only consider representations $\rho _i$ having the property that the holonomies around $P_i$ represented by the diagonal matrices $\textrm{diag}(e^{2\pi \textrm{i}\sigma _i},e^{-2\pi \textrm{i}\sigma _i})$ with $\sigma _2=-\sigma _1\equiv \sigma -\frac{1}{2}$. For generic $\rho _i$ we may characterise the flat bundles by the corresponding solutions to the Riemann–Hilbert problems, concretely represented by matrix-valued function $\Psi _i(Q_i)$ on $D_i$ having for each $\gamma \in \pi _1(C_i)$ an analytic continuation $(\gamma .\Psi )(Q_i)$ satisfying $(\gamma .\Psi )(Q_i)=\Psi (Q_i)\rho _i(\gamma )$. The coordinates $x_i$ allow us to represent the functions $\Psi _i$ by functions $\Phi _i(x_i)$ on the punctured disc $D=\{x\in {\mathbb {C}};|x|<1\}$. It will for our goals be sufficient to consider only the case that $P_i$ is a regular singularity of the form $\Phi _i(x_i)=F_i(x_i)x_i^{{\textsf{d}}_{i}}$, with ${\textsf{d}}_i=\textrm{diag}(\sigma _i,-\sigma _i)$, and $F_i(x_i)$ holomorphic at $x_i=0$. The gluing condition $x_1x_2=z$ furthermore allows us to represent the functions $\Phi _i(x_i)$ by functions $\Xi _i(x)$ on the annulus *A*, for $i=1,2$, and where *x* is a local coordinate on *A*. One may then define a flat bundle on *C* by imposing the relation

3.1 $$\begin{aligned} \Xi _2(x)=\Xi _1(x) T_A,\qquad T_A\in \textrm{SL}(2,{\mathbb {C}}). \end{aligned}$$

Assuming that $\Xi _i(x)$ both have diagonal monodromy around *A* for $i=1,2$ one generically has to require that $T_A$ is diagonal, and can therefore be represented as $T_A=\textrm{diag}(e^{-\pi \textrm{i}\eta }, e^{\pi \textrm{i}\eta })$.

The parameters $(\sigma ,\eta )$ introduced in this way define coordinates for the character variety ${\mathcal {M}}_{{\textrm{ch}}}(C)$. It is clear that the origin of the coordinate $\eta$ depends on the choice of the functions $\Xi _i$, $i=1,2$. Multiplying either $\Xi _1$ or $\Xi _2$ by a diagonal matrix from the right will shift $\eta$. Fixing a choice for $\Xi _1$ and $\Xi _2$ determines the coordinate $\eta$ uniquely.

The gluing construction described in this section can be used to introduce a pair of coordinates $(\sigma ,\eta )$ for any Riemann surface *C* that can be represented by gluing two surfaces $C_1$ and $C_2$. Such coordinates will be called coordinates of Fenchel–Nielsen (FN) type in the following. It has been noted in Sect. 3.1.1 that the precise definition of the coordinate $\eta$ depends on some choices. We are now going to discuss two useful ways to fix these choices.

#### Normalisation Factors

One may recall, on the one hand, that $\eta$ can be defined by fixing a pair of functions $\Phi _i(x)$ representing the solutions of the Riemann–Hilbert problem on $C_i$ in the discs $D_i$. Given that $\Phi _i(x)$ have a regular singularity at $x=0$ for $i=1,2$, we may fix the freedom to multiply $\Phi _i(x)$ by constant diagonal matrices from the right by imposing the condition

3.2 $$\begin{aligned} \Phi _i(x_i)= \bigg (\,\begin{matrix} {\tilde{\xi }}_+^{(i)}(x_i) & \quad {\tilde{\xi }}_-^{(i)}(x_i) \\ \xi _+^{(i)}(x_i) & \quad \xi _-^{(i)}(x_i)\end{matrix}\,\bigg )\,, \qquad \xi _\pm ^{(i)}(x_i)=\nu _\pm ^{(i)}\, x_i^{{\textsf{d}}_i}\, (1+{{\mathcal {O}}}(x_i))\,. \end{aligned}$$

Choosing for each $i=1,2$ a pair $(\nu _+^{(i)},\nu _-^{(i)})$ of complex numbers fixes the freedom to multiply $\Phi _i(x_i)$ by constant diagonal matrices from the right. The residual gauge freedom to multiply $\Phi _i(x_i)$ from the left by matrix valued holomorphic functions of $x_i$ can be fixed, for example, by demanding that ${\tilde{\xi }}_s^{(i)}(x_i)=\partial _{x_i}\xi _s^{(i)}(x_i)$, which is equivalent to demanding that the connection one-forms $A_i(x_i)=(\partial _x\Phi _i(x_i))(\Phi _i(x_i))^{-1}$ have oper form. It is then natural to further reduce the normalisation freedom by imposing the condition $\textrm{det}(\Phi _i(x_i))=1$ for $i=1,2$. This implies $(2\sigma -1)\nu _+^{(i)}\nu _-^{(i)}=1$. The left-over normalisation freedom is then parameterised by the normalisation factors^4^

3.3 $$\begin{aligned} {\textsf{n}}^{(1)}:=\frac{\nu _-^{(1)}}{\nu _+^{(1)}}\,, \qquad {\textsf{n}}^{(2)}:=\frac{\nu _+^{(2)}}{\nu _-^{(2)}}\,. \end{aligned}$$

We may thereby use the complex numbers ${\textsf{n}}^{(i)}$ as parameters allowing us to distinguish different choices of the FN coordinate $\eta$ corresponding to the same pants decomposition. A distinguished choice is to consider ${\textsf{n}}^{(1)} ={\textsf{n}}^{(2)} = 1$, which we will denote by $\eta _0$. Then, from (3.1) one may easily deduce that other choices of normalisation lead to a coordinate $\eta$ related to $\eta _{0}$ as follows

3.4 $$\begin{aligned} {e^{2\pi \textrm{i}\eta _0 }}=e^{2\pi \textrm{i}\eta }\,{{\textsf{n}}^{(2)}{\textsf{n}}^{(1)}}\,. \end{aligned}$$

The direct relation between choices of normalisation for the solutions (3.2) and choices of coordinates $\eta$ expressed in (3.4) will often be used in the following.

#### Trace Functions

The traces of holonomies $\textrm{tr}(\rho (\gamma ))$, $\gamma \in \pi _1(C)$ generate the ring of algebraic functions on the character variety. The relations in $\pi _1(C)$ together with identities satisfied by traces of $SL(2,{{\mathbb {C}}})$-matrices imply algebraic relations between the trace functions. In the case $C=C_{0,4}$, for example, one can consider the seven trace functions

3.5a $$\begin{aligned}&L_k=\operatorname {Tr} M_k=2\cos 2\pi \theta _k,\qquad k=1,\ldots ,4, \end{aligned}$$

3.5b $$\begin{aligned}&L_s=\operatorname {Tr} M_1 M_2,\qquad L_t=\operatorname {Tr} M_1 M_3,\qquad L_u=\operatorname {Tr} M_2 M_3, \end{aligned}$$

generating the algebra of invariant polynomial functions on ${{\mathcal {M}}}_{{\textrm{ch}}}(C_{0,4})$, with $M_k$ being the monodromy around $z_k$, for $k=1,2,3,4$. These trace functions satisfy the quartic equation

3.6 $$\begin{aligned}&L_1L_2L_3L_4+L_sL_tL_u+L_s^2+L_t^2+L_u^2+L_1^2+L_2^2+L_3^2+L_4^2\nonumber \\&\quad =\left( L_1L_2+L_3L_4\right) L_s+\left( L_1L_3+L_2L_4\right) L_t +\left( L_2L_3+L_1L_4\right) L_u+4. \end{aligned}$$

FN-type coordinates can alternatively be defined by picking a certain parameterisation of the trace functions solving the algebraic relations they satisfy. We will now explain how the definition of FN-type coordinates by means of the gluing construction induces a parameterisation of the trace functions.

Considering surfaces *C* represented by gluing surfaces $$C_1$$ and $$C_2$$ as described in Sect. 3.1.1, one may find a set of generators for $$\pi _1(C)$$ by picking sets of generators for $$\pi _1(C_i)$$, $$i=1,2$$, and adding sufficiently many curves $$\gamma $$ traversing the annulus *A* created in the gluing construction. It suffices to consider curves $$\gamma $$ which can be represented as a composition $$\varpi _2 \circ \varpi _A \circ \varpi _1 \circ \varpi _A^{-1}$$, where $$\varpi _i$$ are paths supported in $$C_i$$, for $$i=1,2$$, respectively, and $$\varpi _A$$ is a path traversing the annulus *A* from $$C_2$$ to $$C_1$$. The holonomy $$M_{\gamma }$$ can then be factorised in the form $$T^{-1}_AM_1 T_A M_2 $$, with $$M_i$$ being the holonomies along $$\varpi _i$$, for $$i=1,2$$, respectively, and $$T_A$$ being the transition function across *A*. For generic $$\rho $$, one may diagonalise simultaneously both the holonomy $$M_A=-\left( {\begin{smallmatrix} e^{2\textrm{i}\pi \sigma } &  0 \\ 0 &  e^{-2\textrm{i}\pi \sigma } \end{smallmatrix}}\right) $$ around the non-contractible cycle of the annulus, and the matrix $$T_A=\left( {\begin{smallmatrix} e^{\textrm{i}\pi \eta } &  0 \\ 0 &  e^{-\textrm{i}\pi \eta } \end{smallmatrix}}\right) $$ describing the parallel transport from one boundary of the annulus to the other. The variables $$(\sigma ,\eta )$$ defined in this way can be used to complete coordinate systems for the moduli spaces of flat connections on $$C_i$$, $$i=1,2$$, to a coordinate system for the moduli space of flat connections on *C*.

One may note that the trace functions $\textrm{tr}(M_{\gamma }) =\textrm{tr}(T^{-1}_AM_1 T_A M_2 )$ will then have a simple $\eta$-dependence of the form

3.7 $$\begin{aligned} \begin{aligned}&\textrm{tr}(M_{\gamma })=t^+(\sigma )e^{2\pi \textrm{i} \eta }+t^0(\sigma )+t^-(\sigma )e^{-2\pi \textrm{i} \eta },\\&t^+(\sigma )=b_2 c_1, \quad t^0(\sigma )= a_1 a_2 + d_1 d_2,\quad t^-(\sigma )= b_1 c_2,\end{aligned} \qquad M_i=\bigg (\begin{matrix} a_i &  b_i \\ c_i &  d_i\end{matrix}\bigg ). \end{aligned}$$

Fixing a particular definition of the coordinate $\eta$ is thereby seen to be equivalent to specifying the functions $t^{\pm }(\sigma )$ appearing in the expressions (3.7) for the trace functions. We will next illustrate this relation by some examples which will be used later.

#### Case $C=C_{0,4}$

We have just illustrated two ways to parametrise ambiguities in the definition of $\eta$: either in terms of normalisation of flat sections in the factorization limit of the Riemann–Hilbert correspondence, or by a specific parametrisation of trace functions. The two possibilities are of course related to each other, we will now explain this with the specific example of $C_{0,4}$.

In the case $C=C_{0,4}\simeq {\mathbb {P}}^1\setminus \{0,z,1,\infty \}$, one may express the solutions to the Riemann–Hilbert problem on the surfaces $C_i\simeq C_{0,3}\simeq {\mathbb {P}}^1{\setminus }\{0,1,\infty \}$, $i=1,2$, in terms of the Gauss hypergeometric function. The solution $\Phi _2$ to the Riemann–Hilbert problem on $C_2$ can be represented in the form (3.2) with

3.8 $$\begin{aligned} \begin{aligned} \xi _{s}^{(2)}(x)=\nu _s^{(2)}\, x^{s(\sigma -\frac{1}{2})}(1-x)^{s\theta _3}F(A_s,B_s,C_s;x), \end{aligned} \end{aligned}$$

for $s=\pm 1$, where *F*(*A*, *B*, *C*; *x*) is the Gauss hypergeometric function and

3.9 $$\begin{aligned} \begin{aligned} A_+=A, \quad A_-=1-A,\\ B_+=B, \quad B_-=1-B,\\ \end{aligned}\qquad \begin{aligned}&C_+=C,\\  &C_-=2-C, \end{aligned} \qquad \begin{aligned} A&=\theta _3+\theta _4+\sigma ,\\ B&=\theta _3-\theta _4+\sigma , \end{aligned}\qquad C=2\sigma . \end{aligned}$$

$\Phi _1$, on the other hand, may be chosen by defining $\xi _\pm ^{(1)}(x)$ from $\xi _\pm ^{(2)}(x)$ by the replacements $x\rightarrow x^{-1}$, $\theta _4\rightarrow \theta _1$, $\theta _3\rightarrow \theta _2$ and $s\rightarrow -s$.

As an example for the computation of trace functions let us then consider the trace $$\textrm{tr}(M_{z1})$$ of the holonomy around *z* and 1. According to the discussion in Sect. 3.1.3 we can represent $$M_{z1}$$ in the form $$T^{-1}_AM_1 T_A M_2 $$, with $$M_2$$ being the monodromy around $$x=1$$ on $$C_2$$ and $$M_1$$ being the monodromy around the point representing $$x=z$$ on $$C_1$$. The well-known formulae for the monodromies of the hypergeometric function then yield formulae for $$M_2$$,

3.10 $$\begin{aligned} M_2=\left( \begin{matrix} a_2 & \quad b_2 \\ c_2 & \quad d_2\end{matrix}\right) ,\qquad \begin{aligned}&b_2=- \, {\textsf{n}}^{(2)} \frac{2\pi \textrm{i}\,\Gamma (C_+)\Gamma (C_+-1)}{\Gamma (A_+)\Gamma (B_+)\Gamma (C_+-A_+)\Gamma (C_+-B_+)},\\&c_2=+ \frac{1}{{\textsf{n}}^{(2)}} \frac{2\pi \textrm{i}\,\Gamma (C_-)\Gamma (C_--1)}{\Gamma (A_-)\Gamma (B_-)\Gamma (C_--A_-)\Gamma (C_--B_-)}, \end{aligned} \end{aligned}$$

A similar formula for monodromy $M_1$ is found by the replacements defining $\xi _\pm ^{(1)}$ from $\xi _\pm ^{(2)}$.

Two choices for ${\textsf{n}}^{(i)}$, $i=1,2$, appear to be fairly natural. One may, on the one hand, simply choose ${\textsf{n}}^{(i)}=1$ for $i=1,2$. In that case we easily see that $t^{\pm }(\sigma )=t^{\pm }_0(\sigma )$ with

3.11 $$\begin{aligned} t^{\pm }_0(\sigma )&= \frac{(2\pi )^2\,\big [\Gamma (1\pm (2\sigma -1))\Gamma (\pm (2\sigma -1))\big ]^2}{ \prod _{s,s'=\pm 1}\Gamma \big (\frac{1}{2}\pm \big (\sigma -\frac{1}{2}\big )+s\theta _1+s' \theta _2\big )\Gamma \big (\frac{1}{2}\pm \big (\sigma -\frac{1}{2}\big )+s\theta _3+s' \theta _4\big )}. \end{aligned}$$

This corresponds to the definition of $\eta _0$ given in Sect. 3.1.3. The normalisation factors ${\textsf{n}}^{(i)}$, $i=1,2$, can alternatively be chosen such that $b_2c_1=1$, defining a coordinate $\eta$ such that

3.12 $$\begin{aligned} t^+(\sigma ) = 1,\quad \;\; t^{-}(\sigma )&=\prod _{s,s'=\pm 1} \frac{2\sin \pi (\sigma +s\theta _1+s' \theta _2)\,2\sin \pi (\sigma +s\theta _3+s' \theta _4)}{(2\sin 2\pi \sigma )^4}. \end{aligned}$$

One many now check that the coordinates $\eta$ and $\eta _0$ are related precisely as in (3.4), if ${\textsf{n}}^{(2)}$ is defined by choosing $b_2=1$ in (3.10), and ${\textsf{n}}^{(1)}$ defined by the replacements above.

### Normalised Tau-Functions for $C=C_{0,4}$

Series expansions for the isomonodromic tau-functions of theta series type have been derived in [30, 57, 60, 80]. It is quite remarkable that these expansions can be represented in a form resembling the series defining the theta-functions,

3.13 $$\begin{aligned}&{{\mathcal {T}}}^{(\eta )}(\,\sigma ,\eta \,,\,{\underline{\theta }}\,;\,z\,)=\sum _{n\in {{\mathbb {Z}}}} \,e^{2\pi \textrm{i}n\,\eta }\, {{\mathcal {N}}}^{(\eta )}(\sigma +n\,;\,{\underline{\theta }}\,){{\mathcal {F}}} (\,\sigma +n\,,\,{\underline{\theta }}\,;\,z\,), \end{aligned}$$

with functions ${{\mathcal {F}}}$ represented by power series of the form

$$\begin{aligned} {{\mathcal {F}}} (\,\sigma ,\,{\underline{\theta }};\,z\,)=z^{\sigma ^2-\theta _1^2-\theta _2^2}\bigg (1+\sum _{k=1}^\infty {{\mathcal {F}}}_k(\,\sigma ,\,{\underline{\theta }}\,) \, z^k\bigg ). \end{aligned}$$

As observed in [36], there is a fairly small family of distinguished choices for the coordinate $\eta$ for which one can recast the series expansions for the tau-functions in the form (3.13). We anticipate in the notations that the choice of functions ${{\mathcal {N}}}^{(\eta )}$ is related to the choice of $\eta$. The function ${{\mathcal {N}}}^{(\eta )}$ corresponding to the coordinate $\eta$ previously defined in Sect. 3.1.4 is

3.14a $$\begin{aligned}&{{\mathcal {N}}}^{(\eta )}(\,\sigma \,,{\underline{\theta }}\,)= {N(\sigma ,\theta _2,\theta _1)N(\sigma ,\theta _3,\theta _4)} \end{aligned}$$

3.14b $$\begin{aligned}&N(\vartheta _1,\vartheta _2,\vartheta _3) = \frac{ \prod _{\epsilon ,\epsilon ' = \pm } G(1+\vartheta _{1}+\epsilon \vartheta _{2}+\epsilon ' \vartheta _{3}) }{(2\pi )^{\vartheta _1}G(1) \prod _{r=1}^{3}G(1+2\vartheta _r) }\,, \end{aligned}$$

with *G*(*x*) being the Barnes G-function satisfying $G(x+1)=\Gamma (x)G(x)$. It is straightforward to show how to derive this form of the expansion from the results of [80] (cf. Appendix B [35]).

In order to clarify how ${{\mathcal {N}}}^{(\eta )}$ varies with the choice of $\eta$ it will be useful to note that (3.13) is equivalent to an expansion for the normalised tau-function of the following form

3.15 $$\begin{aligned} {\mathcal {T}}^{(\eta _0)}\big (\,\sigma ,\eta \,;\,{\underline{\theta }}\,;\,z\big )= \sum _{n\in {{\mathbb {Z}}}} \,e^{2\pi \textrm{i}n\,\eta _0 }\,{{\mathcal {C}}}_n(\,\sigma \,,{\underline{\theta }}\,) {{\mathcal {F}}} (\,\sigma +n\,,\,{\underline{\theta }}\,;\,z\,), \end{aligned}$$

with $\eta _0$ being the FN-type coordinate defined in Sect. 3.1.2, and ${{\mathcal {C}}}_n(\sigma ,{\underline{\theta }})$, $n\in {{\mathbb {Z}}}$, being a family of functions which can be represented in terms of a single function ${{\mathcal {N}}}^{(\eta )}(\sigma ,{\underline{\theta }})$ as

3.16 $$\begin{aligned} {{\mathcal {C}}}_n(\,\sigma \,,{\underline{\theta }}\,)= \left( \frac{{{\mathcal {N}}}^{(\eta )}(\sigma -1\,;\,{\underline{\theta }}\,)}{{{\mathcal {N}}}^{(\eta )}(\sigma \,;\,{\underline{\theta }}\,)}\right) ^n \frac{{{\mathcal {N}}}^{(\eta )}(\sigma +n\,;\,{\underline{\theta }}\,)}{{{\mathcal {N}}}^{(\eta )}(\sigma \,;\,{\underline{\theta }}\,)}. \end{aligned}$$

Indeed, by observing that ${{\mathcal {N}}}^{(\eta )}(\sigma ,{\underline{\theta }})$ is related to the normalisation factors ${\textsf{n}}^{(i)}$ defining the coordinate $\eta$ through the identity^5^

3.17 $$\begin{aligned} \frac{{{\mathcal {N}}}^{(\eta )}(\sigma -1\,;\,{\underline{\theta }}\,)}{{{\mathcal {N}}}^{(\eta )}(\sigma \,;\,{\underline{\theta }}\,)}= \frac{1}{{\textsf{n}}^{(1)}{\textsf{n}}^{(2)}}, \end{aligned}$$

one may easily check the equivalence of (3.13) and (3.15) using the relation between $\eta$ and $\eta _0$ from (3.4), together with

3.18 $$\begin{aligned} {{\mathcal {T}}}^{(\eta )}={{\mathcal {N}}}^{(\eta )} \, {{\mathcal {T}}}^{(\eta _0)}\,. \end{aligned}$$

At this point, it should be stressed that while (3.13) has the form of a generalized theta-series expansion, (3.15) does not. Nevertheless the latter is somewhat canonical, in the sense that choosing coordinate $\eta _0$ corresponds to ${\textsf{n}}^{(i)} = 1$. Moreover the relation (3.16) provides an interesting way to find additional choices of $\eta$ inducing a theta-series expansion like (3.13). One may note, in fact, that the representation of the coefficient functions ${{\mathcal {C}}}_n(\sigma ;{\underline{\theta }})$ in the form (3.16) is ambiguous. It is possible to replace the function ${{\mathcal {N}}}^{(\eta )}(\sigma ;{\underline{\theta }})$ on the right side of (3.16) by other functions ${{\mathcal {N}}}^{(\eta ')}(\sigma ;{\underline{\theta }})$ without changing the functions ${{\mathcal {C}}}_n(\sigma ;{\underline{\theta }})$. In order to see this, let us consider the possibility to replace ${{\mathcal {N}}}^{(\eta )}$ by a function ${{\mathcal {N}}}^{(\eta ')}$ of the form ${{\mathcal {N}}}^{(\eta ')}(\sigma ;{\underline{\theta }})={{\mathcal {E}}}(\sigma ;{\underline{\theta }}) {{\mathcal {N}}}^{(\eta )}(\sigma ;{\underline{\theta }})$. It is easy to see that this replacement will leave the functions ${{\mathcal {C}}}_n(\sigma ;{\underline{\theta }})$ unchanged iff the function

3.19 $$\begin{aligned} {\textsf{e}}\,(\,\sigma \,,{\underline{\theta }}\,):=\frac{{{\mathcal {E}}}(\,\sigma +1\,,{\underline{\theta }}\,)}{{{\mathcal {E}}}(\,\sigma \,,{\underline{\theta }}\,)}, \end{aligned}$$

is periodic in $\sigma$, ${\textsf{e}}(\sigma +1,{\underline{\theta }})={\textsf{e}}(\sigma ,{\underline{\theta }})$. Such a replacement will leave ${{\mathcal {T}}}^{(\eta _0)}$ unchanged, but it will allow us to define another normalisation for the isomondromic tau-function

3.20 $$\begin{aligned} {{\mathcal {T}}}^{(\eta ')}={{\mathcal {E}}}\, {{\mathcal {T}}}^{(\eta )} \end{aligned}$$

admitting an expansion of theta series type,

3.21 $$\begin{aligned}&{{\mathcal {T}}}^{(\eta ')}(\,\sigma \,,\,{\underline{\theta }}\,;\,z\,)=\sum _{n\in {{\mathbb {Z}}}} \,e^{2\pi \textrm{i}n\,\eta '}\, {{\mathcal {N}}}^{(\eta ')}(\sigma +n\,;\,{\underline{\theta }}\,){{\mathcal {F}}} (\,\sigma +n\,,\,{\underline{\theta }}\,;\,z\,) \end{aligned}$$

with $\eta$ related to $\eta '$ such that

3.22 $$\begin{aligned} e^{2\pi \textrm{i}\,\eta }=e^{2\pi \textrm{i}\,\eta '}{\textsf{e}}(\,\sigma \,,{\underline{\theta }}\,). \end{aligned}$$

We thereby see that the function ${{\mathcal {N}}}^{(\eta ')}$ is associated with a new FN-type coordinate $\eta '$ defined using (3.22).

This argument leads to a vast class of possibilities for generalised theta-series expansions of the tau function, since the only requirement we imposed is periodicity of ${\textsf{e}}$, which can be expressed as the following step-two relation for ${{\mathcal {E}}}$

3.23 $$\begin{aligned} {{\mathcal {E}}}(\sigma ) {{\mathcal {E}}}(\sigma +2) = {{\mathcal {E}}}(\sigma +1)^2 \,. \end{aligned}$$

Among this large class of possibilities, however, there turns out to be a rather small distinguished subset.

As an example, let us consider the function

3.24 $$\begin{aligned}&{{\mathcal {N}}}^{(\eta ')}(\,\sigma \,,{\underline{\theta }}\,)= {N(\sigma ,\theta _2,\theta _1)N'(\sigma ,\theta _3,\theta _4)} \end{aligned}$$

3.25 $$\begin{aligned}&N'(\vartheta _1,\vartheta _2,\vartheta _3) = \frac{ \prod _{\epsilon ,\epsilon ' = \pm } G(1+\vartheta _{2}+\epsilon \vartheta _{3}+\epsilon ' \vartheta _{1}) }{(2\pi )^{\vartheta _1}G(1) \prod _{r=1}^{3}G(1+2\vartheta _r) }\,. \end{aligned}$$

This would correspond to the choice

3.26 $$\begin{aligned} {{\mathcal {E}}}(\,\sigma \,,{\underline{\theta }}\,)=S(\theta _3-\sigma +\theta _4)S(\theta _3-\sigma -\theta _4), \qquad S(x):=\frac{G(1+x)}{G(1-x)}. \end{aligned}$$

Noting that *S*(*x*) satisfies the functional relation

3.27 $$\begin{aligned} S(x\pm 1)=\mp (2\sin \pi x)^{\mp 1}S(x), \end{aligned}$$

one easily sees that the function ${\textsf{e}}(\sigma ,{\underline{\theta }})$ associated with the choice (3.26) is periodic in $\sigma$. The coordinate $\eta '$ associated with ${{\mathcal {N}}}^{(\eta ')}$ by means of (3.22) can be identified with an element of the family of coordinates defined in Sect. 3.1 by replacing the normalisation factor ${\textsf{n}}^{(2)}$ defined in Sect. 3.1.4 by ${\textsf{n}}{'} ^{(2)}={\textsf{e}}\,{\textsf{n}}^{(2)}$.

In a similar way one can show that replacing one of the functions *N* in (3.14a) by any one of the functions $N_i$, $i=1,2,3$, defined as

3.28 $$\begin{aligned} N_i(\vartheta _1,\vartheta _2,\vartheta _3) = \frac{ \prod _{\epsilon ,\epsilon ' = \pm } G(1+\vartheta _{i}+\epsilon \vartheta _{i+1}+\epsilon ' \vartheta _{i+2}) }{(2\pi )^{\vartheta _1}G(1) \prod _{r=1}^{3}G(1+2\vartheta _r) }\,,\qquad \vartheta _{i+3}\equiv \vartheta _i, \end{aligned}$$

will leave the functions ${{\mathcal {C}}}_n(\sigma ;{\underline{\theta }})$ unchanged. This means that the functions ${{\mathcal {C}}}_n(\sigma ;{\underline{\theta }})$ have symmetries under permutations of the arguments that are not manifest in the factorisation (3.16).

To conclude this overview of the case of $C_{0,4}$, let us recollect a few of the most relevant facts learned so far. On the one hand, not all choices of $\eta$ induce expansions of ${{\mathcal {T}}}$ having the form of generalised theta-series, $\eta _0$ being a counter-example. On the other hand, different choices of $\eta$ inducing such an expansion involve changes in the normalisation ${{\mathcal {N}}}^{(\eta )}$ of the tau function, compatible with ambiguities in its definition (2.22). In turn, the *relative* normalisation coefficients ${{\mathcal {E}}}$ for ${{\mathcal {T}}}$ are related in a precise way to *relative* normalisation coefficients ${\textsf{e}}$ for $\eta$, as described by (3.19). It is also worth noting that the gluing parameter *z* for $C_1, C_2$ turns out to play the role of the isomonodromy ‘time’ coordinate, as well as the series-expansion parameter in the theta-series coefficients ${{\mathcal {F}}}$. Finally, we should stress that the discussion so far concerns only a *fixed* choice of pants decomposition for $C_{0,4}$, this is directly related to the fact that the theta-series coefficients ${{\mathcal {F}}}$ appearing in (3.13) are the *same* for all these choices of $\eta$.

### Normalised Tau-Functions for $C=C_{0,2}$

A first expansion of theta series form for the tau-function associated with Painlevé III has been found in [61]. This expansion takes the following form

3.29 $$\begin{aligned} {{\mathcal {T}}}^{(w)}(\sigma ',\eta ';\Lambda )= \sum _{n\in {{\mathbb {Z}}}}e^{4\pi \textrm{i}\,n\,\eta '}\, {{\mathcal {N}}}^{(w)}(\sigma '+n) \, {{\mathcal {F}}}(\sigma '+n,\Lambda ) \end{aligned}$$

where

3.30 $$\begin{aligned} {{\mathcal {N}}}^{(w)}(\sigma ) =\prod _{s=\pm }\frac{1}{G(1+2s\sigma )},\qquad {{\mathcal {F}}}(\sigma ,\Lambda )=\Lambda ^{4\sigma ^2}\bigg (1+\sum _{k=1}^\infty {{\mathcal {F}}}_k(\sigma )\Lambda ^{4k}\bigg )\,. \end{aligned}$$

A formula for the coefficients ${{\mathcal {F}}}_k(\sigma )$ can be found in [61]. Comparing with the notations used in [61], one should note that the parameters we denote by $\sigma '$ and $\eta '$ correspond to the parameters denoted $\sigma$ and $\eta$ in [61], respectively. We will see that $\sigma '$ and $\eta '$ are closely related, but not quite identical to coordinates $(\sigma ,\eta )$ of FN type as defined in this paper. It may also be noted that the role of *z* from the case of $C_{0,4}$ has been taken by $\Lambda$, which indeed plays the role of isomonodromy time and expansion parameter for ${{\mathcal {F}}}$.

The functions $(\sigma (\mu ),\eta (\mu ))$ appearing in (3.29) have been defined in [61] by parameterising the Stokes data $\mu$ associated with the irregular singularities at $z=0$ and $z=\infty$ as follows. A pair of solutions $Y^{(0)}(z)$, $Y^{(\infty )}(z)$ of the equation $(\partial _z-A(z))Y(z)=0$ can be defined having asymptotic behaviour at $z\rightarrow 0$ and $z\rightarrow \infty$ of the form

3.31 $$\begin{aligned} \begin{aligned} Y^{(0)}(z)&\simeq G^{(0)}(\sqrt{z}) \big [ 1+{{\mathcal {O}}}(\sqrt{z})\big ]e^{+2\Lambda ^2\frac{1}{\sqrt{z}}\sigma _z}, \\ Y^{(\infty )}(z)&\simeq G^{(\infty )}(\sqrt{z})\big [ 1+{{\mathcal {O}}}(\sqrt{z^{-1}})\big ]e^{-2{\sqrt{z}}\sigma _z}, \end{aligned} \quad \begin{aligned}&-\pi< \arg (z \,\Lambda ^{-4} )< 3\pi ,\\&-\pi< \arg (z)< 3\pi \,, \end{aligned} \end{aligned}$$

where $G^{(0)}(\sqrt{z})$ and $G^{(\infty )}(\sqrt{z})$ are gauge transformations. The Stokes data have been parameterised in [61] in terms of a pair of variables here denoted by $({\sigma }',{\eta '})$ in such a way that the monodromy around $x=0$ gets represented in the following form:

3.32 $$\begin{aligned} \begin{aligned} Y^{(0)}(e^{2\pi \textrm{i}}z)&=Y^{(0)}(z) M_0,\\ Y^{(\infty )}(e^{2\pi \textrm{i}}z)&=Y^{(\infty )}(z) M_\infty \end{aligned} \qquad M_0=\sigma _x M_\infty \sigma _x=\left( \begin{matrix} 0 & \quad \textrm{i} \\ \textrm{i} & \quad -2\cos 2\pi {\sigma '} \end{matrix}\right) , \end{aligned}$$

with $$\sigma _x=\big ({\begin{smallmatrix} 0 & \quad 1 \\ 1 & \quad 0 \end{smallmatrix}}\big )$$, together with the fact that $Y^{(0)}(z)$ and $Y^{(\infty )}(z)$ are related as

3.33 $$\begin{aligned} Y^{(\infty )}(z)=Y^{(0)}(z)E,\quad E=\frac{1}{\sin 2\pi {\sigma }'} \left( \begin{matrix} \sin 2\pi {\eta '} & \quad -\textrm{i}\sin 2\pi ({\eta '}+{\sigma '}) \\ \textrm{i}\sin 2\pi ({\eta '}-{\sigma '}) & \quad \sin 2\pi {\eta '} \end{matrix}\right) . \end{aligned}$$

We will determine the relation between the parameters $\sigma '$ and $\eta '$ and the FN-type variables $(\sigma ,\eta )$ defined in Sects. 3.1.1 and 3.1 in the following subsection.

### Fenchel–Nielsen-Type Coordinates for $C_{0,2}$

We will now see that the coordinates $({\sigma '},{\eta '})$ appearing in the expansion (3.29) are closely related to coordinates of FN type for the Riemann sphere with two irregular singularities of the mildest type. To this aim one may begin by noting that the monodromy in an annulus *A* separating the punctures at $z=0$ and $z=\infty$ is diagonalised by the following change of basis

3.34 $$\begin{aligned} \begin{aligned} Y^{(0)}(z)&=Y_1(z) W_1,\\ Y^{(\infty )}(z)&=Y_2(z) W_2, \end{aligned} \qquad W_1=W_2\sigma _x, \qquad W_2= \left( \begin{matrix} 1 & \quad -\textrm{i}\,e^{-2\pi \textrm{i}{\sigma '}} \\ 1 & \quad - \textrm{i}\,e^{+2\pi \textrm{i}{\sigma '}} \end{matrix}\right) \,, \end{aligned}$$

so that we have for both $i=1,2$,

3.35 $$\begin{aligned} Y_i(e^{2\pi \textrm{i}}z) =Y_i(z) M_A\,, \qquad M_A = - \left( \begin{matrix} e^{2\pi \textrm{i}\sigma '} & \quad 0 \\ 0 & \quad e^{-2\pi \textrm{i}{\sigma '}} \end{matrix}\right) \,. \end{aligned}$$

$Y_1$ and $Y_2$ are related by a diagonal matrix,

3.36 $$\begin{aligned} Y_2(z)=Y_1(z) \, T_A\,, \qquad T_A = WE \sigma _x W^{-1} =-\textrm{i}\, \left( \begin{matrix} e^{-2\pi \textrm{i}{\eta '}} & \quad 0 \\ 0 & \quad e^{2\pi \textrm{i}{\eta '}} \end{matrix}\right) . \end{aligned}$$

This needs to be compared to the definition of the FN-type coordinates used in this paper.

To this aim one may note, on the one hand, that a sphere with two irregular punctures can be represented by gluing two spheres $C_i$ having a regular and an irregular puncture each. Solutions to the Riemann–Hilbert problem on the two-punctured spheres $C_i$ can be obtained by isomonodromic deformations of the solutions $Y_i(z)$ on *C* in the factorisation limit $\Lambda \rightarrow 0$.

In the gluing construction described in Sects. 3.1.1 and 3.1, one needs a pair of functions $(\Phi _1,\Phi _2)$ representing solutions to the Riemann–Hilbert problems on $C_i$. We need to find the pair of functions $(\Phi _1,\Phi _2)$ related to the bases $(Y_1,Y_2)$ defined above by the limit $\Lambda \rightarrow 0$. Let us first consider the solution $Y_2(x)$ of $(\partial _x-A(x))Y_2(x)=0$, with *A*(*x*) given in (2.15). It is related to a solution $\Phi _2(x)$ of $(\partial _x-A_0(x))\Phi _2(x)=0$, with $A_0(x)=A(x)\big |_{\Lambda =0}$, by a gauge transformation, as follows from the equation replacing (2.19) in the case $C=C_{0,2}$.

It next follows from (3.34) that the function $\Phi _2(x)$ is related to a function $\Phi ^{(\infty )}(x)=\Phi _2(x)W_2$ having the same asymptotic behaviour (3.31) for $x\rightarrow \infty$ as $Y^{(\infty )}(x)$. The function $\Phi ^{(\infty )}(x)$ is thereby uniquely defined up to gauge transformations. We claim that

3.37 $$\begin{aligned} \Phi ^{(\infty )}(x)=g^{(\infty )}(x)\left( \begin{matrix} *& \quad {\tilde{*}} \\ K_{2\sigma -1}(2\sqrt{x}) & \quad \textrm{i}K_{2\sigma -1}(2e^{\pi \textrm{i}}\sqrt{x}) \end{matrix}\right) , \end{aligned}$$

with $K_\alpha (x)$ being the modified Bessel function of the second kind, and matrix elements in the upper row left unspecified, being dependent on the choice of gauge transformation $g^{(\infty )}(x)$. In order to check that $\Phi ^{(\infty )}(x)$ represents the limit $\Lambda \rightarrow 0$ of $Y^{(\infty )}(x)$ one may use the formula

3.38 $$\begin{aligned} K_{\alpha }(z)\underset{z\rightarrow \infty }{\sim } \sqrt{\frac{\pi }{2z}}e^{-z}(1+{{\mathcal {O}}}(z^{-1})). \end{aligned}$$

The functions $K_{\alpha }(x)$ are related to the modified Bessel functions $I_\alpha (x)$ of the first kind as

3.39 $$\begin{aligned} K_{\alpha }(z)=\frac{\pi }{\sin \pi \alpha }(I_{-\alpha }(z)-I_{\alpha }(z)). \end{aligned}$$

Noting that the functions $I_{\alpha }(z)$ have diagonal monodromy $I_{\alpha }(e^{2\pi \textrm{i}}z)=e^{2\pi \textrm{i}\alpha }I_{\alpha }(z)$ around $z=0$, it becomes easy to show that $\Phi ^{(\infty )}(x)=\Phi _2(x)W_2$, with

3.40 $$\begin{aligned} \Phi _2(x)=g^{(\infty )}(x)\left( \begin{matrix} *' & \quad *'' \\ I_{2\sigma -1}(2\sqrt{x}) & \quad I_{1-2\sigma }(2\sqrt{x}) \end{matrix}\right) \!,\quad W_2=\frac{\pi }{\sin 2\pi \sigma } \left( \begin{matrix} 1 & \quad -\textrm{i}\,e^{+2\pi \textrm{i}{\sigma }} \\ 1 & \quad - \textrm{i}\,e^{-2\pi \textrm{i}{\sigma }} \end{matrix}\right) \!. \end{aligned}$$

Comparing with (3.34), we conclude that ${\sigma }'=-\sigma$. There is a similar relation between $\Phi _1(x)$ and the limit $\Lambda \rightarrow 0$ of $Y_1(x)$. Comparing (3.36) to the equation (3.1) defining $\eta$ yields the relation $\eta =-2\eta '-\frac{1}{2}$.

### Strong Coupling Expansion

We are now going to observe that the tau-function for Painlevé III admits an expansion of theta series type which does not involve Fenchel–Nielsen-type coordinates as before. The role of the Fenchel–Nielsen type will be taken by the coordinates for the character variety ${{\mathcal {M}}}_{{\textrm{ch}}}(C)$ introduced by Fock and Goncharov.

In addition to the weak coupling expansion reviewed in Sect. 3.3, there is another expansion of the Painlevé III tau function, conjectured in [82]:

3.41 $$\begin{aligned} {\mathcal {T}}^{(s)}(\nu ,\rho ;\Lambda ) = \sum _{n\in {\mathbb {Z}}} e^{4\pi \textrm{i} \rho n}\, {{\mathcal {N}}}^{(s)}(\nu +\textrm{i}n,\Lambda ) \, {{\mathcal {G}}}(\nu +\textrm{i}n,\Lambda ) \end{aligned}$$

where ${{\mathcal {G}}}(\nu ,\Lambda )=1+\sum _{k=1}^\infty {{\mathcal {G}}}_k(\nu )\Lambda ^{-k}$ is a normalised series in powers of $\Lambda ^{-1}$ and the prefactor ${{\mathcal {N}}}^{(s)}(\nu ,\Lambda ) =e^{\frac{\textrm{i}\pi \nu ^2}{4}} 2^{\nu ^2} (2\pi )^{-\frac{\textrm{i}\nu }{2}} G(1+\textrm{i}\nu ) (8\Lambda )^{\frac{\nu ^2}{2}+\frac{1}{4}} e^{4\Lambda ^2 + 8\nu \Lambda }$. Compared to the expansion (3.29) one may note, in particular, that negative powers of $\Lambda$ appear in this expansion.

The parameters $\nu$ and $\rho$ have been defined in [82] as the following functions of the parameters $\sigma ,\eta$ introduced previously,

3.42 $$\begin{aligned} e^{\pi \nu } = \frac{\sin 2\pi \eta '}{\sin 2\pi \sigma '} \,, \qquad e^{4\pi \textrm{i}\rho } = \frac{\sin 2\pi \eta '}{\sin 2\pi (\sigma '+\eta ')} \,. \end{aligned}$$

We will now show that the parameters $\nu$, $\rho$ are particular Fock–Goncharov coordinates.

### Fock–Goncharov Coordinates

**Fig. 1:**
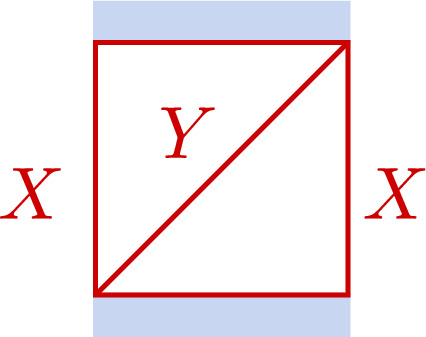
Triangulation of the annulus of the Painlevé III system

Fock–Goncharov coordinates are assigned to triangulations of the Riemann surface [52]. In the case of Painlevé III, we have a triangulation of the annulus with two irregular punctures characterized by a single Stokes sector each (see Sect. 5 for the necessary background regarding Stokes graphs). Such a triangulation *T* therefore has exactly one vertex $v_0$ at $z=0$ and one vertex $v_\infty$ at $z=\infty$. Two edges of *T* correspond to boundary edges: one connects $v_0$ to itself encircling the singularity at $z=0$, the other one has a similar role at $v_\infty$. There are also two internal edges marked by letters *X* and *Y* in Fig. 1. The Fock–Goncharov coordinate $X_e$ associated with an edge *e* separating two triangles of an ideal triangulation is defined as the cross-ratio

3.43 $$\begin{aligned} X_e= -\frac{(s_1\wedge s_2)(s_3\wedge s_4)}{(s_2\wedge s_3)(s_4\wedge s_1)} \,, \end{aligned}$$

where $s_i$ are flat sections satisfying $(\partial _z-A(z))s_i(z)$ vanishing at the puncture representing the *i*-th corner of the quadrilateral formed by the two triangles, with corners labelled counter-clockwise starting from one of the ends of *e*. The cross-ratio (3.43) does not depend on the point where all sections $s_1,\dots ,s_4$ appearing in (3.43) are evaluated.

In order to compute the Fock–Goncharov coordinates in our case, we first need to identify the small flat section at each vertex. This can be inferred from the explicit form of their asymptotic expansions (3.31). For convenience, let $e_1 = (1,0)^t$ and $e_2 = (0,1)^t$, then the flat section is $Y^{(0)}\cdot e_2$.^6^ A similar analysis yields the small flat section at $v_\infty$, it is $Y^{(\infty )}\cdot e_1$.

Computing cross-ratios (3.43) is now straightforward. Let us start from edge *Y*, the sections associated with the four corners of the corresponding quadrilateral are^7^

3.44 $$\begin{aligned} \begin{aligned} s_1&= Y^{(0)} \cdot e_2 \\ s_3&= Y^{(0)}\cdot E\cdot M^{-1}_\infty \cdot e_1 \end{aligned} \qquad \quad \begin{aligned} s_2&= Y^{(0)}\cdot M_0^{-1} \cdot e_2 \\ s_4&= Y^{(0)}\cdot E \cdot e_1 \end{aligned} ~. \end{aligned}$$

Here, we used monodromy data to express $$s_{i}$$ evaluated at $$z_*$$ chosen in the bottom-left corner of Fig. 1, in terms of the local asymptotic solution $$ Y^{(0)}$$, see footnote 6 for details. Thanks to this, all dependence on $$ Y_+^{(0)}(z_*)\wedge Y_-^{(0)}(z_*)$$ drops out of the cross-ratio, leaving

3.45 $$\begin{aligned} Y = -\left( \frac{\sin 2 \pi \sigma '}{\sin 2\pi \eta '}\right) ^2 = -e^{-2\pi \nu }\,. \end{aligned}$$

Similarly, to compute *X* we consider the quadrilateral crossed by the left-most vertical edge in Fig. 1. The four sections are therefore^8^

3.46 $$\begin{aligned} \begin{aligned} s_1&= Y^{(0)} \cdot e_2 \\ s_3&= Y^{(0)}\cdot E \cdot e_1 \end{aligned} \qquad \quad \begin{aligned} s_2&= Y^{(0)}\cdot E\cdot M_\infty ^{-1} \cdot e_1 \\ s_4&= Y^{(0)}\cdot M_0 \cdot e_2 \end{aligned} \end{aligned}$$

plugging these into (3.43) gives the coordinate associated with edge *X*

3.47 $$\begin{aligned} X = -\left( \frac{\sin 2\pi (\sigma '+\eta ')}{\sin 2\pi \sigma '}\right) ^2 = - e^{-8\pi \textrm{i}\rho +2\pi \nu }\,. \end{aligned}$$

We have shown that coordinates $(\nu ,\rho )$ appearing in the strong coupling generalized theta-series expansion of the Painlevé III tau function are particular Fock–Goncharov coordinates.

## Relations Between Theta Series

We have seen that multiplying the isomonodromic tau-functions with monodromy-dependent normalisation factors can define isomonodromic tau functions having expansions of generalised theta series type. These tau functions are essentially uniquely characterised by the set of Darboux coordinates for the space ${{\mathcal {M}}}_{{\textrm{ch}}}(C)$ of monodromy data appearing in the series expansions.

However, there are many such expansions, in general. The relation between any two of them corresponds to a change of Darboux coordinates. In our main examples $C=C_{0,2}$ and $C=C_{0,4}$ we are now going to show that the changes of normalisation relating two tau-functions of the type considered here have an elegant characterisation in terms of the change of Darboux coordinates defining the two partition functions, respectively. They will turn out to be analogs of the generating functions for the relevant changes of Darboux coordinates, defined by replacing derivatives by finite differences in the defining equations.

We had already encountered a first example for difference generating functions in the previous section: Studying different Darboux coordinates associated with a fixed pants decomposition we observed that the discrete derivative (3.19) of the ratio of tau functions (3.20) is directly related to the change of coordinates (3.22). In this section, we will significantly extend this picture, by showing that a similar structure can describe changes of the tau function induced by changes of Darboux coordinates in much larger generality.

### Relation Between Strong and Weak Coupling Expansions

The two expansions of theta series (3.29) and (3.41) quoted above are related by multiplication with a function of the monodromy data,

4.1 $$\begin{aligned} {{\mathcal {T}}}^{(w)}(\sigma ,\eta ;\Lambda )={{\mathcal {X}}}(\sigma ,\nu ){{\mathcal {T}}}^{(s)}(\nu ,\rho ;\Lambda ), \end{aligned}$$

assuming that $(\sigma ,\eta )$ and $(\nu ,\rho )$ are related by (3.42). The main result of [82] is an explicit formula for the function ${{\mathcal {X}}}(\sigma ,\nu )$.

To find this formula, it has been noted in [82] that the theta series expansions for (3.29) and (3.41) imply the difference equations

4.2 $$\begin{aligned} {{\mathcal {T}}}^{(w)}(\sigma +1,\eta ;\Lambda )=e^{-4\pi \textrm{i}\,\eta }\,{{\mathcal {T}}}^{(w)}(\sigma ,\eta ;\Lambda ),\quad {\mathcal {T}}^{(s)}(\nu +\textrm{i},\rho ;\Lambda ) = e^{-4\pi \textrm{i}\,\rho }\,{\mathcal {T}}^{(s)}(\nu ,\rho ;\Lambda ). \end{aligned}$$

Consistency of (4.1) with these difference equations implies that the normalisation factor ${{\mathcal {X}}}(\sigma ,\nu )$ must satisfy the pair of relations

4.3 $$\begin{aligned} {{\mathcal {X}}}(\sigma +1,\nu ) = e^{-4\pi \textrm{i}\eta } {{\mathcal {X}}}(\sigma ,\nu )\,, \qquad {{\mathcal {X}}}(\sigma ,\nu +\textrm{i}) = e^{4\pi \textrm{i}\rho } {{\mathcal {X}}}(\sigma ,\nu )\,. \end{aligned}$$

Both $(\sigma ,\eta )$ and $(\nu ,\rho )$ are Darboux coordinates for the space of monodromy data parameterising solutions of the Painlevé III equation. This follows from our results above relating $(\sigma ,\eta )$ and $(\nu ,\rho )$ to coordinates of FN and FG types, respectively, and has been verified directly in [82]. Equations (4.3) resemble the equations characterising the generating function of the change of Darboux variables from $(\sigma ,\eta )$ to $(\nu ,\rho )$, with derivatives being replaced by finite difference operators. In comparing with the discussion of Sect. 3.2, one may note that (4.3) and (3.19) play analogous roles. Such functions will be called *difference generating functions* in the following.

### Changes of Pants Decomposition

Besides changes of FN-type coordinates associated with different normalisations for $\eta$ in a fixed pants decomposition (as considered in Sect. 3.2), one will have to discuss the changes of pants decomposition. The following summarises some key points needed for such a discussion.

Within the CFT framework, it is straightforward to derive expansions similar to (3.13) corresponding to other pants decompositions of *C*. In the case $C=C_{0,4}$ studied in this section, it suffices to consider the pants decomposition defined by cutting along a curve $\gamma _u$ separating *z*, 1 from 0, $\infty$, in analogy with standard terminology from high energy physics referred to as the *u*-channel. This pants decomposition allows one to define FN-type coordinates $(\sigma _u,\eta _u)$ in the same way as described above. It is then natural to regard the tau-function as a function of the coordinates $(\sigma _u,\eta _u)$. The analog of (3.13) corresponding to the *u*-channel will then have the form

4.4 $$\begin{aligned} {\mathcal {T}}^{(u)}\big (\sigma _u,\eta _u\,;\,{\underline{\theta }}\,;\,z\big )= \sum _{n\in {{\mathbb {Z}}}} \,e^{2\pi \textrm{i}n\eta _u}\, {{\mathcal {G}}}^{(u)}(\,\sigma _u+n\,,\,{\underline{\theta }}\,;\,z\,), \end{aligned}$$

where

4.5 $$\begin{aligned}&{{\mathcal {G}}}^{(u)}(\,\sigma _u\,,\,{\underline{\theta }}\,;\,z\,)={{\mathcal {N}}}^{(u)}(\,\sigma _u\,,\,{\underline{\theta }}'\,) {{\mathcal {F}}}  (\,\sigma _u\,,\,{\underline{\theta }}'\,;\,1-z\,), \end{aligned}$$

with $${\underline{\theta }}'=(\theta _3,\theta _2,\theta _1,\theta _4)$$ if $${\underline{\theta }}= (\theta _1,\theta _2,\theta _3,\theta _4)$$.^9^

A simple but important consequence of the generalised theta-series form of the expansions in (3.13), (4.4) are the difference equations

4.6 $$\begin{aligned} \begin{aligned} {{\mathcal {T}}}^{(s)}\big (\sigma _s+1,\eta _s\,;\,{\underline{\theta }}\,;\,z\big )&= e^{-2\pi \textrm{i}\eta _s}\,{{\mathcal {T}}}^{(s)}\big (\sigma _s,\eta _s\,;\,{\underline{\theta }}\,;\,z\big ),\\ {{\mathcal {T}}}^{(u)}\big (\sigma _u+1,\eta _u\,;\,{\underline{\theta }}\,;\,z\big )&= e^{-2\pi \textrm{i}\eta _u}\,{{\mathcal {T}}}^{(u)}\big (\sigma _u,\eta _u\,;\,{\underline{\theta }}\,;\,z\big ), \end{aligned} \end{aligned}$$

having set ${{\mathcal {T}}}^{(\eta )}\equiv {{\mathcal {T}}}^{(s)}$, $\sigma \equiv \sigma _s$ and $\eta \equiv \eta _s$ to make the notation more uniform. It follows from the Painlevé VI equation that ${{\mathcal {T}}}^{(s)}\big (\sigma _s,\eta _s;\,{\underline{\theta }};\,z\big )$ must be proportional to ${{\mathcal {T}}}^{(u)}\big (\sigma _u,\eta _u;\,{\underline{\theta }};\,z\big )$ if the coordinates $(\sigma _s,\eta _s,{\underline{\theta }})$ and $(\sigma _u,\eta _u,{\underline{\theta }})$ are evaluated on the same point of the character variety,

4.7 $$\begin{aligned} {{\mathcal {T}}}^{(s)}\big (\sigma _s,\eta _s\,;\,{\underline{\theta }}\,;\,z\big )\,=\, {{\mathcal {H}}}(\,\sigma _s,\sigma _u\,,L_t\,;\,{\underline{\theta }}\,)\, {{\mathcal {T}}}^{(u)}\big (\sigma _u,\eta _u\,;\,{\underline{\theta }}\,;\,z\big ). \end{aligned}$$

Choosing $\sigma _s$, $\sigma _u$ and $L_t$ as arguments of the function ${{\mathcal {H}}}$ is of course somewhat redundant. Given $\sigma _s$ and $\sigma _u$ one may use (3.6) together with $L_s=2\cos 2\pi \sigma _s$ and $L_u=2\cos 2\pi \sigma _u$ to determine $L_t$ up to a sign. The factor ${{\mathcal {H}}}(\,\sigma _s,\sigma _u,L_t;\,{\underline{\theta }}_s\,)$ in (4.7) has been calculated explicitly in the remarkable work [79, 81]. The basis for the approach taken in [81] is the functional relations

4.8a $$\begin{aligned} {{\mathcal {H}}}(\sigma _s+1,\sigma _u\,,L_t\,;\,{\underline{\theta }}\,)&= e^{-2\pi \textrm{i}\eta _s(\sigma _s,\sigma _u,L_t)}\,{{\mathcal {H}}}(\sigma _s,\sigma _u\,,L_t\,;\,{\underline{\theta }}\,), \end{aligned}$$

4.8b $$\begin{aligned} {{\mathcal {H}}}(\sigma _s,\sigma _u+1\,,L_t\,;\,{\underline{\theta }}\,)&= e^{+2\pi \textrm{i}\eta _u(\sigma _s,\sigma _u,L_t)}\,{{\mathcal {H}}}(\sigma _s,\sigma _u\,,L_t\,;\,{\underline{\theta }}\,). \end{aligned}$$

The relations (4.8) identify the function ${{\mathcal {H}}}$ as a difference generating function.

One should notice that the changes of normalisation of the tau-functions induced by changes for Darboux coordinates for ${{\mathcal {M}}}_{{\textrm{ch}}}(C)$ can be composed. In this way, one may represent the relations (4.7) involving a rather complicated difference generating function as the composition of similar relations involving the difference generating function for the change from FG to FN-type coordinates considered above.

### Transitions Between Two Sets of Fock–Goncharov Coordinates

Having discussed difference generating functions for transitions between FG and FN coordinates, as well as between FN and FN coordinates associated with different pants decompositions, we now complete the picture with a discussion of transitions between FG and FG coordinates associated with different choices of triangulations.

Among all these cases, the FG–FG transition enjoys a special status. In fact, it should be noted that the transitions considered here and those considered above are not all independent: in particular, the FN–FG transition can be understood as an infinite sequence of FG–FG transitions (as part of the *juggle* phenomenon, see Sect. 5.1 for details). Moreover a change of pants decomposition, i.e. an FN–FN transition, can be usually obtained by an FN–FG transition followed by an FG–FG transition and an FG–FN transition of a different type. In view of this, the FG–FG transition may be regarded as the building block underlying all other cases.

#### Dehn Twist

To illustrate the features of FG–FG difference generating functions, we consider the composition of a flip and a relabelling of coordinates defined by

4.9 $$\begin{aligned} X'=Y^{-1},\qquad Y'=X(1+Y^{-1})^{-2}. \end{aligned}$$

This change of variables can be seen to correspond to a Dehn twist for the triangulation of the annulus shown in Fig. 1. While specific, this case nevertheless exhibits all the features of flips of more general triangulations, it is then transparent how the upcoming analysis extends to the more general setting.

To proceed, we switch to logarithmic variables $x,y,x',y'$,

4.10 $$\begin{aligned} X=e^{2\pi \textrm{i}\,x},\qquad Y=-e^{2\pi \textrm{i}\,y},\qquad X'=-e^{2\pi \textrm{i}\,x'},\qquad Y'=e^{2\pi \textrm{i}\,y'}. \end{aligned}$$

The equations (4.9) can be solved for *Y* and $Y'$,

4.11 $$\begin{aligned} Y(x,x')=-e^{-2\pi \textrm{i}\,{x'}},\qquad Y'(x,x')=e^{2\pi \textrm{i}\,x}(1-e^{2\pi \textrm{i}\,x'})^{-2}. \end{aligned}$$

The difference generating function ${{\mathcal {J}}}(x,x')$ associated with the change of variables (4.9) has the following defining properties,

4.12 $$\begin{aligned} \frac{{{\mathcal {J}}}(x+1,x')}{{{\mathcal {J}}}(x,x')}=-(Y(x,x'))^{-1},\qquad \frac{{{\mathcal {J}}}(x,x'+1)}{{{\mathcal {J}}}(x,x')}=Y'(x,x'). \end{aligned}$$

A function satisfying these properties can be constructed in the following form

4.13 $$\begin{aligned} {{\mathcal {J}}}(x,x')={e^{2\pi \textrm{i}xx'}}{(E(x'))^{2}}, \end{aligned}$$

provided that the function *E*(*z*) satisfies the functional equation

4.14 $$\begin{aligned} E(z+1)=\frac{1}{1-e^{2\pi \textrm{i}\,z}}E(z). \end{aligned}$$

A function *E*(*z*) satisfying (4.14) can be constructed as follows

4.15 $$\begin{aligned} E(z)=(2\pi )^{-z}e^{-\frac{\pi \textrm{i}}{2}z^2}\frac{G(1+z)}{G(1-z)}\,. \end{aligned}$$

An alternative representation of the same function is

4.16 $$\begin{aligned} E(z)=\exp \left( \frac{1}{2\pi \textrm{i}} \textrm{Li}_2(1-e^{2\pi \textrm{i}z}) \right) \,. \end{aligned}$$

### Relations Between Fock–Goncharov and Fenchel–Nielsen Coordinates

In the case $C=C_{0,2}$, we have shown above that the coordinates $(\eta ,\sigma )$ and $(\nu ,\rho )$ introduced in [81] are coordinates of Fenchel–Nielsen and Fock–Goncharov type, respectively. The relation (3.42) thereby acquires an interpretation as a change of coordinates on the character variety. It will be useful to rewrite (3.42) in the following form

4.17 $$\begin{aligned} X = \left( \frac{ V+ V^{-1}}{ U- U^{-1}}\right) ^2, \qquad Y = \left( \frac{ U- U^{-1}}{{UV}+{U}^{-1} {V}^{-1}}\right) ^2, \qquad \begin{aligned}&U = e^{2\pi \textrm{i}\sigma }\,,\\&\textrm{i} V =e^{\pi \textrm{i}(- \eta -2\sigma - 1/2)} \,, \end{aligned} \end{aligned}$$

We see that the change from Fenchel–Nielsen to Fock–Goncharov coordinates becomes rational in these variables. Quite remarkably, we are now going to show that similar relations between these two types of coordinates exist also for more general surfaces *C*.

**Fig. 2:**
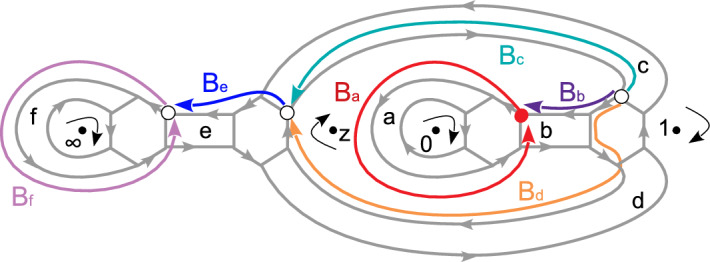
Fat graph on $C_{0,4}$ with six basic paths

In the case $C=C_{0,4}$, one may, for example, consider the triangulation dual to the fat graph depicted in Fig. 2.

One may note that the part of the triangulation dual to this fat graph which is contained in an annulus separating the pairs of punctures (0, *z*) from $(1,\infty )$ is of the same form as considered in the case $C=C_{0,2}$ above. The edges labelled *c* and *d* in Fig. 2 will correspond to the edges labelled with letters *X* and *Y* in Fig. 1. We are going to show that substituting *c* and *d* by the expressions on the right sides of equations (3.42) yields a parameterisation of the monodromy data through FN-type coordinates $\sigma$ and $\eta$.

The Fock–Goncharov coordinates allow us to represent the monodromies on $C_{0,4}$ as compositions of elementary building blocks as follows:

4.18 $$\begin{aligned} \begin{aligned} M_1&=B_a^{-1}~,\\ M_3&= B_b B_d^{-1} B_e^{-1} B_f B_e B_c B_b^{-1} ~, \end{aligned} \qquad \begin{aligned} M_2&= B_b B_c^{-1} B_d B_b^{-1} B_a ~,\\ M_4&= B_b B_d^{-1} B_e^{-1} B_f^{-1} B_e B_d B_b^{-1}~, \end{aligned} \end{aligned}$$

using the following building blocks

4.19 $$\begin{aligned} \begin{aligned}&B_a={\textsf{s}}{{\textsf{v}}}{{\textsf{e}}}(a){{\textsf{v}}}~,~ B_b = {{\textsf{e}}}(b){{\textsf{v}}}~,~ B_c = {{\textsf{e}}}(c) ~,~ \\&B_d = {{\textsf{v}}}{{\textsf{e}}}(d){{\textsf{v}}}{\textsf{s}}{{\textsf{v}}}~,~ B_e = {{\textsf{e}}}(e){{\textsf{v}}}{\textsf{s}}~,~ B_f = {\textsf{s}}{{\textsf{v}}}{{\textsf{e}}}(f){{\textsf{v}}}~, \end{aligned} \end{aligned}$$

with ${\textsf{s}}$, ${{\textsf{v}}}$, ${{\textsf{e}}}$ being the following matrices

4.20 $$\begin{aligned} {\textsf{s}}= \left( \begin{matrix} 0 & \quad 1 \\ -1 & \quad 0\end{matrix} \right) ~, \quad {\textsf{v}}= \left( \begin{matrix} 1 & \quad 0 \\ 1 & \quad 1\end{matrix} \right) ~, \quad {\textsf{e}}(x)= \left( \begin{matrix} 1/\sqrt{x} & \quad 0 \\ 0 & \quad \sqrt{x} \end{matrix} \right) . \end{aligned}$$

The monodromies are defined choosing the point marked in red in Fig. 2 as a base point. The resulting expressions for the trace function $L_u$ are

4.21 $$\begin{aligned} L_u=&-\frac{1}{b d e \sqrt{af}} (a b^2 d^2 e^2 f+a b^2 d^2 e f+a b^2 d^2 e+a b^2 d^2+a b d^2 e^2 f+a b d^2 e f+ a b d^2 e \nonumber \\&+ a b d^2+a b d+b d^2 e^2 f+b d^2 e f+b d^2 e+d^2 e^2 f+d^2 e f \nonumber \\&+d^2 e+b d^2+b d+d^2+d e f+d e+2 d+1) ~. \end{aligned}$$

The eigenvalues $\textrm{m}_i$ of the matrices $M_i$ are for $i=1,2,3,4$ given, respectively, as

4.22 $$\begin{aligned} \textrm{m}_1=\sqrt{a}\,,\quad \textrm{m}_2=-b\sqrt{ad/c}\,,\quad \textrm{m}_3=-e\sqrt{df/c}\,,\quad \textrm{m}_4=\sqrt{f}\,. \end{aligned}$$

It is thereby possible to express the variables *a*, *b*, *e* and *f* as

4.23 $$\begin{aligned} a=\textrm{m}_1^2\,,\quad b=-\frac{\textrm{m}_2}{\textrm{m}_1}\sqrt{\frac{c}{d}}\,,\quad e=-\frac{\textrm{m}_3}{\textrm{m}_4}\sqrt{\frac{c}{d}}\,,\quad f=\textrm{m}_4^2\,. \end{aligned}$$

Using these relations together with $X=d$, $Y=c^{-1}$ one obtains a parameterisation of all monodromy matrices in terms of *X*, *Y*, and $\textrm{m}_1,\dots ,\textrm{m}_4$. By further using (4.17) one obtains a parameterisation of the monodromies on $C_{0,4}$ in terms of the variables $\sigma$ and $\eta$.

In order to compare the coordinates $\sigma$ and $\eta$ defined in this way with the coordinates defined by means of abelianisation in [36] one may note that the resulting expressions for the trace functions $L_{12}= \textrm{Tr} (M_1M_2)$ and $L_{23}= \textrm{Tr}( M_2M_3)$ are found to be of the form

4.24 $$\begin{aligned} \begin{aligned} L_{12}&= 2\cos 2\pi \sigma ' ~,\\ L_{23}&= e^{4\pi \textrm{i}\eta '}C_+(\sigma ')+C_0(\sigma ')+e^{-4\pi \textrm{i}\eta '}C_-(\sigma '), \end{aligned} \end{aligned}$$

with $C_{\pm }(\sigma )$ given as

4.25 $$\begin{aligned} C_s(\sigma ) = 4\,\frac{e^{-4s \pi \textrm{i}\sigma }}{(\sin 2\pi \sigma )^2} \prod _{s'=\pm } {\sin \pi (\sigma +s'\theta _1-s\theta _2) \sin \pi (\sigma +s'\theta _4-s\theta _3)} . \end{aligned}$$

In this way, it becomes easy to check that the coordinates $\sigma '$, $\eta '$ introduced above^10^ as a parameterisation of the Fock–Goncharov coordinates *c* and *d* coincide with one of the systems of coordinates on ${{\mathcal {M}}}_{{\textrm{ch}}}(C_{0,4})$ defined using abelianisation in [36]^11^.

It is clear that a similar relation between FG and FN coordinates will be found for all fat graphs on $C_{0,4}$. In order to see this, it suffices to note that an arbitrary fat graph of $C_{0,4}$ is related to the one in Fig. 2 by a sequence of flips. As the flips are represented in terms of the Fock–Goncharov coordinates by rational changes of coordinates, it suffices to compose changes of coordinates induced by the flips relating a generic fat graph to the one depicted in Fig. 2 with the change of coordinates described above.

We had noted above that there exists a difference generating function for the change of coordinates (4.17). We now see that the *same* change of variables can be used in the case of $C=C_{0,4}$. The difference generating function ${{\mathcal {X}}}(\sigma ,\nu )$ relating normalised tau-functions for Painlevé III can therefore be used to define natural normalised tau-functions for Painlevé VI associated with certain Darboux coordinates of FG type. This observation generalises to even more general changes of coordinates by composing the change between FN-type and FG-type coordinates discussed here with the changes between pairs of FN- and pairs of FG-type coordinates discussed in Sects. 4.2 and 4.3, respectively.

## Coordinates from Exact WKB

In the previous section, we have illustrated how the theta-series expansion of the tau function changes with the choice of Darboux coordinates, both of FG and of FN types. Recall that the coefficients of FN-type theta series expansions, denoted above by ${{\mathcal {F}}}$, can be interpreted as topological string partition functions. This prompts the question of whether certain kinds of expansions can be more natural than others in a given region of the moduli space of quantum curves ${\mathcal {Z}}$. Reformulating this question in terms of Darboux coordinates, this section discusses how exact WKB can be used to associate distinguished coordinates of both FG and FN types to generic pairs $(\Sigma ,\hbar )$, where $\Sigma$ is the classical curve, and $\hbar \in {{\mathbb {C}}}^\times$.

### WKB Networks

The double cover $\Sigma =\{(y,x);y^2+q(x)=0\}$ defined by a quadratic differential *q*(*x*) comes equipped with a canonical differential $\sqrt{q}$. We will assume *q* to have at least one pole of order at least two. A trajectory of $(q,\hbar )$ is a leaf of the foliation defined by the equation

5.1 $$\begin{aligned} \textrm{Im}(w(x))=\mathrm {const.},\qquad w(x)=e^{-\textrm{i}\arg (\hbar )}\int ^x\!\textrm{d}x'\,\sqrt{q(x')}. \end{aligned}$$

A Stokes curve is a trajectory having at least one end at a branch point. The Stokes graph ${{\mathcal {S}}}_{q,\hbar }$ is the graph formed by the Stokes curves of $(q,\hbar )$. ${{\mathcal {S}}}_{q,\hbar }$ has vertices at the branch points of $\Sigma$, and edges represented by trajectories emanating from the branch points.

Saddle trajectories are Stokes curves which either connect two branch points, or closed trajectories which encircle double poles of *q*(*x*). For generic $(q,\hbar )$ one finds Stokes graphs which do not have saddle trajectories. This means that all Stokes curves emanating from a branch point must end at poles of *q*. The complement of ${{\mathcal {S}}}_{q,\hbar }$ is a collection of contractible regions foliated by trajectories connecting poles of *q*. Choosing a generic representative trajectory from each region defines an ideal triangulation of *C* called the WKB triangulation. Generically, regions come in two types, called strips and half-planes. Strips are quadrangles with two double poles of *q* on the boundary, and all trajectories stretch between these poles. Half-planes arise when *q* has a pole of degree higher than two, then trajectories sufficiently close to it have both endpoints on the same pole, but end on it along different (anti-)Stokes rays.

It is useful to study the families of Stokes graphs ${{\mathcal {S}}}_{q,\hbar }$ obtained by varying the phase $\arg (\hbar )$. These families depend on the choice of *q* in an interesting way which has attracted a lot of attention in view of applications to the spectrum of BPS-states in ${{\mathcal {N}}}=2$, $d=4$ supersymmetric field theories of class ${{\mathcal {S}}}$ [64]. For generic values of $\arg (\hbar )$ one finds Stokes graphs defining a WKB triangulation. The topological type of the WKB triangulation will not change for $\arg (\hbar )$ in a wedge of the complex $\hbar$-plane. Any two such wedges are separated by a ray through the origin of the $\hbar$-plane associated with a change of the topological type of Stokes graph.

#### Ring Domains

**Fig. 3:**
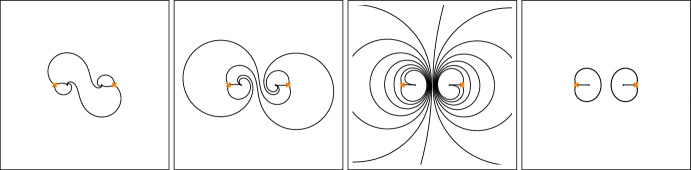
Stokes graphs for the differential (5.2) with $\Lambda =1$ and $U=2$, varying $\arg (\hbar )$ until a non-degenerate ring domain appears

Non-generic cases of particular importance for us are pairs $(q,\hbar )$ where the Stokes graph has non-degenerate ring domains, annular regions embedded in *C* which have empty intersection with ${{\mathcal {S}}}_{q,\hbar }$. Within a ring domain one can use the function *w* defined in (5.1) as a local coordinate. The ring domain is foliated by closed trajectories defined by the equation $\textrm{Im}(w(x))=\textrm{const}$. A ring domain is called degenerate if it consists of closed curves encircling a double pole of *q*. The boundary of a non-degenerate ring domain consists of unions of saddle trajectories connecting a finite, non-empty set of branch points. A collection of Stokes graphs illustrating the transition between Stokes graphs with and without ring domains is depicted in Fig. 3 for quadratic differentials on $C=C_{0,2}$ of the form

5.2 $$\begin{aligned} q(x)=\frac{\Lambda ^2}{z^3}+\frac{U}{z^2}+\frac{\Lambda ^2}{z}. \end{aligned}$$

The existence of a ring domain in the complement of ${{\mathcal {S}}}_{q,\hbar }$ is unstable under small perturbations of *q* or the value $\hbar _*$ of $\hbar$ at which the ring domain appears. In order to understand the nature of this instability one may consider a branch point *b* on the boundary of a ring domain, and the two saddle trajectories emanating from it that bound the ring domain. A small perturbation $(q,\hbar _*e^{\textrm{i}\epsilon })$ of $\hbar _*$ will yield a Stokes graph ${{\mathcal {S}}}_{q,\hbar _*,\epsilon }$ having two Stokes lines emanating from *b* rotated in the same direction relative to the two saddle trajectories of ${{\mathcal {S}}}_{q,\hbar _*}$ emanating from *b* by an angle proportional to $\epsilon$. One of these Stokes lines must traverse the ring domain, the other one will not, see Fig. 3. An arc connecting two branch points on different boundaries of one of the ring domains will intersect any Stokes line traversing the ring domain a number to times which diverges when $\epsilon \rightarrow 0$. This phenomenon is closely related to the phenomenon called “juggle” in [64], featuring two infinite towers of saddles as one takes $\epsilon \rightarrow 0^\pm$. For this reason we will adopt the name “accumulation ray” for the ray $\hbar _*{\mathbb {R}}_{>0} \subset {\mathbb {C}}$ where the ring domain appears.

The neighbourhood of a pair $(q,\hbar )$ having a Stokes graph with ring domains displays certain universal features which play an important role in our story. It will turn out that, roughly speaking, there is a finite neighbourhood of $\epsilon =0$ in which the Stokes graphs ${{\mathcal {S}}}_{q,\hbar _*,\epsilon }$ vary substantially only within ring domain occurring at $\epsilon =0$, and only very little outside. We will in the following describe this behaviour more precisely in the case $C=C_{0,4}$. As a preparation it will be useful to consider the case $C=C_{0,3}$ first.

#### Stokes Graphs on the Pair of Pants

The unique family of quadratic differentials with double poles at punctures on $C_{0,3}$ is:

5.3 $$\begin{aligned} q_0(x) = \frac{- a_3^2 x^2 + (a_3^2 + a_1^2 - a_2^2 ) x - a_1^2}{x^2(x-1)^2}. \end{aligned}$$

**Fig. 4:**
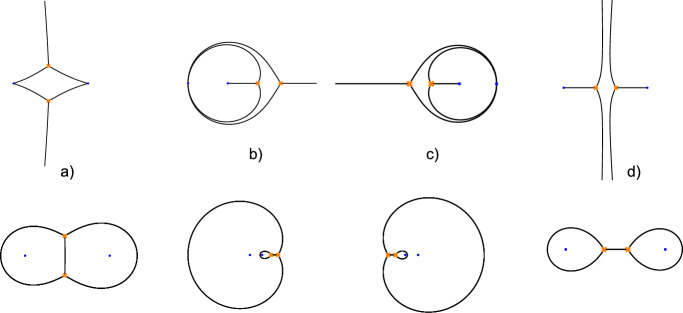
Top row: Stokes graph examples from [13]. Bottom row: Corresponding Fenchel–Nielsen networks, in this case identical with Anti-Stokes graphs. Punctures at finite distance are denoted by a blue dot, an additional puncture is located at infinity and not shown. Turning points are denoted by an orange cross

The classification of the topological types of Stokes graphs is fairly simple in this case [13]: The Stokes graphs can be classified by the number of Stokes lines ending in the punctures. There exist four possibilities, depicted^12^ in Fig. 4. In three of the cases, there are four Stokes lines ending in one puncture and only one Stokes line ending in each of the remaining punctures. In the fourth case, there are two Stokes lines ending in each puncture.

In the cases where four Stokes lines end in a puncture, this puncture is distinguished. It is natural to denote the Stokes graphs having distinguished puncture $z_i$ by ${{\mathcal {S}}}_i$, for $i=1,2,3$, respectively. The remaining one is more symmetric than the other three, suggesting the notation ${{\mathcal {S}}}_s$.

The topological type of Stokes graphs is determined in a simple way by the parameters $\vartheta _i=\textrm{i} a_i/\hbar$, $i=1,2,3$, with $a_i$ corresponding to residues of $\sqrt{q}$ at the three punctures. The type ${{\mathcal {S}}}_i$ is found when

5.4a $$\begin{aligned} \textrm{Re}(\vartheta _i+s\,\vartheta _{i+1}+s'\,\vartheta _{i+2})>0,\qquad \forall \;\,s,s'=\pm 1, \end{aligned}$$

while ${{\mathcal {S}}}_s$ will be realised if

5.4b $$\begin{aligned} \begin{aligned} \textrm{Re}(\vartheta _1+\vartheta _{2}+\vartheta _{3}-2\vartheta _i)&>0, \quad \,i\in \{1,2,3\},\\ \text {and}\quad \textrm{Re}(\vartheta _1+\vartheta _{2}+\vartheta _{3})&>0.\\ \end{aligned} \end{aligned}$$

Saddle trajectories are found at the boundaries of the regions defined by the inequalities defining ${{\mathcal {S}}}_s$ and ${{\mathcal {S}}}_i$, $i=1,2,3$.

*Comment on conventions:* The quadratic differential near a puncture is $q_0 = -\frac{a^2}{x^2} + \dots$. The Stokes lines of $\hbar ^2\partial ^2_x - q_0(x)$ for $\hbar \in {\mathbb {R}}$ correspond to horizontal trajectories emanating from $x = 0$ for $a\in \textrm{i}{\mathbb {R}}$, and to closed trajectories encircling $x = 0$ for $a\in {\mathbb {R}}$. We thus adopt the definition of the parameter $\hbar = \textrm{i}\lambda$, with $\lambda$ being the string coupling in [36].

It will furthermore be useful to keep in mind that parameters like $\vartheta _i$ in [36] coincide with parameters used here. They were naturally real in [36], being related to Kähler parameters $t_i$ as $t_i=R\lambda \vartheta _i$, assuming that *R* and the topological string coupling $\lambda$ are real. The parameters $\vartheta _i$ in [36] were related to the residue parameters $a_{i}$ in the classical curve as $a_{i}=\vartheta _i\lambda$. Real values of the Kähler parameters $t_i$ therefore correspond to real values of the variables $a_i$ used in this paper.

#### Cutting of Stokes Graphs

Let us now investigate the case of a Stokes graph ${{\mathcal {S}}}_{q,\hbar }$ on $C=C_{0,4}$ having a ring domain. Cutting along two distinct closed trajectories foliating the ring domain decomposes *C* into an annular region and two spheres having two punctures and a hole each. One can fill punctured discs into the holes producing two three-punctured spheres $C_1$ and $C_2$ with Stokes graphs defined by ${{\mathcal {S}}}_{q,\hbar }$. The quadratic differentials $q^{(1)}$ and $q^{(2)}$ defined by this procedure on $C_1$ and $C_2$, respectively, have a double pole at the puncture contained in the discs glued into holes with a residue given by the period of $\sqrt{q}$ along the closed trajectories used to decompose *C*. The construction ensures that two out of the four branch points of *q* will be found on each of the two surfaces $C_1$ and $C_2$. The Stokes graphs ${{\mathcal {S}}}_{q,\hbar _*,\epsilon }^{(1)/(2)}$ defined on $C_1$ and $C_2$ using $q^{(1)}$ and $q^{(2)}$ are obtained from ${{\mathcal {S}}}_{q,\hbar }$ by cutting *C* as described above, and deleting all trajectories which do not emerge from the branch points located in $C_1$ and $C_2$, respectively.

We may then discuss using this decomposition of *C* how ${{\mathcal {S}}}_{q,\hbar _*,\epsilon }$ varies with $\epsilon$. A change of the topological type of the Stokes graph occurs exactly when a saddle trajectory occurs. This happens when two Stokes lines emanating from branch points meet each other. The decomposition of *C* into $C_1$, $C_2$ and an annulus allows one to distinguish saddle trajectories located entirely in $C_1$ or $C_2$ from saddle trajectories traversing the annulus. For $\epsilon \ne 0$ one finds a pair of Stokes graphs ${{\mathcal {S}}}_{q,\hbar _*,\epsilon }^{(1)}$ and ${{\mathcal {S}}}_{q,\hbar _*,\epsilon }^{(2)}$ on $C_1$ and $C_2$ which each must have one of the four topological types described in Sect. 5.1.2, respectively.^13^ Importantly, there is a finite range of values for $\epsilon$ in which the topological type of the Stokes graphs ${{\mathcal {S}}}_{q,\hbar _*,\epsilon }^{(1)/(2)}$ does not change under variations of $\epsilon$. This easily follows from the well-known fact that there is a finite number of saddles for ${{\mathcal {S}}}_{q,\hbar _*,\epsilon }^{(1)/(2)}$ as one varies $\epsilon$ for a fixed *q*.^14^ Any other boundary between two regions ${{\mathcal {S}}}_i$ for $i=1,2,3,s$ can only be found at finite non-vanishing values of $\epsilon$. In any neighbourhood of 0 there will, on the other hand, always exist infinitely many values of $\epsilon$ at which there exist saddle trajectories traversing the annulus.

### WKB Expansion

The solutions of the basic ODE $(\hbar ^2\partial _x^2-q_{\hbar } (x))\chi (x)=0$ can be represented as an expansion in $\hbar$, usually referred to as the WKB-expansion. We will here give a very brief summary of the relevant results, referring to [84] for a more detailed discussion and references to the original literature.

The WKB-expansion can conveniently be described by first representing the solution *S* of the Riccati equation $q_\hbar =\hbar ^2({S}^2+{S}')$ as a formal series

5.5 $$\begin{aligned} {S}(x)\equiv {S}(x;\hbar )=\sum _{k=-1}^{\infty } \hbar ^k{S}_k(x). \end{aligned}$$

The family of functions $\{{S}_k(x);k\ge -1\}$ must satisfy the recursion relations

5.6 $$\begin{aligned} ({S}_{-1}(x))^2=q^{(0)}(x)~, \qquad 2{S}_{-1}{S}_{n+1}+{S}_n' +\sum _{\begin{array}{c} k+l=n\\ 0\le k,l\le n \end{array}} {S}_k{S}_l= q^{(n+2)}\quad \text {for}\;\,n\ge -1, \end{aligned}$$

where $q^{(n)}(x)$ is defined by $q_\hbar (x)=\sum _{n=0}^{\infty }\hbar ^nq^{(n)}(x)$. The series (5.5) is usually not convergent, but turns out to be Borel summable under certain conditions,^15^ see [84] for a careful discussion of the relevant results. Let ${S}^{(\pm )}$ be the solutions to the Riccati equation with leading terms $\pm \sqrt{q_0}$, and let ${S}_{\mathrm{\scriptscriptstyle odd}}=\frac{1}{2}({S}^{(+)}-{S}^{(-)})$.

One natural pair $\chi ^{(b)}_{\pm }$ of solutions to $(\hbar ^2\partial _x^2-q_{\hbar } (x))\chi (x)=0$ is normalised at a branch point *b* of $\Sigma$. The solutions can be represented in the form

5.7 $$\begin{aligned} \chi _\pm ^{(b)}(x)=\frac{1}{\sqrt{{S}_\mathrm{\scriptscriptstyle odd}(x)}}\exp \bigg [\pm \int _{{{\mathcal {C}}}_{b,x}}\textrm{d}x'\;{S}_\mathrm{\scriptscriptstyle odd}(x')\bigg ]. \end{aligned}$$

The contour ${{\mathcal {C}}}_{b,x}$ can be chosen to be one-half of a path $\beta _{b,x}$ on $\Sigma$ starting at the pre-image of *x* on the second sheet, encircling the branch point *b*, and ending at the pre-image of *x* on the first sheet. Such solutions clearly depend on the choice of the branch point *b* and satisfy a simple normalisation condition at this point.

Another pair $\chi _{\pm }^{(p)}$ of solutions is normalised at a double pole *p* of the quadratic differential *q*. Assuming that *x* is a local coordinate such that $x(p)=0$, we may define $\chi _{\pm }^{(p)}$ as

5.8 $$\begin{aligned} \chi _{\pm }^{(p)}(x)=\frac{x^{\pm (\sigma -\frac{1}{2})}}{\sqrt{{S}_\mathrm{\scriptscriptstyle odd}}}\exp \left( \pm \int _0^x \textrm{d}x' \left( {S}_\mathrm{\scriptscriptstyle odd}-\frac{2\sigma -1}{2x'}\right) \!\right) , \quad \sigma =\frac{1}{2}+\mathop \textrm{Res}_{x=0}({S}_{\mathrm{\scriptscriptstyle odd}}). \end{aligned}$$

Note that on $C_{0,3}$ this implies the following relation $\sigma = \frac{1}{2} + \frac{a_i}{\hbar }$, if the puncture *p* is identified with the puncture with residue $a_i$.

Another important object defined using the WKB expansion is the Voros-symbol $e^{V_{\gamma }}$ associated with the cycle $\gamma$ on $\Sigma \setminus {\hat{P}}$, with ${\hat{P}}$ being the pre-image of the set of poles and zeros of *q* under the covering projection $\Sigma \rightarrow C$. The formal series $V_\gamma$ is defined as [39, 84]

5.9 $$\begin{aligned} V_{\gamma }=\int _{\gamma }\textrm{d}x'\;{S}_{\mathrm{\scriptscriptstyle odd}}(x'). \end{aligned}$$

The definition of the Voros symbols can be generalised to paths starting and ending at poles of *q*. Assuming that *p* is a double pole of *q* as considered above, and *b* is a branch point lying in the same chart with coordinate *x* one can define [14, 84]

5.10 $$\begin{aligned} V^{(pb)}=V^{(pb)}_{>0}+V^{(pb)}_{\le 0},\qquad \begin{aligned}&V^{(pb)}_{\le 0}=\frac{1}{2}\lim _{x\rightarrow 0}\left( \int _{\gamma _x}\textrm{d}x'\;{S}^{^{\scriptscriptstyle \mathrm odd}}_{\le 0}(x')+(2\sigma -1)\log (x)\right) ,\\&V^{(pb)}_{> 0}=\frac{1}{2}\int _{\gamma _0}\textrm{d}x'\;{S}^{^{\scriptscriptstyle \mathrm odd}}_{>0}(x'), \end{aligned} \end{aligned}$$

using the notations ${S}^{^{\scriptscriptstyle \mathrm odd}}_{>0}=\sum _{k>0}\hbar ^k{S}^{^{\scriptscriptstyle \mathrm odd}}_{k}$, ${S}^{^{\scriptscriptstyle \mathrm odd}}_{\le 0}=\sum _{k\le 0}\hbar ^k{S}^{^{\scriptscriptstyle \mathrm odd}}_{k}$, and $\gamma _x$ being a contour starting at *x*, encircling *b* before returning to *x*.

Under certain conditions, it turns out that the series for $\chi _{\pm }^{(b)}$, and ${V}_\gamma$ are Borel summable. In particular, this holds if the foliation has at most one saddle connection, ensuring that each domain is either a strip, a half-plane, or a degenerate ring domain. We refer to [84] for a review of the known results on Borel summability.

### Fock–Goncharov Coordinates from Exact WKB

If the formal series defining the Voros symbols $V_{\gamma }$ are Borel summable, one may use the functions defined thereby as local coordinate functions for the space ${{\mathcal {Z}}}$ of quantum curves. Whenever the Stokes graph associated with $(q,\hbar )$ is saddle-free, one may naturally assign cycles $\gamma$ defining $V_{\gamma }$ to edges of the WKB triangulation associated with $(q,\hbar )$ as follows. An edge of the WKB triangulation may either be a boundary edge corresponding to a generic leaf trajectory near a higher-order pole of *q*, or it may separate two triangles containing different branch points of *q*, or it may be an edge in the interior of a self-folded triangle obtained by deforming an ordinary triangle until two corners and two sides lie on top of each other, being surrounded by the third side. We will not assign cycles to boundary edges. In the case of internal edges, one may define $\gamma$ as the homology class of the anti-invariant lift of a segment dual to the edge, connecting the pair of branch points on either side. The orientation may be fixed by demanding $\textrm{Re}\hbar ^{-1} \oint _\gamma \sqrt{q_0} >0$. The definition of the path for the case of a self-folded triangle can be found in [84, Figure 11].

The Borel sum of the Voros symbol then defines coordinate functions on ${{\mathcal {Z}}}$. It has been shown in [5] that these coordinate functions coincide with the coordinates obtained from the FG coordinates associated with the WKB triangulation defined by $(q,\hbar )$ by composition with the holonomy map from ${{\mathcal {Z}}}$ to the character variety. It seems natural to conjecture that the relation between Voros symbols and Fock–Goncharov extends from ${{\mathcal {M}}}_{\textrm{op}}^\hbar (C)$ to ${{\mathcal {M}}}_{\textrm{conn}}^\hbar (C)$.

The relation between Fock–Goncharov coordinates and Voros symbols proven in [5] offers a direct explanation of the previous results from [39, 84] showing that the transformations of the Voros symbols associated with a flip of WKB triangulation take the form of cluster mutations familiar from the theory of FG coordinates. This implies that the transformations of the FG coordinates induced by a change of Stokes graph dual to a flip of the triangulation represent examples of the Stokes phenomenon studied in [24]. The FG coordinates associated with a particular type of Stokes graph admit analytic continuations beyond the region of ${{\mathcal {Z}}}$ characterised by the given Stokes graph. The cluster transformations give the expressions for the analytic continuation of the set of coordinates associated with one type of WKB triangulation in terms of the coordinates associated with a WKB triangulation related to the first one by a flip.

### Fenchel–Nielsen-Type Coordinates from Exact WKB

Exact WKB can be used in a rather different way to define coordinates of Fenchel–Nielsen type. A pair $(q,\hbar )$ having an associated Stokes graph with a maximal number of non-degenerate ring domains defines a pants decomposition of *C*.^16^ When *C* is a sphere with four regular punctures, this amounts to having one ring domain. We may first recall from Sect. 3.1 that the FN-type coordinates associated with a given pants decomposition are uniquely defined by fixing the relative normalisation of the solutions $\Phi _i(x_i)$ on the two pairs of pants $C_i$, $i=1,2$, arising in such a decomposition.

It remains to be noticed that the exact WKB method offers natural ways to fix the normalisation of each of the solutions $\Phi _i$, $i=1,2$, by constructing them as Borel sums $\Psi _i$ of formal WKB series. We choose a pair $(q,\hbar _*)$ whose Stokes graph features a non-degenerate ring domain *A*, and assume for simplicity that the corresponding non-contractible cycle on *C* is separating. We then choose $\hbar \ne \hbar _*$ and generic, meaning that $(q,\hbar )$ is saddle-free on *C*, within a sufficiently small wedge centred around $\hbar _*$. Picking branch points $b_i$, $i=1,2$ on distinct components of the boundary of *A*, we may define

5.11 $$\begin{aligned} \Psi _i(x) =\left( \begin{matrix} \partial _x\psi ^{(b_i)}_{+}(x) & \quad \partial _x{\psi }^{(b_i)}_-(x) \\ \psi ^{(b_i)}_+(x) & \quad {\psi }^{(b_i)}_-(x) \end{matrix}\right) , \end{aligned}$$

where $\psi ^{(b_i)}_\pm (x)$, $i=1,2$, are defined on the annulus *A*, respectively, in neighbourhoods of $b_i$, as follows:

- $\psi ^{(b_i)}_+(x)$, $i=1,2$ are the solutions to $(\hbar ^2\partial _x^2-q_{\hbar } (x))\psi (x)=0$ defined by Borel summation of the WKB series (5.7) with the following behaviour along a Stokes line $x(\tau )$ traversing the annulus,^17^
  5.12 $$\begin{aligned} \psi ^{(b_i)}_+(x)=\left\{ \begin{aligned} \chi _+^{(b_i)}(x) \quad \text {if}\quad \frac{d}{d\tau }\left( e^{-\textrm{i}\,\arg {\hbar }}\int _{b_i}^x\sqrt{q_0}(x) \right) <0,\\ \chi _-^{(b_i)}(x) \quad \text {if}\quad \frac{\textrm{d}}{\textrm{d}\tau }\left( e^{-\textrm{i}\,\arg {\hbar }}\int _{b_i}^x\sqrt{q_0}(x) \right) >0, \end{aligned}\right. \end{aligned}$$
- $\psi _{-}^{(b_i)}(x)$, $i=1,2$, are the unique solutions to $(\hbar ^2\partial _x^2-q_{\hbar } (x))\psi (x)=0$ with diagonal monodromy around *A* satisfying the condition
  5.13 $$\begin{aligned} \textrm{det}(\Psi _i(x))=\psi _{-}^{(b_i)}(x)\partial _x\psi _{+}^{(b_i)}(x)-\psi _{+}^{(b_i)}(x)\partial _x\psi _{-}^{(b_i)}(x)=1. \end{aligned}$$
  The functions $\psi _{-}^{(b_i)}(x)$ can be expressed in terms of $\psi _{+}^{(b_i)}(x)$ by the formula
  5.14 $$\begin{aligned} \psi _{-}^{(b_i)}(x)=\frac{1}{1-e^{2\pi \textrm{i}\,\sigma }}\,\psi _{+}^{(b_i)}(x)\int _{\gamma _x}dx' \,{(\psi _{+}^{(b_i)}(x'))^{-2}},\qquad i=1,2, \end{aligned}$$
  where $\sigma$ is defined by representing the monodromy of $\psi _+^{(b_i)}(x)$ around *A* as $(\gamma _x\psi _+^{(b_i)})(x)=e^{-2\pi \textrm{i}\,\sigma }\psi _+^{(b_i)}(x)$, and $\gamma _x$ is a closed contour starting at *x* encircling *A* exactly once.

This definition is “as canonical as possible” in the sense that it only depends on the choices of branch points $b_i$, $i=1,2$, whenever boundaries of *A* have more than one branch point on either component.^18^ The definition of $\psi _+^{(b_i)}(x)$ invokes Borel resummation for the wavefunctions, which is defined piecewise in domains bounded by Stokes lines. However the choice of a recessive WKB solution, which is preserved by Stokes jump across the corresponding Stokes line, is well defined in a neighbourhood of $b_i$ that extends across the Stokes line. This is a direct consequence of the connection formula derived in [114, Section 6]. The definition of $\psi _+^{(b_i)}(x)$ is then extended to a universal covering of the annulus *A* by analytic continuation away from this neighbourhood. On the other hand, the definition of $\psi _-^{(b_i)}$ does not invoke Borel resummation at all, instead it relies entirely on the definition of $\psi _+^{(b_i)}$. Thus, both these definitions are well-posed, for generic $\hbar$.

### Voros Symbols as Normalisation Factors

In the above, we have been considering surfaces *C* which can be represented by gluing two surfaces $C_i$ having punctures $P_i$ with coordinate neighbourhoods $D_i$ surrounding $P_i$. This has been done by identifying points in annuli $A_i=D_i{\setminus } D'_i$, $D'_i$ being smaller discs containing $P_i$. The construction described in the previous subsection defines functions $\Psi _i(x)$ in the annulus $A\subset C$ constructed by identifying $A_1\subset C_1$ and $A_2\subset C_2$.

Conversely, let *C* be a Riemann surface, and let $q_0$ be a holomorphic quadratic differential on *C* such that there exists $\hbar \in {{\mathbb {C}}}^*$ such that $\hbar ^{-2}q_0$ has a ring domain *A*. The function $w(x)=\int ^x \sqrt{q}$ defines a coordinate in *A* making $q_0$ constant, $(dw)^2=q(x)(dx)^2$. If $a=\frac{1}{2\pi \textrm{i}}\int _\gamma \sqrt{q_0}$ is the period around *A*, one can define a function $y(x)=\exp \big (\pm \frac{1}{a}\int ^x_b \sqrt{q}\big )$ such that $\sqrt{q_0}=\pm a\frac{dy}{y}$. If *b* is a point on the boundary of *A* one may choose the sign in the definition of *y*(*x*) such that $|y(x)|>1$ for $x\in A$, giving a function *y*(*x*) from *A* to the annulus $A_h=\{y\in {{\mathbb {C}}};1<|y|<h\}$.

By cutting *C* along the two boundaries of *A*, one can define two surfaces ${\check{C}}_{i}$, $i=1,2$, each having a hole and containing annuli $A_i$ mapping to *A* under the canonical embedding $\check{C}_i\hookrightarrow C$. The coordinate *y* canonically defines coordinates $y_i$ on the annuli $A_i$ for $i=1,2$. Gluing punctured discs to the inner or outer boundaries of $A_i$ defines, for $i=1,2$, respectively, punctured surfaces $C_i$ with quadratic differentials $q_i$ having punctures $p_i$ contained in discs $D_i$ equipped with coordinates $y_i$ vanishing at $p_i$ in which the quadratic differentials $q_i$ are represented simply as $q_i=\frac{a^2}{y^2}(dy_i)^2$.

In this set-up, one has a natural one-to-one correspondence between flat sections $\Psi$ on *C* and pairs of flat sections $\Psi _i$ on $C_i$. The Borel summation of the WKB expansion can be used to define two types of flat sections in $C_i$, $i=1,2$. The flat section $\Psi _i$, is of the form (5.11) with $\psi _\pm ^{(b_i)}$ normalised at the branch points $b_i$ at the boundary of $D_i$. Another flat section denoted $\Psi _i^{(0)}$ is defined by replacing in (5.11) $\psi _\pm ^{(b_i)}$ by $\psi _{\pm }^{(p_i)}$, normalised at the punctures $p_i$, for $i=1,2$, respectively. Note that the functions $\psi _{\pm }^{(p_i)}(y)$ behave near $y=0$ as

5.15 $$\begin{aligned} \psi ^{(p_i)}_\epsilon (y)=\frac{y^{\epsilon (\sigma -\frac{1}{2})}}{\sqrt{{2\sigma -1}}} (1+{{\mathcal {O}}}(y)), \end{aligned}$$

corresponding to having $\nu _+^{(i)} = \nu _-^{(i)}$ in (3.2), whereas the behaviour of the functions $\psi ^{(b_i)}_\epsilon (x)$ is of the form

5.16 $$\begin{aligned} \psi ^{(b_i)}_\epsilon (y)=\nu ^{(b_i)}_\epsilon \frac{ y^{\epsilon (\sigma -\frac{1}{2})}}{\sqrt{{2\sigma -1}}}(1+{{\mathcal {O}}}(y)). \end{aligned}$$

The condition (5.13) implies the relation $\nu ^{(b_i)}_+\nu ^{(b_i)}_-=1$. It follows that the solutions $\psi ^{(b_i)}_\epsilon (x)$ are uniquely characterised by the normalisation factors $\nu ^{(b_i)}_+$. It is not hard to show that $\nu ^{(b_i)}_+$ are given by the Voros symbols^19^

5.17 $$\begin{aligned} \nu ^{(b_1)}_+=e^{V^{(p_1b_1)}}\,, \qquad \nu ^{(b_2)}_+=e^{-V^{(p_2b_2)}}\,. \end{aligned}$$

As explained in Sect. 3.1, each of the flat sections $\Psi _i$ and $\Psi ^{(0)}_i$ leads to an unambiguous definition of twist coordinates $\eta$ and $\eta _0$ of FN type, respectively. Useful quantities characterising the relation between $\eta$ and $\eta _0$ are the normalisation factors

5.18 $$\begin{aligned} {\textsf{n}}^{(1)} :=\frac{\nu ^{(1)}_-}{\nu ^{(1)}_+}=\big (\nu ^{(1)}_+\big )^{-2}=e^{-2V^{(p_ib_i)}} \,, \qquad {\textsf{n}}^{(2)} :=\frac{\nu ^{(2)}_+}{\nu ^{(2)}_-}=\big (\nu ^{(2)}_+\big )^2=e^{-2V^{(p_ib_i)}} \,. \end{aligned}$$

The normalisation factors ${\textsf{n}}^{(i)}$ allow us to represent the relation between $\eta$ and $\eta _0$ in the form

5.19 $$\begin{aligned} e^{2\pi \textrm{i}\,\eta }\,{\textsf{n}}^{(1)}{\textsf{n}}^{(2)}=e^{2\pi \textrm{i}\eta _0}. \end{aligned}$$

Indeed, $\Psi _i$ and $\Psi ^{(0)}_i$ are related by right multiplication with the diagonal matrix $\textrm{diag}(\nu ^{(b_i)}_+,\nu ^{(b_i)}_-)$. It follows that the matrices representing the monodromies of $\Psi _i$ and $\Psi ^{(0)}_i$ have off-diagonal elements differing by factors of $({\textsf{n}}^{(i)})^{\pm 1}$.

### FN-Type Coordinates as Limits of FG-Coordinates

We have now seen how Exact WKB can be employed in different situations to produce coordinates of two different types. On the one hand, FG coordinates can be defined for generic choices of $(q,\hbar )$. On the other hand, the definition of FN-type coordinates relies on certain properties of the Stokes graph that only arise in particular circumstances. The relevant class of Stokes graphs for which one may define FN-type coordinates corresponds to those choices of *q* for which, upon specialization of $\hbar$ to a distinguished value $\hbar _*$, the Stokes graph develops a non-degenerate ring domain (cf. Sect. 5.1). We henceforth refer to the regions in the moduli space of quadratic differentials characterized by this property as ‘weak coupling’ regimes.^20^

Weak coupling regions have the special property that they admit simultaneously the definition of *both* FG- and FN-type coordinates. In these situations it turns out that FN-type coordinates are often preferable over FG coordinates, for the purpose of expanding the tau function as a theta series. It should be noted that not all quadratic differentials belong to weak coupling regions, in fact in some cases it is clear that there are no such differentials at all.^21^ For this reason, FG coordinates are often necessary to cover other regions of the moduli space. It may also occur that multiple ring domains appear for a given *q* for different values of $\hbar$, corresponding to cycles inducing different factorizations of *C*. Clearly, such situations allow to transition between FN-type coordinates associated with different factorizations.

Overall, our goal is to determine a set of distinguished coordinates associated with each region of the moduli space, with the property that they induce theta series expansions of the tau function. Taken together, FG- and FN-type coordinates cover the whole moduli space, and each is canonically assigned to a different region. Weak coupling regions are those where the two domains of definition overlap. We will now describe how the two sets of coordinates patch together.

*5.6.1 Coordinate transformation*

In the neighbourhood of an accumulation ray, as described in Sect. 5.1, one finds an infinite sequence of wedges in the $\hbar$-plane having associated networks allowing us to define simultaneously coordinates of FG and FN types. It turns out that two types of coordinates defined in this way have a simple relation. Remarkably one finds the same relation both in the case $C=C_{0,2}$ (Painlevé III) and $C=C_{0,4}$ (Painlevé VI), which can be represented as follows:

5.20 $$\begin{aligned} Y(U,V)=\left( \frac{U-U^{-1}}{V+V^{-1}}\right) ^2, \qquad X(U,V)=\left( \frac{VU+(UV)^{-1}}{U-U^{-1}}\right) ^2, \end{aligned}$$

using the notations $U=e^{2\pi \textrm{i}\,\sigma '}$ and $V=\textrm{i}\,e^{2\pi \textrm{i}\,\eta '}$. In both cases, one can identify *X* and *Y* with (part of) the FG-type coordinates associated with the WKB networks appearing in this context.

**Fig. 5:**
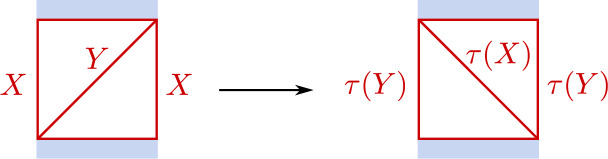
The flip of a triangulation on the annulus

To sharpen the statement of relation (5.20), one must specify a choice of chart both for FG coordinates and for FN-type coordinates. FG coordinates are associated with edges of an ideal triangulation, which in our case is the WKB triangulation defined by $(q,\hbar )$ as explained above. To fix a triangulation and determine which edges correspond to *X*, *Y*, we start by fixing *q* so that there is an accumulation ray containing $\hbar _*$. Varying the phase of $\hbar$ away from $\hbar _*$ induces infinite sequences of flips in either direction. Under good conditions, the infinite sequence of rays lies within a wedge of finite angular width ‘centred’ at the accumulation ray.^22^ Choosing $\hbar$ anywhere within this wedge, such that the Stokes graph is saddle-fee, gives a triangulation with two edges traversing the annulus, as in Fig. 5. Then, *X* and *Y* are the FG coordinates associated with these edges, for more details we refer to [64]. FN-type coordinates, on the other hand, are defined by a choice of non-contractible cycle on *C* induced by the ring domain appearing at $\hbar _*$.^23^

As emphasized, there are infinitely many wedges in the $\hbar$-plane near the accumulation ray of $\hbar _*$. We have not specified any particular choice of wedge, except for the requirement that one must stay “sufficiently close” to the accumulation ray. Switching from a wedge to another involves crossing a finite number of active rays^24^, which induces a change in the topology of the Stokes graph, which in turn changes *both* FG coordinates $(X,Y)\rightarrow (X',Y')$ and FN-type coordinates $(U,V)\rightarrow (U',V')$. The former jump can be understood via the duality between a Stokes graph and a WKB triangulation (which undergoes a flip). Likewise, the change in FN-type coordinates can be understood through their definition based on curve factorization and Exact WKB on each pair of pants. We will show below that the two jumps are *compatible* with relation (5.20), in the sense that applying this formula to the FN-type coordinates $(U',V')$ gives precisely the FG coordinates $(X',Y')$. For this reason, the validity of (5.20) holds separately in each sector within a sufficiently narrow wedge across an accumulation ray. In the remainder of this subsection, we give a simple derivation of this property.

*5.6.2 Dehn twist*

We are now going to describe the family of WKB triangulations occurring in sufficiently small neighbourhoods of an accumulation ray. (A definition of “sufficiently small” will be given below.)

It is easy to see that crossing an active ray will lead to a change of topological types of the Stokes graphs which can be represented by the diffeomorphisms from *A* to itself representing the effect of a Dehn twist. The induced changes of coordinates describe a discrete evolution that we will now describe in more detail.

We fix a triangulation *T* of standard form in an annulus *A* associated with a non-contractible curve.^25^ We may represent *A* as the quotient $\{x\in {{\mathbb {C}}};|\textrm{Im}(x)|\le 1\}/{{\mathbb {Z}}}$, with $1\in {{\mathbb {Z}}}$ acting as $x\rightarrow x+1$. A triangulation of *A* is defined by the left side $e=i[0,1]$ and the diagonal $d=\{x\in {{\mathbb {C}}},0\le \textrm{Im}(x)=\textrm{Re}(x)\le 1\}$. We can assign FG-coordinates *X* and *Y* to the edges *e* and *d*, respectively. A flip changes the triangulation above to another triangulation defined by $e'=e$ and $d'=\{x\in {{\mathbb {C}}},0\le \textrm{Im}(x)=1-\textrm{Re}(x)\le 1\}$. The flip induces a change of coordinates

5.21 $$\begin{aligned} X'=X (1+Y^{-1})^{-2}, \qquad Y'=\frac{1}{Y}. \end{aligned}$$

A diffeomorphism representing a Dehn twist is equivalent to a flip changing the diagonal to the opposite one, followed by a relabelling of the edges $X'\rightarrow Y'$, $Y'\rightarrow X'$. The automorphism of the Poisson-algebra *A* induced by the Dehn twist will be denoted $\tau$. It can be represented as

5.22 $$\begin{aligned} \tau (X)=\frac{1}{Y}, \qquad \tau (Y)=X(1+Y^{-1})^{-2}. \end{aligned}$$

One may note, on the other hand, that the Dehn twist induces the simple change of FN-type coordinates

5.23 $$\begin{aligned} U'\equiv {\tilde{\tau }}(U)=U\,, \qquad V'\equiv {\tilde{\tau }}(V)=VU^{-1}\,. \end{aligned}$$

To see this, recall the definition of *V* in terms of $\eta$ given below (5.20), together with the relation between $\eta$ and Voros symbols in (5.18)–(5.19). As the Voros symbols are defined by a choice of integration contour starting from a branch point $b\in \partial A$ and progressing into the annulus *A*, the effect of a Dehn twist is to modify this contour by adding a detour along the non-contractible cycle associated with *A*. As a result, the Voros symbol shifts precisely by $\sigma$, since $e^{\pm 2\pi \textrm{i} \sigma }$ are the eigenvalues of the transport matrix $M_A$ around *A* (cf. Sect. 3.1). Recalling the definition of *U* in terms of $\sigma$ given below (5.20), explains (5.23).

Having established how each set of coordinates transforms under Dehn twists corresponding to phase-rotations of $\hbar$ near an accumulation ray, it remains to show that the change of coordinates (5.20) maps the discrete evolution ${\tilde{\tau }}$ to $\tau$. This is a simple exercise which we leave to the interested reader. We will henceforth rename ${\tilde{\tau }}$ into $\tau$.

We can now give a definition for what it means to take a sufficiently small wedge around an accumulation ray. We define this to be a wedge in $\hbar$-plane corresponding to a family of WKB triangulations related to each other by a sequence of Dehn twists, which affects only the edges crossing the annulus, in the way shown in Fig. 5. This is the region in which one may simultaneously define FG- and FN-type coordinates associated with the non-contractible curve of the ring domain at $\hbar _*$.

*5.6.3 Limiting behaviour*

Next we consider the limiting behaviour of both types of coordinates induced by the Dehn twist dynamics generated by iterating the changes of coordinates associated with the active rays in the vicinity of the accumulation ray.

In particular, we will be interested in comparing FN-type coordinates (*U*, *V*) chosen in a generic, but fixed, angular sector with the limiting values of FG coordinates (*X*, *Y*). As we will see, there is a well-defined limit for this relation, which moreover turns out simpler than the change of variables (5.20) relating FG and FN-type coordinates associated with the same sector.

Assuming that $|U|<1$, we observe that

5.24a $$\begin{aligned} \tau ^n(Y)&=\left( \frac{U-U^{-1}}{VU^{-n}+V^{-1}U^{n}}\right) ^2\sim U^{2n}(U-U^{-1})^2 V^{-2} ~, \end{aligned}$$

5.24b $$\begin{aligned} \tau ^n(X)&=\left( \frac{VU^{1-n}+V^{-1}U^{n-1}}{U-U^{-1}}\right) ^2\sim U^{-2n}\frac{(UV)^{2}}{(U-U^{-1})^2} ~. \end{aligned}$$

It easily follows that

5.25a $$\begin{aligned} U^2&=\lim _{n\rightarrow \infty } \tau ^n(XY)~, \end{aligned}$$

5.25b $$\begin{aligned} V^2&=(U-U^{-1})^2\lim _{n\rightarrow \infty }U^{2n-1}\tau ^n\big (\sqrt{X/Y}\big ) ~. \end{aligned}$$

Equations (5.24), (5.25) clarify the relation between the FN-type coordinates (*U*, *V*) defined in an angular sector in the $\hbar$-plane chosen in the vicinity of an accumulation ray, and limits $n\rightarrow \infty$ of certain functions formed out of the FG coordinates $\tau ^n(X)$ and $\tau ^n(Y)$. A similar limit can be defined assuming $|U|>1$.

On the one hand, this relation agrees with an observation of [74], stating that FN coordinates arise as *spectral coordinates* (of which (*X*, *Y*) are one example) in the limit situation where $\hbar$ approaches an accumulation ray. The choice between $|U|<1$ and $|U|>1$ corresponds to the choice of a resolution in [74]. On the other hand, relation (5.25b) also establishes a suggestion from [64] on a more rigorous footing.

### Discussion

The reader will have noticed that the FN-type coordinates are defined somewhat more indirectly using exact WKB than the FG-type coordinates which are literally Borel summations of certain Voros symbols. In the case of the FN-type coordinates we did not define the coordinate $\sigma$ associated with a ring domain directly by Borel summation. Instead, we used Borel summation to define natural solutions to the differential equation defined by the quantum curve, and defined $\sigma$ from the holonomy of these solutions around the annulus *A* defined by the ring domain.

It is of course a natural question if $\sigma$ also admits a representation by Borel summation of a Voros symbol. Interesting results in this direction are provided by the recent work [55] devoted to the case $C=C_{0,2}$ discussed in this paper.^26^ The extensive numerical studies carried out in [55] can be taken as support for the conjecture that the Voros symbol associated with $\sigma$ is Borel summable along the positive real axis, at least when restricted to the subspace of opers.

The picture changes dramatically when considering a phase of $\hbar$ away from zero. One finds an infinite collection of wedges in the $\hbar$-plane in which the Voros symbols associated with $\sigma$ are again Borel summable. However, according to the results quoted above, one will generically find that the results of the Borel summations are represented by FG-type coordinates.

This may seem counterintuitive. However, one should notice that any angular rotation of $\hbar$ away from the positive real axis, as small as it may be, will inevitably cross infinitely many active rays. Starting from a wedge around a non-vanishing value of $\arg {\hbar }$ one will encounter the infinite sequence of flips discussed in the previous subsection when $\arg {\hbar }$ approaches zero. The observation that the FN-type coordinates can be understood as the limits of the sequence of FG-type coordinates associated with the wedges surrounding the positive real line seems to be perfectly consistent with the conjecture motivated by the results of [55] that the Borel summation of the Voros symbol associated with $\sigma (U,\hbar )$ is represented by FG coordinates in the wedges that are disjoint from the positive real axis, while it reproduces the function $\sigma (U,\hbar )$ when the Borel summation is performed along the positive real axis.^27^

## Theta Series Expansions from Exact WKB

Having established how the Exact WKB framework leads to natural choices of Darboux coordinates in different points in the moduli space of quantum curves, we now return to the theta-series expansions induced by these coordinates. On first sight it may seem that the picture we have previously found in the case of $C=C_{0,2}$ with two irregular singularities is somewhat different from the one established for $C=C_{0,4}$ in [36]. We will therefore revisit the latter case in this section, allowing us to demonstrate that an important basic feature is shared by the two cases: In both cases there exists a family of normalised tau-functions having representations of theta series type in terms of systems of Darboux coordinates for ${{\mathcal {M}}}_{{\textrm{ch}}}(C)$ which can be both of Fenchel–Nielsen and of Fock–Goncharov type.

### Theta Series Corresponding to FN-Type Coordinates from Exact WKB

We had seen in Sect. 3 that there exists a way to define normalised partition functions depending on the choice of a system of coordinates for the character variety ${{\mathcal {M}}}_{{\textrm{ch}}}(C)$. It was furthermore shown in Sect. 5 that exact WKB allows one to define distinguished sets of coordinates for a given region in the space of quantum curves. Our next goal is to show that the FN-type coordinates defined from exact WKB are among the coordinates $(\sigma ,\eta )$ that appear in the expansions of *theta series type* discussed in Sect. 3.

#### Case of $C_{0,4}$

Let us begin with the case of $C_{0,4}$. Recall that the definition of FN-type coordinates by exact WKB depends on the choice of a pair of Stokes graphs on the pairs of pants $C_i$, $i=1,2$, appearing in a given pants decomposition of $C_{0,4}$. To discuss a specific example let us consider the pants decomposition of *s*-type defined by cutting along a contour separating 0 and *z* from 1 and $\infty$. Fixing the *s*-channel defines $\sigma$ uniquely, but not $\eta$. In Sect. 3.2, we discussed several choices of $\eta$ leading to different theta series expansions, and discussed their relation. We will now show how certain pairs of coordinates $(\sigma ,\eta )$ encountered in Sect. 3.2 are tied to specific types of Stokes graphs by Exact WKB analysis, following observations and definitions of Sect. 5, and corroborating previous observations of [36].

Let us choose the Stokes graphs to be of type ${{\mathcal {S}}}_2$ (as defined in the discussion around Fig. 4) on both $C_1$ and $C_2$. According to the discussion in Sect. 3.1, one may use this set-up to define a FN-type coordinate $\eta _{22}$ related to $\eta _0$ by a relation of the form $e^{2\pi \textrm{i}\,\eta _{22} }\,{\textsf{n}}^{(1)}{\textsf{n}}^{(2)}=e^{2\pi \textrm{i}\eta _0 }$, with ${\textsf{n}}^{(i)}$ related to the Voros symbol $V^{(p_ib_i)}$ as ${\textsf{n}}^{(i)}=e^{-2V^{(p_ib_i)}}$ for $i=1,2$, respectively. This Voros symbol has recently been calculated in [14, 78]. Theorem 2.2 in [14] immediately implies the following formula for the corresponding normalisation constants

6.1 $$\begin{aligned}&{\textsf{n}}^{(2)}={\textsf{n}}_2(\sigma ,\theta _3,\theta _4),\qquad {\textsf{n}}^{(1)}={\textsf{n}}_2(\sigma ,\theta _2,\theta _1), \end{aligned}$$

6.2 $$\begin{aligned}&{\textsf{n}}_2(\vartheta _1,\vartheta _2,\vartheta _3)=\frac{2\pi }{\Gamma (2\vartheta _1)\Gamma (2\vartheta _1-1)} \frac{\Gamma (\vartheta _1+\vartheta _2+\vartheta _3)\Gamma (\vartheta _1+\vartheta _2-\vartheta _3)}{\Gamma (1-\vartheta _1+\vartheta _2+\vartheta _3)\Gamma (1-\vartheta _1+\vartheta _2-\vartheta _3)}. \end{aligned}$$

In this way, exact WKB defines a choice of normalisation for the $\eta$-coordinate, and a corresponding expansion for ${{\mathcal {T}}}$. What is not obvious from this definition, is whether or not the expansion of ${{\mathcal {T}}}$ defined by exact WKB takes the form of a generalised theta-series, or not.

To address this question, we may recall from Sect. 3.2 that coordinates $\eta$ appearing in expansions of generalised theta series type are related to the FN-type coordinate $\eta _0$ defined in Sect. 3.1.4 by a relation of the form

6.3 $$\begin{aligned}&e^{2\pi \textrm{i}\,\eta }=e^{2\pi \textrm{i}\,\eta _0 } \, \frac{{{\mathcal {N}}}^{(\eta )}(\sigma -1\,;\,{\underline{\theta }}\,)}{{{\mathcal {N}}}^{(\eta )}(\sigma \,;\,{\underline{\theta }}\,)}. \end{aligned}$$

It then suffices to note that the function $N_2$ defined in (3.28) satisfies

6.4 $$\begin{aligned} \frac{N_{2}(\vartheta _1,\vartheta _2,\vartheta _3)}{N_{2}(\vartheta _1-1,\vartheta _2,\vartheta _3)}= {\textsf{n}}_2(\vartheta _1,\vartheta _2,\vartheta _3) \end{aligned}$$

in order to see that the coordinate $\eta$ defined by setting in (6.3)

6.5 $$\begin{aligned} {{\mathcal {N}}}^{(\eta )}\equiv {N_2(\sigma ,\theta _2,\theta _1)N_2(\sigma ,\theta _3,\theta _4)}, \end{aligned}$$

is indeed the coordinate $\eta _{22}$ defined from exact WKB above. The tau function defined by this choice of normalisation for $\eta$ will be denoted as ${{\mathcal {T}}}^{({22})}$. It seems natural to assign ${{\mathcal {T}}}^{({22})}$ to the region in the moduli space of quantum curves where the pants decomposition of the Stokes graph defined by $(q,\hbar )$ produces a pair of Stokes graphs on $C_{0,3}$ of type $({{\mathcal {S}}}_2,{{\mathcal {S}}}_2)$.

#### Case of $C_{0,2}$

In a similar way, one may revisit the case $C=C_{0,2}$, asking in particular how the choice of normalisation for the FN-type coordinates appearing in Sect. 3.3 is related to the normalisation defined by Exact WKB.

To address this, we may start by noting that the weak coupling expansion (3.29) can easily be recast in the form

6.6 $$\begin{aligned}&{{\mathcal {T}}}^{(\eta )}(\,\sigma ,\eta \,;\,z\,)=\sum _{n\in {{\mathbb {Z}}}} \,e^{2\pi \textrm{i}n\,\eta }\,(-1)^n {{\mathcal {N}}}^{(\eta )}(\sigma +n){{\mathcal {F}}} (\,\sigma +n\,;\,z\,), \end{aligned}$$

with

6.7 $$\begin{aligned} {{\mathcal {N}}}^{(\eta )}(\sigma )=\frac{1}{G(1+2\sigma )G(1-2\sigma )}. \end{aligned}$$

The coordinate $\eta$ appearing in (6.6) has been defined in Sect. 3.4. In the general framework for the definition of FN-type coordinates described in Sect. 3.1, one may characterise the coordinate $\eta$ in terms of the normalisation factors ${\textsf{n}}^{(i)}$ explicitly given as

6.8 $$\begin{aligned} {\textsf{n}}^{(2)}={\textsf{n}}^{(1)}=\frac{\Gamma (1-2\sigma )}{\Gamma (2\sigma -1)}, \end{aligned}$$

as follows easily from equation (3.40).

Turning our attention to the normalisation fixed by Exact WKB, the relevant Voros symbols can be extracted from [78], giving for $\textrm{Re}(\sigma )>0$

6.9 $$\begin{aligned} {\textsf{n}}^{(2)}= {\textsf{n}}^{(1)}=\frac{2\pi }{\Gamma (2\sigma )\Gamma (2\sigma -1)}. \end{aligned}$$

Let us denote by ${{\tilde{\eta }}}$ the FN-type coordinate determined by this choice of normalisations. By adapting the discussion in Sect. 3.2, one can easily show that ${{\tilde{\eta }}}$ induces a generalised theta series expansion similar to the one in (3.29), but with ${\mathcal {N}}^{(\eta )}(\sigma )$ replaced by

6.10 $$\begin{aligned} {\mathcal {N}}^{({{\tilde{\eta }}})}(\sigma )= \frac{1}{(G(1+2\sigma ))^2}. \end{aligned}$$

It is interesting to note that the partition function ${{\tilde{{{\mathcal {Z}}}}}}(\sigma ,\Lambda )={{\mathcal {N}}}^{({{\tilde{\eta }}})}(\sigma ){{\mathcal {F}}}(\sigma ,\Lambda )$ defined with this choice of normalisation coincides with the topological string partition function computed with the help of the topological vertex, similarly to the example in [36] (see Appendix E [35]). This is not the case if ${{\mathcal {N}}}^{({{\tilde{\eta }}})}(\sigma )$ is replaced by the function ${{\mathcal {N}}}^{(\eta )}(\sigma )$ appearing in (3.29).

### Changes of FN-Type Coordinates for Fixed Pants Decomposition

Our next goal is to discuss how the definition for the normalised tau-functions defined by Exact WKB extends across the loci where the Stokes graphs change topological type. This will be related to the observations from Sect. 3.2 that there exist several choices of FN-type coordinates inducing generalized theta series expansions associated with a single pants decomposition, as anticipated in [36].

A few basic cases have to be discussed in this regard. The first type of changes of Stokes graphs is associated with the appearance of saddle trajectories inside one of the pairs of pants defined by the given pants decomposition. As a first example, let us consider the case where the Stokes graph on $C_2$ changes from type ${{\mathcal {S}}}_2$ to ${{\mathcal {S}}}_s$.

*6.2.1*

**Fig. 6:**
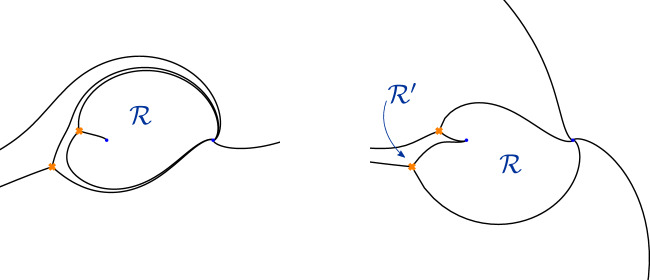
Transition from a Stokes graph of type ${{\mathcal {S}}}_2$ (left) to one of type ${{\mathcal {S}}}_s$ (right) on a three-punctured sphere defined by a factorization limit of $C_{0,4}$

We have given a definition for the case when both Stokes graphs on $C_{i}$, $i=1,2$ are of type ${{\mathcal {S}}}_2$ in the previous subsection. In the case where one of the Stokes graphs is of type ${{\mathcal {S}}}_s$ one needs to revisit the definition. Let us assume that the Stokes graph on $C_2$ has type ${{\mathcal {S}}}_s$. There are now two Stokes regions ${{\mathcal {R}}}$, ${{\mathcal {R}}}'$ around the puncture at $x=0$ created by the factorisation of $C_{0,4}$. One of them is simply the continuation of the Stokes region surrounding $x=0$ through the transition from ${{\mathcal {S}}}_2$ to ${{\mathcal {S}}}_s$, the other one is created in this transition, see Fig. 6.

In the definition of the Voros symbol fixing the normalisation, one now encounters an ambiguity in the choice of the branch point representing the starting point of the contour of integration. We are going to give here a prescription to fix this ambiguity, which is ‘as canonical as possible’. A broader discussion of this point can be found in Appendix F [35], which presents an alternative, less canonical prescription involving more choices.

According to the classification of Stokes graphs in [13], one will find the transition from ${{\mathcal {S}}}_2$ to ${{\mathcal {S}}}_s$ when the real part of the parameter $t:=C-B=\theta _3-\theta _4-\sigma$ becomes negative. The Stokes graph ${{\mathcal {S}}}_s$ has a pair of Stokes lines connecting the puncture at $x=0$ to two branch points $b_\pm$. Before the transition from region ${{\mathcal {S}}}_2$ to region ${{\mathcal {S}}}_s$, our general prescription leads us to a choice of normalisation fixed by the Voros symbol $V^{(b_20)}$, defined by an integration contour running from the branch point $b_2$ in ${{\mathcal {S}}}_2$ to the puncture at 0. After the transition we face two choices of normalisation, corresponding to Voros symbols $V^{(b_\pm 0)}$, whose integration contours run from the branch points $b_\pm$ in ${{\mathcal {S}}}_s$ to the puncture at 0.

*6.2.2*

It is important to notice that the transition from ${{\mathcal {S}}}_2$ to ${{\mathcal {S}}}_s$ involves a jump of topology for the Stokes graphs, shown in Fig. 6, which induces a jump of the Voros symbols. The Borel summation of the Voros symbol $V^{(b_20)}$ has recently been studied in [15]. It was found that the Borel summation of the Voros symbol $V^{(b_20)}$ will jump in the transition from ${{\mathcal {S}}}_2$ to ${{\mathcal {S}}}_s$. It follows from [15, Theorem 4.8] that the analytic continuation of the function ${\textsf{n}}^{(2)}_2$ defined by the Borel summation of the formal series $e^{-2V^{(b_20)}}$ in the chamber associated with ${{\mathcal {S}}}_2$ will be related to the functions ${\textsf{n}}^{(2)}_{s,\pm }$ defined by Borel summation of $e^{-2V^{(b_\pm 0)}}$ in the chamber associated with ${{\mathcal {S}}}_s$ by a relation of the form^28^

6.11 $$\begin{aligned} \frac{{\textsf{n}}^{(2)}_{s,\pm }(\sigma ,\theta _3,\theta _4)}{{\textsf{n}}^{(2)}_2(\sigma ,\theta _3,\theta _4)} =\frac{1}{1-e^{2\pi \textrm{i}\,\epsilon _{\pm }(\sigma +\theta _4-\theta _3)}}, \end{aligned}$$

with a sign $\epsilon _{\pm }\in \{1,-1\}$ depending on the path used to define the analytic continuation of the Voros symbol $V^{(b_20)}$. This sign is not arbitrary and can be determined unambiguously by a direct analysis, we will however fix it below taking an alternative route. The jump associated with the transition from ${{\mathcal {S}}}_2$ to ${{\mathcal {S}}}_s$ at $\textrm{Re}(t)=0$ is due to an analog of the Stokes phenomenon in the Borel summation of the Voros symbol.^29^

**Fig. 7:**
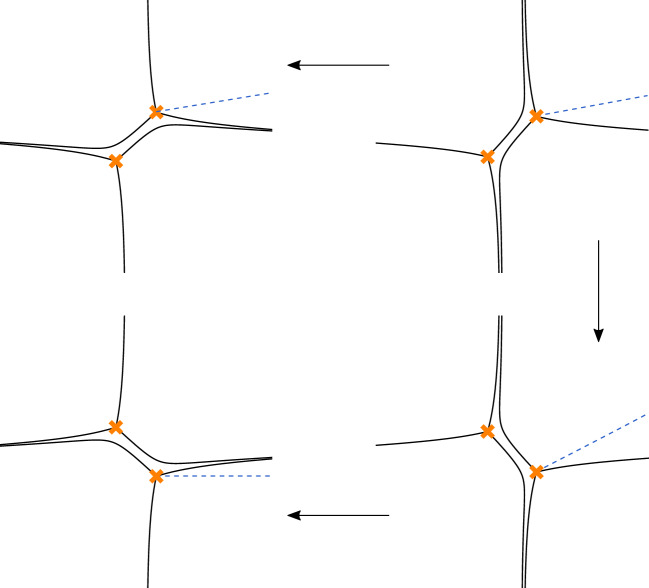
Transitions in the type of Stokes graph on $C_{0,3}$, from type ${{\mathcal {S}}}_i$ (top-right) to type ${{\mathcal {S}}}_s$ (top and bottom left). The anti-Stokes graph associates contours for the Voros symbol (the dashed blue line) beginning at two different branch points $b_\pm$. For convenience of illustration we depict the transition between regions ${{\mathcal {S}}}_3$ and ${{\mathcal {S}}}_s$ here, with the understanding that the transition between ${{\mathcal {S}}}_2$ and ${{\mathcal {S}}}_s$ is qualitatively identical

*6.2.3*

The difference between the two normalization factors of region ${{\mathcal {S}}}_s$ is due to the change of the choice of contour defining the Voros symbols according to our definitions above, see Fig. 7. In fact, it is evident from (6.11) that

6.12 $$\begin{aligned} {\textsf{n}}^{(2)}_{s,+}(\sigma ,\theta _3,\theta _4) = {\textsf{n}}^{(2)}_{s,-}(\sigma ,\theta _3,\theta _4) \cdot (- e^{2\pi \textrm{i}\,\epsilon _{-}(\sigma +\theta _4-\theta _3)}) \end{aligned}$$

Note that the exponent of the factor in brackets is (up to the overall sign) the Voros symbol associated with the shortest closed cycle running between the two branch points. This should indeed be expected since $V^{(b_-0)}-V^{(b_+0)} = V^{(b_-0)}+ V^{(0b_+)} = V^{(b_- b_+)}$.

The relations between normalisation factors ${\textsf{n}}^{(2)}_{2}, {\textsf{n}}^{(2)}_{s,+}, {\textsf{n}}^{(2)}_{s,-}$ are therefore completely under control, and consistent with each other. We have the two transitions from ${{\mathcal {S}}}_2$ into ${{\mathcal {S}}}_{s,\pm }$ described by (6.11) and the transition between ${{\mathcal {S}}}_{s,+}$ and ${{\mathcal {S}}}_{s,-}$ described by (6.12).

With this picture at hand, we are finally led to the following proposal to fix the normalisation of FN-type coordinates in the symmetric chamber ${{\mathcal {S}}}_s$. Instead of choosing between ${\textsf{n}}^{(2)}_{s,+}$ and ${\textsf{n}}^{(2)}_{s,+}$, we consider their geometric mean. In terms of Voros symbols, we define

6.13 $$\begin{aligned} V^{(s)} : = \frac{1}{2} \left( V^{(0 b_+)} + V^{(0 b_-)} \right) \end{aligned}$$

and set the corresponding normalisation coefficient to be ${\textsf{n}}^{(2)}_s=e^{-2V^{(s)}}$.^30^ This choice of normalisation has the advantage of being analytic throughout ${{\mathcal {S}}}_s$. It is clearly invariant under (6.12). With this choice of normalisation we find a unique jump between regions ${{\mathcal {S}}}_2$ and ${{\mathcal {S}}}_s$, replacing (6.11) by

6.14 $$\begin{aligned} \frac{{\textsf{n}}^{(2)}_{s}(\sigma ,\theta _3,\theta _4)}{{\textsf{n}}^{(2)}_2(\sigma ,\theta _3,\theta _4)} = - \frac{e^{\pi \textrm{i}\,(\sigma +\theta _4-\theta _3)}}{1-e^{2\pi \textrm{i}\,(\sigma +\theta _4-\theta _3)}} \,. \end{aligned}$$

Here, we have set $\epsilon _{\pm }=\pm 1$ in (6.11), this specialization will be motivated by an analysis of the asymptotic series for the Gamma function, coming up next. One may observe that (6.14) combines a jump due to the Stokes phenomenon with a jump in the leading term of the Voros symbol. The former is captured by (6.11) and corresponds to the two horizontal arrows in Fig. 7, while the latter is captured by (6.12) and arises from the change of basepoint for the integration contour between the left two frames of Fig. 7.

*6.2.4*

Given that formula (6.2) represents the Borel summation of $e^{-2V^{(b_20)}}$ one may alternatively derive (6.11) using the Borel summation of the asymptotic series of the Gamma functions appearing in formula (6.2), also discussed in Appendix D of [35]. In this way one finds that the prescription we have used to define the Voros symbols $V^{(b_\pm 0)}$ above is equivalent to defining the leading term $t\log t$ in the asymptotic series of $\log \Gamma (t)$ to be $t\log (-t)$. This will lead us to a convenient way to describe our continuation prescription, and to extend it to the remaining cases. Let us consider the function $\Gamma _B(w)$ defined as follows

6.15 $$\begin{aligned} \Gamma _B(w)=\left\{ \begin{aligned}&\Gamma (w)\qquad \qquad \quad \text {for}\;\;\textrm{Re}(w)>1,\\&\frac{2\pi }{\Gamma (1-w)} \qquad \quad \text {for}\;\;\textrm{Re}(w)<1, \end{aligned}\right. \end{aligned}$$

The function $\Gamma _B(w)$ is piece-wise analytic on ${{\mathbb {C}}}$, having jumps only along the imaginary axis. $\Gamma _B(w)$ represents the Borel summation of the asymptotic series for the Gamma-function $\Gamma (w)$ with leading term $w\log w$ defined by $w\log (-w)$ (denoted ${\check{\Gamma }}_0$ in Appendix D of [35]). The function $\Gamma _B(w)$ allows us to get the formula for the normalisation factor ${\textsf{n}}^{(2)}_s$ associated with the quantum curves having Stokes graphs ${{\mathcal {S}}}_s$ on one pair of pants simply by replacing the function $\Gamma (1-\sigma -\theta _4+\theta _3)$ in the expression for ${\textsf{n}}^{(2)}_2$ following from (6.2) by $\Gamma _B(1-\sigma -\theta _4+\theta _3)$.

*6.2.5*

For simplicity in the following discussion, we will restrict to cases with $\textrm{Re}(\sigma )\ge \frac{1}{2}$. The conditions for having a Stokes graph of type ${{\mathcal {S}}}_2$ on $C_2$ following from [13] imply that $\textrm{Re}(\theta _3-\theta _4)>0$. The only case which is left under these conditions is associated with the Stokes graph ${{\mathcal {S}}}_3$ on $C_2$. Proceeding along similar lines as before one will find that the normalisation factor ${\textsf{n}}^{(2)}_3$ associated with the quantum curves having Stokes graphs ${{\mathcal {S}}}_3$ on $C_2$ can be obtained by simply replacing the function $\Gamma (1-\theta _3-\theta _4+\sigma )$ in the expression for ${\textsf{n}}^{(2)}_s$ by $\Gamma _B(1-\theta _3-\theta _4+\sigma )$. The result for all three cases can be represented uniformly as

6.16 $$\begin{aligned} {\textsf{n}}^{(2)}(\sigma ,\theta _3,\theta _4)=\frac{2\pi }{\Gamma (2\sigma )\Gamma (2\sigma -1)} \frac{\Gamma (\sigma +\theta _3+\theta _4)\Gamma (\sigma +\theta _3-\theta _4)}{\Gamma _B(1-\sigma +\theta _3+\theta _4)\Gamma _B(1-\sigma +\theta _3-\theta _4)}. \end{aligned}$$

The cases not covered yet have $\textrm{Re}(\theta _4-\theta _3)>0$. In this case, one may proceed along the same lines as before, starting from the Stokes graph ${{\mathcal {S}}}_1$. The result can be represented in a uniform manner by using the function $\Gamma _B(w)$, as before.

According to the discussion in Sect. 3.1, there is a corresponding change of coordinates

6.17 $$\begin{aligned} e^{2\pi \textrm{i}\eta _{22} }= - e^{2\pi \textrm{i}\eta _{s2}} \frac{e^{\pi \textrm{i}\,(\sigma +\theta _4-\theta _3)}}{1-e^{2\pi \textrm{i}\,(\sigma +\theta _4-\theta _3)}} \end{aligned}$$

It is not hard to see that the corresponding difference generating function is ${{\mathcal {E}}}_{(2,s)}(\,\sigma ,{\underline{\theta }}\,)$ given as

6.18 $$\begin{aligned} {{\mathcal {E}}}_{(2,s)}(\,\sigma \,,{\underline{\theta }}\,)= {\mathcal {E}}(\sigma +\theta _4-\theta _3), \end{aligned}$$

with ${{\mathcal {E}}}(x)$ being functions constructed from *G*(*x*) as

6.19 $$\begin{aligned} {{\mathcal {E}}}(x)=(-2\pi \textrm{i})^{-x} \frac{G(1+x)}{G(1-x)}. \end{aligned}$$

We will interpret the function ${{\mathcal {E}}}_{(2,s)}$ as a transition function in a line bundle ${{\mathcal {L}}}_{\Theta }$ on the space ${{\mathcal {Z}}}$ of quantum curves associated with thickened neighbourhoods of the loci in ${{\mathcal {Z}}}$ where saddle connections of the type considered above appear.

With the help of the transition function ${{\mathcal {E}}}_{(2,s)}$, one can define a natural continuation of the tau-function associated with the pair of Stokes graphs $({{\mathcal {S}}}_2,{{\mathcal {S}}}_2)$ on $(C_2,C_1)$ into the region associated with the pair $({{\mathcal {S}}}_s,{{\mathcal {S}}}_2)$ of Stokes graphs.

**Fig. 8:**
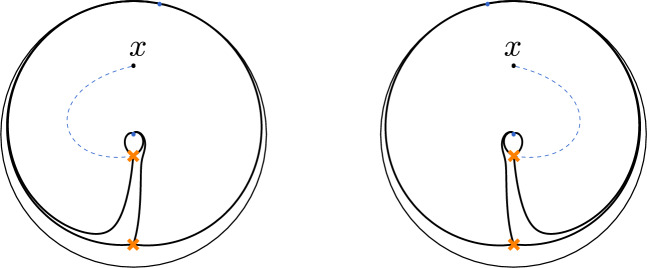
Contours of integration before and after the appearance of a saddle trajectory, which are related by composition with a cycle encircling the annulus

Another type of changes of the Stokes graphs associated with the appearance of saddle trajectories traversing the annulus has been discussed in Sect. 5. In this case, it is easy to see that the choice of contours defining the solutions $\psi _+^{(i)}$ defining the flat sections $Y_i$ cannot be continued through the loci where a saddle trajectory appears. One may note, however, that the contours of integration defining the leading term the WKB expansion for the normalisation factor before and after the appearance of a saddle trajectory are related by composition with a cycle encircling the annulus, as illustrated in Fig. 8. This will modify the normalisation factors ${\textsf{n}}^{(i)}$ by ${\textsf{n}}^{(i)}\rightarrow e^{\pm 2\pi \textrm{i}\sigma }{\textsf{n}}^{(i)}$, for $i=1,2$ corresponding to a change of coordinates $(\sigma ',\eta ')= (\sigma ,\eta \pm 2\sigma )$ according to the discussion in Sect. 5.5. The difference generating function ${{\mathcal {E}}}(\sigma ,{\underline{\theta }})$ associated with this case is found to be the function $e^{\mp 2\pi \,\textrm{i}\,\sigma ^2}$. Such difference generating functions represent transition functions of the line bundle ${{\mathcal {L}}}_\Theta$ associated with the loci where saddle trajectories traversing the annulus appear.

### The Real Slice

The space of quantum curves contains an interesting real slice represented by the so-called Jenkins–Strebel (JS-) differentials, quadratic differentials defining Stokes graphs having a maximal number of saddle trajectories. The Stokes graphs associated with generic JS-differentials decompose the surface *C* into ring domains, consisting either of annuli, or punctured discs around the punctures of *C*. A JS differential thereby defines a pants decomposition of *C*. Each pair of pants carries a Stokes graph having three edges connecting the two branch points. It is therefore necessary that the parameters $a_1$, $a_2$ and $a_3$ associated with any one of the pairs of pants appearing in this pants decomposition are purely real.

One should keep in mind that the parameters $\vartheta _i$ are related to $a_i$ as $\vartheta _i=\textrm{i} a_i/\hbar$. The differential $\hbar ^{-2}q_0(x)$ will then define a JS differential iff $\textrm{Im}(\hbar )=0$. On a four-punctured sphere $C=C_{0,4}$ defined by gluing two such pairs of pants one finds that there are accumulation rays at $\textrm{Im}(\hbar )=0$. In order to assign FN-type coordinates to JS differentials on *C* one has to choose a half-plane from which one approaches the real $\hbar$-axis. This corresponds to the choice of a resolution in the abelianisation approach. To be specific, we will in the following consider the case where $\textrm{Im}(\hbar )=0$ is approached from the upper half-plane. This implies that $\textrm{Re}(\vartheta _1)>0$. The other case can be treated in a very similar way.

According to the classification of Stokes graphs of [13] summarised in Sect. 5.1.2, one needs to distinguish chambers according to the sign of $a_i-a_{i+1}-a_{i+2}$, $i=1,2,3$, defining $a_i\equiv a_{i-3}$ for $i>3$. Introducing the notations

6.20 $$\begin{aligned} A=\vartheta _1+\vartheta _2-\vartheta _3,\qquad B=\vartheta _1+\vartheta _2+\vartheta _3,\qquad C=2\vartheta _1\,, \end{aligned}$$

the normalisation factors ${\textsf{n}}_i(\vartheta _1,\vartheta _2,\vartheta _3)$ associated with the Stokes graphs ${{\mathcal {S}}}_i$ are on the real slice for $i=1,2,3,s$, respectively, given as

6.21 $$\begin{aligned} \begin{aligned} {\textsf{n}}_s&= \frac{1}{\textrm{i}} \frac{\Gamma (A) \Gamma (B) \Gamma (C-A)}{\Gamma (C)\Gamma (C-1)\Gamma (B-C+1)} \\ {\textsf{n}}_1&= \frac{1}{2\pi } \frac{\Gamma (A) \Gamma (B)\Gamma (C-A) \Gamma (C-B)}{\Gamma (C)\Gamma (C-1)} \\ {\textsf{n}}_2&= \frac{2\pi \Gamma (A) \Gamma (B)}{\Gamma (C)\Gamma (C-1)\Gamma (A-C+1) \Gamma (B-C+1)} \\ {\textsf{n}}_3&= \frac{2\pi \Gamma (C-A) \Gamma (B)}{\Gamma (C)\Gamma (C-1)\Gamma (1-A) \Gamma (B-C+1)}. \end{aligned} \end{aligned}$$

This corresponds to assigning the functions $N_i(\vartheta _1,\vartheta _2,\vartheta _3)$ to Stokes graphs ${{\mathcal {S}}}_i$, for $i=1,2,3,s$, respectively, with $N_i$ for $i=1,2,3$ defined in (3.28), and

6.22 $$\begin{aligned} N_s(\vartheta _1,\vartheta _2,\vartheta _3)&=G(1+\vartheta _1+\vartheta _2+\vartheta _3) \frac{\prod _{j=1}^3 G(1+\vartheta _1+\vartheta _2+\vartheta _3 - 2\vartheta _j)}{(2\pi )^{\vartheta _1} \, G(1) \prod _{r=1}^3 G(1+2\vartheta _r)} \end{aligned}$$

Taking the product of the functions $N_i$ assigned to the pairs of pants defined by a JS differential in this way yields normalisation factors defining expansions of generalised theta series type for the tau-functions ${{\mathcal {T}}}$.

The tau-functions ${{\mathcal {T}}}$ defined in this way can be compared with the topological string partition functions computed in [36] with the help of the topological vertex. Picking the resolution specified above, it is easy to see that to each type of toric graph there corresponds a unique pair of Stokes graphs $({{\mathcal {S}}}^{(2)},{{\mathcal {S}}}^{(1)})$ and the constraints from positivity of the Kähler parameters in the toric geometry used for the geometric engineering in [36] correspond directly to the inequalities (5.4) characterising the Stokes graphs on $C_{0,3}$. We find perfect agreement, chamber by chamber.

## The Space of Quantum Curves

Based on the examples studied previously, we will in the rest of this paper formulate a proposal for the geometric description of the topological string partition functions in the more general class of cases associated with Riemann surfaces $C=C_{g,n}$ of genus *g* and *n* punctures. To simplify the discussion, we will only discuss regular singularities at the *n* punctures. The first step will be to revisit the notion of the quantum curve and the representation in terms of flat connections in this level of generality.

### Quantum Curves

On a Riemann surface $C_{g,n}$ with genus *g* and *n* punctures, we will consider projective $\hbar$-connections with *d* apparent singularities. A projective $\hbar$-connection can locally be represented by differential operators of the form $\hbar ^2\partial _x^2-q_\hbar (x)$, with $q_\hbar (x)$ transforming under a change of coordinate ${\tilde{x}}={\tilde{x}}(x)$ as

7.1 $$\begin{aligned} {q}_{\hbar }({x})=({\tilde{x}}'(x))^2{\tilde{q}}({\tilde{x}}(x))-\frac{\hbar ^2}{2}\{{\tilde{x}},x\},\qquad \{f,x\}=\frac{f'''}{f'}-\frac{3}{2}\left( \frac{f''}{f'}\right) ^2. \end{aligned}$$

We will assume that the functions $q_\hbar$ have convergent power series expansions in $\hbar$ of the form $q_\hbar (x)=\sum _{k=0}^{\infty }q_k(x)\hbar ^k$. If an apparent singularity is found in a chart with coordinate *x* at the position $x=u_r$, it means that $q_\hbar  (x)$ behaves near $x=u_r$ as

7.2 $$\begin{aligned} q_\hbar  (x)=\frac{3\hbar ^2}{4(x-u_r)^2}+\hbar \frac{v_r}{x-u_r}+{q}_{r} +{{\mathcal {O}}}(x-u_r), \end{aligned}$$

with $v_r$ and $q_{r}$ satisfying the system of relations

7.3 $$\begin{aligned} v_r^2=q_{r} , \qquad r=1,\dots ,d, \end{aligned}$$

ensuring that the monodromy of $\hbar ^2\partial _x^2-q_\hbar (x)$ is trivial in $\textrm{PSL}(2,{{\mathbb {C}}})$. Such projective $\hbar$-connections will be called quantum curves.

The difference between two projective $\hbar$-connections represented by functions $q_\hbar  (x)$ and $q_\hbar ^{*}(x)$ defines a quadratic differential. A projective $\hbar$-connection defines a projective structure on *C*, a system of local coordinates on local charts in *C* with transition functions all represented by Möbius transformations. One simply needs to use the ratio $w(x)=\eta _1(x)/\eta _2(x)$ of two linearly independent solutions of $(\hbar ^2\partial _x^2-q_\hbar  (x))\eta (x)=0$ as a new local coordinate in a given chart with coordinate *x*. With respect to the coordinate *w*, the projective $\hbar$-connection gets represented by $\hbar ^2\partial _w^2$. It is often convenient to assume from the outset that the coordinates *x* on open subsets of *C* are part of an atlas defining a projective structure. The functions $q_\hbar  (x)$ appearing in the local representation $\hbar ^2\partial _x^2-q_\hbar  (x)$ of a generic oper will then represent quadratic differentials $q_\hbar  (x) (dx)^2$. One may pick families of reference projective structures varying holomorphically over Teichmüller space. Changing the reference projective connection will modify $q_\hbar  (x)$ by terms of order $\hbar ^2$.

One may note that the quadratic differential $q_\hbar  (x)$ will generically be uniquely determined by the data $({\textbf{u}},{\textbf{v}})=\{(u_r,v_r);r=1,\dots ,d\}$ if $d\ge 3\,g-3+n$. Since $\textrm{dim}(H^0(C,K^2))=3\,g-3+n$, one then has sufficiently many equations (7.3) in order to fix the freedom to modify $q_\hbar  (x)$ by adding purely holomorphic quadratic differentials. We will mostly assume $d=3g-3+n$ in the following.^31^ We will in the following be interested in the space ${{\mathcal {Z}}}$ of quantum curves. We will also be interested in the dependence on the choice of complex structure on *C*. Picking local coordinates ${\textbf{z}}=(z_1,\dots ,z_{3g-3+n})$ for the moduli space ${{\mathcal {M}}}(C_{g,n})$ of complex structures on $C_{g,n}$ allows us to consider the collection $({\textbf{u}},{\textbf{v}};{\textbf{z}};\hbar )$ as a system of coordinates for the space ${{\mathcal {Z}}}$ of quantum curves.

In order to get a concrete parameterisation, let us take a pair $({\textbf{u}},{\textbf{v}})=\{(u_r,v_r);r=1,\dots ,d\}$. If $d=3\,g-3+n$ there generically exists a unique quadratic differential $q_0\equiv Q_0({\textbf{u}},{\textbf{v}})$ such that the spectral curve $\Sigma =\{(u,v)\in T^*C;v^2=Q_0(u)\}$ passes through $(u_r,v_r)$ for all $r=1,\dots ,d$. One may then define $Q_{\hbar ;{\textbf{u}},{\textbf{v}}}$ as $Q_{\hbar ;{\textbf{u}},{\textbf{v}}}=Q_0+\hbar Q_1+\hbar ^2 Q_2$, where $Q_i\equiv Q_i({\textbf{u}},{\textbf{v}})$, $i=1,2$, are the unique quadratic differentials having poles of first and second order at $x=u_r$, $r=1,\dots ,d$, respectively, satisfying^32^

7.4 $$\begin{aligned} \lim _{x\rightarrow u_r}\left( Q_1(x)-\frac{v_r}{x-u_r}\right) =0, \qquad \lim _{x\rightarrow u_r}\left( Q_2(x)-\frac{3}{4(x-u_r)}\right) =0, \end{aligned}$$

for $r=1,\dots ,d$. The quadratic differential $Q _{\hbar ;{\textbf{u}},{\textbf{v}}}$ defined in this way have the property that the expansion in powers of $\hbar$ truncates after the second order. The most general quantum curve with apparent singularities at $x=u_r$, $r=1,\dots ,d$, $d=3\,g-3+n$, which is at most quadratic in $\hbar$ can be represented in this way. We will call this the canonical form of the quantum curve.

It may be useful to consider $\hbar$-dependent changes of variables of the form $v_r=V_r({\textbf{u}},{\textbf{w}};\hbar )$, ${\textbf{w}}=(w_1,\dots ,w_d)$. Functions of the form $V_r= w_r+\hbar \nu _r({\textbf{u}})$ will preserve the feature that the $\hbar$-expansion of $q_\hbar$ contains only terms up to order $\hbar ^2$. There are many different coordinates for spaces of quantum curves, in general. Considering only quantum curves which are at most quadratic in $\hbar$ restricts possible $\hbar$-dependent changes of coordinates severely.

### Relations with $\hbar$-connections

A projective $\hbar$-connection defines an ordinary holomorphic $\hbar$-connection, locally represented in the form $\hbar \partial _x-A(x)$, with $$A(x)=\left( {\begin{smallmatrix} 0 &  q_\hbar \\ 1 &  0\end{smallmatrix}}\right) $$. $\hbar$-connections which are gauge equivalent to a connection of this form are called opers. It is usually assumed that $q_\hbar (x)$ is holomorphic in each local chart. We are here interested in opers which may have $d=3g-3+n$ apparent singularities, referred to as quantum curves.

There is a close relation between quantum curves and $\hbar$-connections on *C*, pairs $({\mathcal {E}},\nabla _\hbar )$, where ${{\mathcal {E}}}$ is a holomorphic bundle, and $\nabla _\hbar$ is a holomorphic $\hbar$-connection modulo gauge transformations. A $\hbar$-connection can locally be represented as $\nabla _\hbar =du(\hbar \partial _u-A(u))$, transforming under gauge transformations as $\nabla _\hbar =h\cdot {\tilde{\nabla }}_{\hbar }\cdot h^{-1}$. Instead of regarding $\hbar$ as a parameter we find it useful to consider it as a coordinate on the moduli space of $\hbar$-connections, the space ${{\mathcal {Z}}}_C$ of gauge equivalence classes $[\hbar ,{\mathcal {E}},\nabla _\hbar ]$ of triples $(\hbar ,{\mathcal {E}},\nabla _\hbar )$ defined by identifying pairs of triples related by gauge transformations. This space fibres over the moduli space ${{\mathcal {M}}}(C)$ of complex structures on *C*. The space ${{\mathcal {Z}}}$ of our interest will be the total space of this fibration.

For a given $\hbar$-connection, one can locally always find gauge transformations bringing it to oper form. In order to do this, we may start by representing ${{\mathcal {E}}}$ as an extension $0\rightarrow L\rightarrow {{\mathcal {E}}}\rightarrow L^{-1}\Lambda \rightarrow 0$. This corresponds to a representation of ${{\mathcal {E}}}$ in terms of transition functions which are all of the upper triangular form $$\big ({\begin{smallmatrix} \zeta &  \eta \\ 0 &  \zeta ^{-1}\delta \end{smallmatrix}}\big )$$, with $\zeta$ and $\delta$ being transition functions for the line bundles *L* and $\Lambda$, respectively. The local trivializations of such a representation allow one to represent $A\equiv A(x)$ in the form $$A=\left( {\begin{smallmatrix} a &  b \\ c &  -a\end{smallmatrix}}\right) $$, with matrix element *c* being a section of the line bundle ${\mathcal {K}}=K L^{-2}\Lambda$. Using such a representation, we can locally find gauge transformations relating $\nabla _\hbar =du(\hbar \partial _u-A(u))$ to a connection of oper form. The gauge transformation to oper form will be singular at the zeros $u_r$ of *c*, $r=1,\dots ,d$, leading to the $d=\textrm{deg}({\mathcal {K}})$ apparent singularities of the resulting oper. It is furthermore easy to see that the residue of an apparent singularity at $x=u_r$ is given as $-a(u_r)$, for $r=1,\dots ,d$.

This construction associates points $({\textbf{u}},{\textbf{v}})$ in the symmetric product $[T^*C]^{d}$ to $\hbar$-connections $\nabla _\hbar$ on extensions $0\rightarrow L\rightarrow {{\mathcal {E}}}\rightarrow L^{-1}\Lambda \rightarrow 0$. It can be shown that there exists a local inverse to this construction [44], assigning pairs $[{\mathcal {E}},\nabla _\hbar ]$ to points in the symmetric product $[T^*C]^{d}$. One may use it to define local coordinates for the moduli spaces ${{\mathcal {Z}}}_C$.

### Relations with Higgs Pairs

Higgs pairs are pairs $[{{\mathcal {E}}},\varphi ]$, with ${{\mathcal {E}}}$ being a holomorphic *G*-bundle, and $\varphi$ being an element of $H^0(C,\textrm{End}({{\mathcal {E}}})\otimes K_C)$. Let ${{\mathcal {M}}}_{\textrm{Hit}}(C)$ be the space of gauge equivalence classes $[{{\mathcal {E}}},\varphi ]$ of stable Higgs pairs $({{\mathcal {E}}},\varphi )$. The parameterization of $\hbar$-connections using points $({\textbf{u}},{\textbf{v}})\in [T^*C]^{d}$ is related to a similar parameterization of Higgs pairs $[{{\mathcal {E}}},\varphi ]$, to be outlined next.

To begin, let us note that one may parameterize $\hbar$-connections by picking families of reference $\hbar$-connections $\nabla _\hbar ^*$ varying holomorphically with the choice of ${{\mathcal {E}}}$, allowing us to define $\varphi =\nabla _\hbar  -\nabla _\hbar ^*$, an element of $H^0(C,\textrm{End}({{\mathcal {E}}})\otimes K_C)$. The maps $\Pi ^{*}:(\hbar ,{{\mathcal {E}}},\nabla _\hbar )\mapsto ({{\mathcal {E}}},\varphi )$ defined in this way can be used^33^ to define bi-holomorphic maps between open subsets of ${{\mathcal {Z}}}_C$ and ${{\mathcal {M}}}_{\textrm{Hit}}(C)\times {{\mathbb {C}}}$, respectively.

Convenient parameterisations for the classes $[{{\mathcal {E}}},\varphi ]$ of Higgs pairs are provided by the integrable structure of the Hitchin moduli space [69]. They can be found using the correspondence [69] between classes $[{{\mathcal {E}}},\varphi ]$ of Higgs pairs and pairs $(\Sigma ,{{\mathcal {L}}})$, with $\Sigma$ being the spectral curve defined by the characteristic equation $\textrm{det}(v-\varphi )=0$, and ${{\mathcal {L}}}$ being the bundle of eigenlines of $\varphi$ on $\Sigma$. Given $(\Sigma ,{{\mathcal {L}}})$ one may reconstruct $[{{\mathcal {E}}},\varphi ]$ from $(\pi _*({{\mathcal {L}}}),\pi _*(y))$, with $\pi :\Sigma \rightarrow C$ being the canonical projection, $\pi _*$ being the push-forward, and *y* being the canonical differential on $\Sigma$.

More explicit parameterizations can be defined as follows. Given $({{\mathcal {E}}},\varphi )$ with ${{\mathcal {E}}}$ being an extension $0\rightarrow L\rightarrow {{\mathcal {E}}}\rightarrow L^{-1}\Lambda \rightarrow 0$, and $$\varphi = \left( {\begin{smallmatrix} \varphi _0 &  \varphi _+ \\ \varphi _- &  -\varphi _0\end{smallmatrix}}\right) $$ one may again consider the collection $\{u_r;r=1,\dots ,d\}$ of zeros of $\varphi _-$, and define $v_r=-\varphi _0(u_r)$ for $r=1,\dots ,d$. One thereby assigns collections of points $({\textbf{u}},{\textbf{v}})=\{(u_r,v_r);r=1,\dots ,d\}$ in $T^*C$ to Higgs fields $\varphi$ on extensions $0\rightarrow L\rightarrow {{\mathcal {E}}}\rightarrow L^{-1}\Lambda \rightarrow 0$. Assuming that $u_r\ne u_s$ for $r\ne s$ one may use $({\textbf{u}},{\textbf{v}})$ to define a pair $(\Sigma ,{{\mathcal {L}}})$ by taking $\Sigma$ to be the curve in $T^*C$ passing through all points $(u_r,v_r)$, $r=1,\dots ,d$, and ${{\mathcal {L}}}:=\pi ^{*}(K^{-1}L){{\mathcal {O}}}_{\Sigma }(D)$, with *D* being the divisor $\sum _{r}\check{u}_r$ on $\Sigma$ satisfying $\pi (D)=\sum _{r=1}^du_r$ and $v(\check{u}_r)=v_r$, for $r=1,\dots ,d$. Observing that eigenvectors of $\varphi$ can be constructed as $$\left( {\begin{smallmatrix} v+\varphi _0 \\ \varphi _-\end{smallmatrix}}\right) $$ one may show that the pair $(\Sigma ,{{\mathcal {L}}})$ defined in this way agrees with the one defined by the construction above, see [44] for details.

Compared with Sect. 7.2, we see that we may parameterise families of Higgs pairs $({{\mathcal {E}}}_{{\textbf{u}},{\textbf{v}}} ,\varphi _{{\textbf{u}},{\textbf{v}}} )$ by collections $({\textbf{u}},{\textbf{v}})=\{(u_r,v_r);r=1,\dots ,d\}$ of points in $T^*C$. Representing ${{\mathcal {E}}}_{{\textbf{u}},{\textbf{v}}}$ as extensions, we may introduce a family of reference connections $\nabla ^*_{{\textbf{u}},{\textbf{v}}}=dx(\partial _x-A_*(x))$. Assuming $A_*$ to be of the form $$A_*=\left( {\begin{smallmatrix} a &  b \\ 0 &  -a\end{smallmatrix}}\right) $$ one may then consider the family of $\hbar$-connections $\nabla _{\hbar ;{{\textbf{u}},{\textbf{v}}}} = du(\hbar \partial _u-(\varphi _{{\textbf{u}},{\textbf{v}}} +\hbar A_*))$ associated with these data. As discussed in Sect. 7.2, one may gauge transform $\nabla _{\hbar ;{{\textbf{u}},{\textbf{v}}}}$ to oper form, defining a quantum curve from $({\textbf{u}},{\textbf{v}})$. This quantum curve can be represented in the form $\hbar ^2\partial ^2_x -Q _{\hbar ;{\textbf{u}},{\textbf{w}}}$ with ${\textbf{w}}$ related to ${\textbf{v}}$ by a simple change of variables ${\textbf{w}}={\textbf{w}}({\textbf{v}})$ which is linear $\hbar$.

### Integrable Structure

There is a projection $\Pi _0$ from ${{\mathcal {Z}}}$ to the Hitchin moduli space ${{\mathcal {M}}}_{\textrm{Hit}}(C)$ associating to a triple $(\hbar ,{\mathcal {E}},\nabla _\hbar )$ the Higgs pair $({\mathcal {E}},\varphi )$ with $\varphi =\nabla _0$. The moduli space ${{\mathcal {M}}}_{\textrm{Hit}}(C)$ has the structure of a torus fibration defined by means of the Hitchin map $h:{{\mathcal {M}}}_{\textrm{Hit}}(C)\rightarrow H^0(C,K_C^2)$ sending $[{{\mathcal {E}}},\varphi ]$ to the quadratic differential $\textrm{tr}(\varphi ^2)$. The equation $\frac{1}{2}\textrm{tr}(\varphi ^2)=q$, with $q\in H^0(C,K_C^2)$ being a fixed quadratic differential, defines submanifolds of ${{\mathcal {M}}}_\textrm{Hit}(C)$ which are abelian varieties. The composition $h\circ \Pi _0$ describes ${{\mathcal {Z}}}$ as a fibration over ${{\mathcal {B}}}_C\simeq H^0(C,K_C^2)$. We will also consider the subset ${{\mathcal {B}}}_C'\subset {{\mathcal {B}}}_C$ represented by quadratic differentials having simple zeros only, defining the subset ${{\mathcal {Z}}}'=(h\circ \Pi _0)^{-1}({{\mathcal {B}}}_C')\subset {{\mathcal {Z}}}$.

The base ${{\mathcal {B}}}_C$ carries a geometric structure called special geometry. Useful coordinates for the base ${{\mathcal {B}}}_C$ reflecting this structure are called homological coordinates, defined as periods of the canonical differential $\sqrt{q}$ on the spectral curve $\Sigma _q$ defined by *q*. To define a set of homological coordinates one needs to choose a canonical basis for $\Gamma =H_1(\Sigma _q^\circ ,{{\mathbb {Z}}})^-$, where the superscript indicates taking the odd part with respect to the exchange of sheets, and $\Sigma _q^\circ \subset \Sigma _q$ is the complement of the inverse image of the poles of *q*. A canonical basis for $\Gamma$ is represented by a collection of cycles $(\alpha ^1,\dots ,\alpha ^d;\beta _1,\dots ,\beta _d)$ on $\Sigma _q$ having intersection index $\alpha _r\circ \beta _s=\delta _{rs}$, where $d=\textrm{dim}({{\mathcal {B}}}_{C})=3g-3+n$ if $C=C_{g,n}$, allowing us to define

7.5 $$\begin{aligned} a^r=\int _{\alpha ^r}\sqrt{q},\qquad {\check{a}}_r=\int _{\beta _r}\sqrt{q}. \end{aligned}$$

There locally exists a function ${{\mathcal {F}}}({{\textsf{a}}})$, ${{\textsf{a}}}=(a^1,\dots ,a^d)$, such that ${\check{a}}_r=\partial _{a^r}{{\mathcal {F}}}({{\textsf{a}}})$. This function is called prepotential. The period matrix $\tau _{{\textsf{a}}}$ of $\Sigma$ is the matrix formed out of the second derivatives $\partial _{a^r}\partial _{a^s}{{\mathcal {F}}}({{\textsf{a}}})$ of ${{\mathcal {F}}}({{\textsf{a}}})$. The geometric structure called special geometry is encoded in a covering of ${{\mathcal {B}}}_C'$ with charts equipped with homological coordinates, and transition functions represented by the changes of homological coordinates associated with the elements of $\textrm{Sp}(2d,{{\mathbb {Z}}})$ describing changes of the canonical homology bases.

Canonically associated with the structure of special geometry is a torus fibration over ${{\mathcal {B}}}_C$ [53], with fibre over a point in ${{\mathcal {B}}}_C$ having coordinate values ${{\textsf{a}}}$ being the torus $\Theta _{{\textsf{a}}}={{\mathbb {C}}}^d/({{\mathbb {Z}}}^d+\tau _{{\textsf{a}}}\cdot {{\mathbb {Z}}}^d)$. The torus fibration defined in this way gives a concrete realization of the integrable structure canonically associated with the special geometry of the base ${{\mathcal {B}}}_C$. The special geometry of the base ${{\mathcal {B}}}_C$ is recovered from the integrable structure by representing the fibres over points $b\in {{\mathcal {B}}}_C$ as complex tori of the form $\Theta _b={{\mathbb {C}}}^d/({{\mathbb {Z}}}^d+\tau _b\cdot {{\mathbb {Z}}}^d)$, with $\tau _b$ being a symmetric $d\times d$ matrix. It can be shown [53] that locally over ${{\mathcal {B}}}_C$ there exist coordinates ${{\textsf{a}}}(b)$ and a function ${{\mathcal {F}}}({{\textsf{a}}})$ allowing one to represent the period matrix $\tau _b$ characterising the tori $\Theta _b$ as the matrix of second derivatives $\partial _{a^r}\partial _{a^s}{{\mathcal {F}}}({{\textsf{a}}})|_{{{\textsf{a}}}={{\textsf{a}}}(b)}$ of ${{\mathcal {F}}}({{\textsf{a}}})$. This is how the special geometry of the base is recovered from the integrable structure of ${{\mathcal {M}}}_{\textrm{Hit}}(C)$.

Recall that a point on ${{\mathcal {B}}}_C$ having coordinates ${{\textsf{a}}}$ represents a choice of a spectral curve $\Sigma _{{\textsf{a}}}$. The torus $\Theta _{{\textsf{a}}}$ represents the Prym variety parameterising the choices of line bundles ${{\mathcal {L}}}$ over $\Sigma _{{\textsf{a}}}$. For curves $\Sigma$ which are double coverings of a base curve *C* as considered here, we had seen that there is a natural correspondence between classes $[{{\mathcal {E}}},\varphi ]$ and *d*-tuples $({\textbf{u}},{\textbf{v}})$ of unordered points in $T^*C$. Taken together we see how the definition of the quantum curves $\hbar ^2\partial ^2_x -Q _{\hbar ;{\textbf{u}},{\textbf{v}}}$ is related to the integrable structure of the Hitchin system.

## Monodromy of Quantum Curves

In the examples studied previously, we had observed the crucial role played by distinguished coordinates of FN and FG types for the character varieties representing the monodromies of the differential operators $\hbar ^2\partial _x^2-q_\hbar  (x)$. As the next step in the formulation of our proposal we will now briefly outline how such coordinates can be defined in the more general cases associated with Riemann surfaces $C_{g,n}$.

### Holonomy

For any $\hbar \ne 0$, one may consider the holonomy of the $\hbar$-connection $\nabla _\hbar$, defining a representation $\rho :\pi _1(C)\rightarrow \textrm{SL}(2,{{\mathbb {C}}})$. The space of all such representations $\rho$ modulo overall conjugation defines the character variety ${{\mathcal {M}}}_{{\textrm{ch}}}(C)$. In this way we may define a map $\textsf{Hol}:{{\mathcal {Z}}}^\times \rightarrow {{\mathcal {M}}}_{{\textrm{ch}}}(C)$, with ${{\mathcal {Z}}}^\times$ being the moduli space of triples $[\hbar ,{\mathcal {E}},\nabla _\hbar ]$, with $\hbar \in {{\mathbb {C}}}^\times$. The character variety has a natural holomorphic symplectic form $\Omega _{\mathrm{\scriptscriptstyle G}}$ going back to Goldman.

The space ${{\mathcal {Z}}}$ can be regarded as a fibration over ${{\mathbb {C}}}$ by means of the projection $p:{{\mathcal {Z}}}\rightarrow {{\mathbb {C}}}$, $p[\hbar ,{\mathcal {E}},\nabla _\hbar ]=\hbar$. The fibres ${{\mathcal {M}}}_{\hbar }$, $\hbar \ne 0$, are moduli spaces of $\hbar$-connections with fixed parameter $\hbar$, and ${{\mathcal {M}}}_0\simeq {{\mathcal {M}}}_{\textrm{Hit}}(C)$. Each fibre ${{\mathcal {M}}}_\hbar$ has a natural symplectic form $\Omega _\hbar$. Restricting $\textsf{Hol}$ to a fibre ${{\mathcal {M}}}_\hbar$ defines a map $\textsf{Hol}_\hbar :{{\mathcal {M}}}_{\hbar }\rightarrow {{\mathcal {M}}}_{{\textrm{ch}}}(C)$ which is locally bi-holomorphic and symplectic [7, 70, 90].

We will later introduce collections of preferred systems of Darboux coordinates for ${{\mathcal {M}}}_{{\textrm{ch}}}(C)$. Such Darboux coordinates are collections of functions $({\textsf{x}},\check{{\textsf{x}}})$, ${\textsf{x}}=(x^1,\dots ,x^d)$, $\check{{\textsf{x}}}=({\check{x}}^1,\dots ,{\check{x}}^d)$ from ${{\mathcal {M}}}_{{\textrm{ch}}}(C)$ to ${{\mathbb {C}}}^\times$ allowing us to represent the symplectic form $\Omega _{\mathrm{\scriptscriptstyle G}}$ as

8.1 $$\begin{aligned} \Omega _{\mathrm{\scriptscriptstyle G}}=\sum _{r=1}^d {dx^r}\wedge {d{\check{x}}_r}. \end{aligned}$$

It is often useful to consider the coordinates $X^r=e^{2\pi \textrm{i}\,x^r}$, $\check{X}_r=e^{2\pi \textrm{i}\,{\check{x}}^r}$, $r=1,\dots ,d$. The coordinates $X^r$ and $\check{X}_r$ can for $r=1,\dots ,d$, be represented as the composition of a map ${\textsf{X}}:{{\mathcal {M}}}_{{\textrm{ch}}}(C)\rightarrow {{\mathbb {T}}}$, with ${{\mathbb {T}}}=({{\mathbb {C}}}^\times )^{2d}$, with the standard coordinate functions on $({{\mathbb {C}}}^\times )^{2d}$. The Darboux coordinates considered later will be distinguished from generic sets of Darboux coordinates by having important special properties.

We may finally compose the holonomy map $\textsf{Hol}$ with the coordinate functions ${\textsf{X}}$ to get locally defined maps ${{\mathcal {X}}}:{{\mathcal {Z}}}\rightarrow {{\mathbb {T}}}$ which can be regarded as collections of coordinates $({{\mathcal {X}}}^r,\check{{{\mathcal {X}}}}_r)$, $r=1,\dots ,d$, for ${{\mathcal {Z}}}$. By means of the local isomorphisms ${{\mathcal {Z}}}^\times \simeq {{\mathcal {M}}}_\textrm{Hit}(C)\times {{\mathbb {C}}}^{\times }$ mentioned above one gets families of holomorphic functions $({{\mathcal {X}}}^r(\hbar ),\check{{{\mathcal {X}}}}_r(\hbar ))$, $r=1,\dots ,d$, parameterised by points in ${{\mathcal {M}}}_{\textrm{Hit}}(C)$. One of the important features of the special class of Darboux coordinates to be considered here is the fact that the asymptotic behaviour for $\hbar \rightarrow 0$ is of the form

8.2 $$\begin{aligned} \log {{\mathcal {X}}}^r\simeq \frac{1}{\hbar }a^r+{{\mathcal {O}}}(\hbar ^0),\quad \log \check{{{\mathcal {X}}}}_r\simeq \frac{1}{\hbar }{\check{a}}_r+{{\mathcal {O}}}(\hbar ^0), \quad r=1,\dots ,d, \end{aligned}$$

with $a^1,\dots ,a^d$ and ${\check{a}}_1,\dots ,{\check{a}}_d$ being homological coordinates as introduced above. One should note, in particular, that equation (8.2) establishes a natural correspondence between the systems ${{\mathcal {X}}}$ of coordinates on ${{\mathcal {Z}}}$ considered later and systems of homological coordinates for ${{\mathcal {B}}}_C$.

### Coordinates Associated with Spectral Networks

The coordinates ${{\mathcal {X}}}$ on ${{\mathcal {Z}}}$ of our interest are obtained from coordinates on ${{\mathcal {M}}}_{{\textrm{ch}}}(C)$ with the help of the holonomy map $\textsf{Hol}$. We will therefore start by discussing very briefly the relevant sets of coordinates on ${{\mathcal {M}}}_{{\textrm{ch}}}(C)$. Two fairly well-known sets of coordinates for ${{\mathcal {M}}}_{{\textrm{ch}}}(C)$ are the Fock–Goncharov (FG)-type [52] and Fenchel–Nielsen (FN)-type coordinates. The FG coordinates are associated with triangulations of *C*. Coordinates of FN type are relatives of the complexifications of the classical Fenchel–Nielsen coordinates on the Teichmüller spaces ${{\mathcal {T}}}(C)$,^34^ sharing the main feature to be associated with pants decompositions of *C*.

While there are obvious differences in the way these types of coordinates are defined, they are ultimately more closely related than it may appear. An important common feature is the fact that both yield *rational* parameterisations of the trace functions, the generators of the ring of algebraic functions on ${{\mathcal {M}}}_{\textrm{ch}}(C)$. These coordinates therefore reflect the algebraic structure of ${{\mathcal {M}}}_{{\textrm{ch}}}(C)$ in a particularly simple way. This feature is well-known in the case of the FG coordinates, and has been noted in [112] for the case of the FN-type coordinates.

The relation between FG- and FN-type coordinates is closer than it might seem. It should be possible to show that the FG coordinates can be expressed as *rational* functions of FN-type coordinates,^35^ generalising the results of Sect. 4.4.

A unified framework for the definition of such coordinates is provided by the abelianisation program [74]. By introducing a certain graph ${{\mathcal {S}}}$ on *C* called spectral network one can set up a one-to-one correspondence between abelian connections on a cover $\Sigma _{{\mathcal {S}}}$ of *C*, and flat non-abelian connections on *C*. Coordinates for the abelian connections on $\Sigma _{{\mathcal {S}}}$ can thereby be used as coordinates for ${{\mathcal {M}}}_{{\textrm{ch}}}(C)$. By specialising the type of network used in this construction one can recover both FG- and FN-type coordinates. The coordinates associated with general spectral networks by abelianisation are hybrids of FG- and FN-type coordinates.

### Coordinates Associated with WKB Networks

As explained above, we are ultimately interested in the coordinates on ${{\mathcal {Z}}}$ obtained from coordinates on ${{\mathcal {M}}}_{{\textrm{ch}}}(C)$ by composition with the holonomy map $\textsf{Hol}$. One of the beautiful features of the coordinates on ${{\mathcal {Z}}}$ obtained in this way is the existence of natural domains of definition, obtained as follows. Each class $[\hbar ,{{\mathcal {E}}},\nabla _\hbar ]$ defines a pair $(q,\hbar )$ consisting of the quadratic differential $q=h\circ \Pi _0[\hbar ,{{\mathcal {E}}},\nabla _\hbar ]$ and $\hbar$. It is explained in [74] how each pair $(q,\hbar )$ defines a spectral network called WKB network. It is then very natural to assign the same type of coordinates to all $[\hbar ,{{\mathcal {E}}},\nabla _\hbar ]$ having a WKB network of the same topological type.

As discussed in [74], one finds for generic $(q,\hbar )$ WKB networks defining triangulations of *C* called WKB triangulations in [64]. Such networks are called Fock–Goncharov (FG)-type networks. At the opposite extreme one finds WKB networks decomposing *C* into a collection of annuli and punctured discs. Such networks are called Fenchel–Nielsen (FN) networks, naturally defining a pants decomposition of *C*. In between these extremes, there exist several hybrid types of networks having a varying number of ring domains. The space ${{\mathcal {Z}}}$ can be stratified according to the number of ring domains appearing in the Stokes graph.

One may naturally associate FG coordinates to the subset of ${{\mathcal {Z}}}$ having $(q,\hbar )$ defining a FG network of a fixed topological type. It is furthermore natural to associate FN-type coordinates to spectral networks of FN type. This program allows one to define coordinates for any saddle-free^36^ spectral network ${{\mathcal {W}}}$ defined by a quadratic differential *q*. The resulting coordinates will be of FG type if ${{\mathcal {W}}}$ has no ring domains, and will yield hybrid types of coordinates combining features of FG and FN types when there are ring domains.

The coordinates assigned to a WKB network using abelianisation have nice bonus features distinguishing them from generic coordinates for the space ${{\mathcal {Z}}}$. Most intensively studied is the subspace of ${{\mathcal {Z}}}$ represented by $\hbar$-opers $$\hbar \partial _u-\big ( {\begin{smallmatrix} 0 & q_\hbar \\ 1 &  0\end{smallmatrix}}\big )$$ without apparent singularities in the cases where the WKB network defined by $(q,\hbar )$, with $q=h\circ \Pi _0[\hbar ,{{\mathcal {E}}},\nabla _\hbar ]$, is of FG type. It follows from the results of [5, 84] that the functions ${{\mathcal {X}}}(\hbar )$ have asymptotic expansions in powers of $\hbar$ represented by the so-called Voros symbols. These asymptotic expansions are Borel-summable if the network defining the coordinates ${{\mathcal {X}}}(\hbar )$ is the FG-type WKB network defined by $(q,\hbar )$.

The case of the FN networks has been investigated less, an exception being the discussion in [72, Section 11]. However, as discussed in Sects. 5 and 6 one may use exact WKB to define canonical bases for the space of solutions to $\nabla _{\hbar }\eta =0$ on each of the three-punctured spheres defined by the pants decomposition associated with the given FN network. Using these canonical bases in the general framework for the definition of FN-type coordinates described in Sect. 3 yields a natural way to assign FN-type coordinates to WKB networks of FN type.

We conjecture that the coordinates ${{\mathcal {X}}}$ defined in this way all share the important feature that their leading asymptotics for $\hbar \rightarrow 0$ is of the form (8.2), establishing a correspondence with the set of homological coordinates $(a^r,{\check{a}}_r)$, $r=1,\dots ,d$, appearing on the right side of (8.2). It seems likely that the systems of coordinates ${{\mathcal {X}}}$ considered here are uniquely characterised by the collections of homological coordinates appearing in the asymptotics for $\hbar \rightarrow 0$.

### Problems of Riemann–Hilbert Type for the Coordinates ${{\mathcal {X}}}$

It is often useful to consider the variation of the spectral networks and the corresponding coordinates with respect to $\hbar$ for fixed quadratic differential *q*. This leads to a characterisation of the coordinates of our interest as solutions to a problem of Riemann–Hilbert (RH) type related to the ones studied in [64] and [24].

Considering the locus in ${{\mathcal {Z}}}$ with fixed $q=h\circ \Pi _0[\hbar ,{{\mathcal {E}}},\nabla _\hbar ]$, one may decompose the punctured plane ${{\mathbb {C}}}^\times$ parameterised by the coordinate $\hbar$ into wedges separated by rays running from the origin to infinity. The wedges are defined to be loci in ${{\mathbb {C}}}^\times$ where the spectral network has a fixed topological type. Rays separating two such wedges are often called active rays. They are characterised by the angle they span with ${{\mathbb {R}}}_+^\times \subset {{\mathbb {C}}}^\times$. The set of angles defining the active rays may have accumulation points. ${{\mathbb {C}}}^\times$ thereby gets decomposed as a disjoint union of the collections of wedges, active rays, and accumulation rays associated with $q\in H^0(C,K_C^2)$.

One may naturally assign coordinates of FG type to the wedges, noting that the corresponding spectral networks are of FG type. The coordinates associated with two wedges separated by a ray will be related by a bi-rational cluster transformation [5, 39, 84]. The collections of these bi-rational transformations are the key input data in the RH type problems characterising the FG-type coordinates according to [24, 64].

So far we have not assigned coordinates to the accumulation rays ${{\mathbb {R}}}_+^{\times }\hbar _{\textrm{ac}}$ yet. However, the corresponding spectral networks will have ring domains, defining a decomposition of *C* into a collection of annuli and bordered Riemann surfaces of simpler topological type. For $\hbar$ in a neighbourhood of $\hbar _\textrm{ac}$ one should be able to generalise the definition of FN-type coordinates from Exact WKB discussed in this paper in order to associate systems of hybrid FG–FN-type coordinates to pairs $(q,\hbar )$ with $\hbar$ in a neighbourhood of $\hbar _{\textrm{ac}}$.

From the point of view of the RH-type problems, one can view this as a way to complete the description by assigning coordinates to the accumulation rays, and to some neighbourhood around them. In order to see why it is natural to assign FN-type or hybrid coordinates to the accumulation rays one may recall from Sect. 5.6 that these coordinates can be understood as the *limits* of the family of FG coordinates associated with the infinite collection of wedges forming the neighbourhood of a given accumulation ray. It may be useful to include the rational transformations from FG- to FN-type coordinates into the collection of data defining the RH-type problems, viewing these transformations as a renormalised infinite product of the transformations associated with the active rays found in the neighbourhood of an accumulation ray.

### Choices of Polarisation

As anticipated in the notations $({{\mathcal {X}}}^r,\check{{{\mathcal {X}}}}_r)$, $r=1,\dots ,d$, we are ultimately interested in choices of coordinates coming with a choice of *polarisation*, expressed by the splitting into two subsets $\{{{\mathcal {X}}}^r;r=1,\dots ,d\}$ and $\{\check{{{\mathcal {X}}}}^r;r=1,\dots ,d\}$ of equal cardinality. This is an additional piece of data not canonically determined by the choice of a set of coordinates itself. A similar issue occurs in the case of the homological coordinates. The choice of a canonical homology basis uniquely determines the polarisation of the set of coordinates $(a^r,{\check{a}}_r)$, $r=1,\dots ,d$, indicated by our notations.

The choice of a polarisation is not canonically determined by the choice of a WKB network in general. A FG network allows us to define $6g-3+3n$ coordinates $X_e$ associated with the edges of the corresponding WKB triangulation. There are *n* monomials in the coordinates $X_e$ determined by the parameters associated with the punctures. There is no canonical way to define a system of coordinates with polarisation from the coordinates $X_e$, in general.

The situation is significantly better in the case of WKB networks of FN type. The definition of the FN-type coordinates given in [36, Section 6.1] for the case of $C_{0,4}$ can be generalised straightforwardly to general *n*-punctured Riemann surfaces *C*. It determines unambiguous choices of FN-type coordinates for certain WKB networks of FN type. This will be important for the definition of partition function discussed in Sect. 9.

## Tau-Functions

We finally return to the relation between tau functions and topological string partition functions. In previous sections, we have introduced key ingredients for our proposal, the spaces of quantum curves, and certain distinguished systems of coordinates on these spaces. We will now use these ingredients to propose a definition of fully normalised tau-functions allowing one to define topological string partition functions with the help of expansions of the form (1.1).

### Definition of the Tau-Functions

The isomonodromic tau-functions are usually defined as the generating functions for the Hamiltonians $H_r$, $r=1,\dots ,d$, generating the isomonodromic deformation flows. We will here propose a generalization of this definition to surfaces $C=C_{g,n}$ of arbitrary genus *g* which is based on the Riemann–Hilbert correspondence.

Over open subsets *U* of the moduli space ${{\mathcal {M}}}(C)$ of complex structures on *C* one may choose families of reference projective connections $\partial _x^2-t_U (x)$ with $t_{U} (x)\equiv t_{U} (x;{\textbf{z}})$ depending holomorphically on the points in ${{\mathcal {M}}}(C)$ represented by coordinates ${\textbf{z}}$ on *U*. To a given projective $\hbar$-connection $\hbar ^2\partial _x^2-t_\hbar (x)$ solving the Riemann–Hilbert problem one may associate the quadratic differential $Q_{U,\hbar } (x)=t_{\hbar } (x)-\hbar ^2 t_U (x)$. The space of quadratic differentials on *C* is canonically isomorphic to the cotangent space $T^*{{\mathcal {M}}}(S)$ of the moduli space ${{\mathcal {M}}}(S)$ of complex structures on a two-dimensional surface *S*. A basis $\{\partial _1,\dots ,\partial _d\}$ for the tangent space ${{\mathcal {T}}}(C)$ of ${{\mathcal {M}}}(S)$ at *C* therefore determines a basis $\{E_1,\dots ,E_d\}$ for the space of linear functions on $H^0(C,K_C^2)$. Assuming that the basis $\{\partial _1,\dots ,\partial _d\}$ is defined by a choice ${\textsf{z}}=(z_1,\dots ,z_d)$ of local coordinates on ${{\mathcal {M}}}(S)$ one may define the tau-function ${{\mathcal {T}}} _U({\textbf{z}})$ as the solution to the system of equations

9.1 $$\begin{aligned} \partial _{r}\log {{\mathcal {T}}} _U({\textsf{z}})=E_r(Q_{U,\hbar } ), \qquad r=1,\dots ,d. \end{aligned}$$

While we do not have a direct proof of the integrability of (9.1) at the moment, we may note that the existence of a function ${{\mathcal {T}}} _U({\textsf{z}})$ satisfying (9.1) follows from the free fermion construction of solutions to the Riemann–Hilbert problem outlined in Appendix, with $Q_{U,\hbar } (x)$ given by the corresponding expectation value of the energy–momentum tensor.

The family of reference projective connections $\partial _x^2-t_U (x)$ cannot be extended over all of ${{\mathcal {M}}}(C)$, in general. The reference projective connections $\partial _x^2-t_U (x)$ and $\partial _x^2-t_V(x)$ associated with two overlapping subsets *U* and *V* of ${{\mathcal {M}}}(C)$ may differ by a quadratic differential $Q_{UV}$ on the overlap. The corresponding tau-functions will therefore be related as

9.2 $$\begin{aligned} {{\mathcal {T}}} _U({\textsf{z}})=f_{UV} ({\textsf{z}}){{\mathcal {T}}} _V({\textsf{z}}), \end{aligned}$$

with $$f_{UV} ({\textsf{z}})$$ satisfying $$\partial _r\log f_{UV} ({\textsf{z}})=\hbar ^2 E_r(Q_{UV} )$$.

### Fully Normalised Tau-Functions

As the monodromy data are, by definition, conserved under the isomonodromic deformation flows it is natural to consider ${{\mathcal {T}}}$ as a function ${{\mathcal {T}}}(\mu ,{\textsf{z}})$ of (i) the deformation times ${\textsf{z}}=(z_1,\dots ,z_d)$, and (ii) the monodromy data $\mu$ which can be represented by points in ${{\mathcal {M}}}_{{\textrm{ch}}}(C)$. The definition (9.1) does not fully determine how ${{\mathcal {T}}}(\mu ,{\textsf{z}})$ depends on the monodromy data. Multiplying ${{\mathcal {T}}}(\mu ,{\textsf{z}})$ with an arbitrary function on ${{\mathcal {M}}}_{\textrm{ch}}(C)$ yields another solution of the defining equations (9.1). An important ingredient of our proposal is a natural way to fix this freedom, defining the fully normalised tau-functions discussed in the following.

Picking Darboux coordinates $x=({\textsf{x}},\check{{\textsf{x}}})$ for ${{\mathcal {M}}}_{{\textrm{ch}}}(C)$ allows one to represent the tau-functions ${{\mathcal {T}}}(\mu ,{\textsf{z}})$ in terms of functions ${{\mathcal {T}}}({\textsf{x}},\check{{\textsf{x}}};{\textsf{z}})\equiv {{\mathcal {T}}}(\mu ({\textsf{x}},\check{{\textsf{x}}}),{\textsf{z}})$ of the Darboux coordinates. We will propose that to each system of preferred coordinates $x=({\textsf{x}},\check{{\textsf{x}}})$ on ${{\mathcal {M}}}_{{\textrm{ch}}}$ there corresponds a fully normalised tau-function ${{\mathcal {T}}}_x({\textsf{x}},\check{{\textsf{x}}};{\textsf{z}})$. Discussing the dependence on the monodromy data we will often drop the dependence on ${\textsf{z}}$ in the notations, ${{\mathcal {T}}}_x({\textsf{x}},\check{{\textsf{x}}})\equiv {{\mathcal {T}}}_x({\textsf{x}},\check{{\textsf{x}}};{\textsf{z}})$.

The tau-functions ${{{\mathcal {T}}}}_x$ can be strongly constrained by the following system of difference equations. Note that the definition of the coordinates $x=({\textsf{x}},\check{{\textsf{x}}})$ involves the choice of a Lagrangian subspace of ${{\mathbb {T}}}$, parameterised by $x^r$, $r=1,\dots ,d$. The difference equations characterising the tau-functions are then

9.3a $$\begin{aligned}&{{\mathcal {T}}}_x({\textsf{x}},\check{{\textsf{x}}}+\delta _r)= {{\mathcal {T}}}_x({\textsf{x}},\check{{\textsf{x}}}), \end{aligned}$$

9.3b $$\begin{aligned}&{{\mathcal {T}}}_x({\textsf{x}}+\delta _r,\check{{\textsf{x}}})=e^{-2\pi \textrm{i}\,{\check{x}}_r}{{\mathcal {T}}}_x({\textsf{x}},\check{{\textsf{x}}}). \end{aligned}$$

It is clear that multiplication of ${{\mathcal {T}}}_x({\textsf{x}},\check{{\textsf{x}}})$ by a constant will preserve the validity of (9.3). The best we can hope for is therefore to be able to fix the normalisation of the functions ${{\mathcal {T}}}_x({\textsf{x}},\check{{\textsf{x}}})$ up to a multiplicative constant independent of ${\textsf{x}}$, $\check{{\textsf{x}}}$. One may take this freedom into account by considering equivalence classes $[{{\mathcal {T}}}_x({\textsf{x}},\check{{\textsf{x}}})]$ of functions ${{\mathcal {T}}}_x({\textsf{x}},\check{{\textsf{x}}})$ defined by the equivalence relation ${{\mathcal {T}}}_x({\textsf{x}},\check{{\textsf{x}}})\sim {\tilde{{{\mathcal {T}}}}}_x({\textsf{x}},\check{{\textsf{x}}})$ if there exists a constant $\nu _x\in {{\mathbb {C}}}^\times$ such that ${{\mathcal {T}}}_x({\textsf{x}},\check{{\textsf{x}}})=\nu _x {\tilde{{{\mathcal {T}}}}}_x({\textsf{x}},\check{{\textsf{x}}})$.

The difference equations (9.3) are equivalent to the fact that the functions ${{\mathcal {T}}}_x$ admit an expansion in the form of a generalised theta series,

9.4 $$\begin{aligned} {{\mathcal {T}}}_x({\textsf{x}},\check{{\textsf{x}}})= \sum _{{\textsf{n}}\in {{\mathbb {Z}}}^d}e^{2\pi \textrm{i}({\textsf{n}},\check{{\textsf{x}}})} Z_x({\textsf{x}}+{\textsf{n}}). \end{aligned}$$

We claim that we can associate fully normalised tau-functions ${{\mathcal {T}}}_x$ to any collection of coordinates *x* associated with spectral networks, be it FG type, FN type or hybrid type. A very brief outline of our approach can be found in Sect. 9.4 below. While the existence of expansions of the form (9.4) follows for coordinates of FN type from the free fermion construction of tau-functions outlined in Appendix, it is not obvious, in general, for coordinates of FG type. It should be stressed, however, that this issue does not affect the definition of fully normalised tau-functions ${{\mathcal {T}}}_x$ proposed here, based on the existence of difference generating functions for the changes between coordinates of FG and FN types discussed in Sect. 9.4.

### Difference Generating Functions for Changes of Coordinates

We will assume having chosen a cover of ${{\mathcal {M}}}_{{\textrm{ch}}}(C)$ with a set of charts $\{{{\mathcal {U}}}_{\imath };\imath \in {{\mathcal {I}}}\}$. Let ${{\mathcal {U}}}_\imath$ and ${{\mathcal {U}}}_\jmath$ be overlapping charts with coordinates $x_\imath =({\textsf{x}}_\imath ,\check{{\textsf{x}}}^\imath )$ and $x_\jmath =({\textsf{x}}_\jmath ,\check{{\textsf{x}}}^\jmath )$, respectively. The coordinates considered in this paper will be such that the equations ${\textsf{x}}_\imath ={\textsf{x}}_\imath ({\textsf{x}}_\jmath ,\check{{\textsf{x}}}^\jmath )$ can be solved for $\check{{\textsf{x}}}^\jmath$ in ${{\mathcal {U}}}_\imath \cap {{\mathcal {U}}}_\jmath$, defining a function $\check{{\textsf{x}}}^\jmath ({\textsf{x}}_\imath ,{\textsf{x}}_\jmath )$. Having defined tau-functions ${{\mathcal {T}}}_\imath ({\textsf{x}}_\imath ,\check{{\textsf{x}}}^\imath )$ and ${{\mathcal {T}}}_\jmath ({\textsf{x}}_\jmath ,\check{{\textsf{x}}}^\jmath )$ associated with charts ${{\mathcal {U}}}_\imath$ and ${{\mathcal {U}}}_\jmath$, respectively, there exist relations of the form

9.5 $$\begin{aligned} {{\mathcal {T}}}_\imath ({\textsf{x}}_\imath ,\check{{\textsf{x}}}^\imath )=F_{\imath \jmath }({\textsf{x}}_\imath ,{\textsf{x}}_\jmath ) {{\mathcal {T}}}_\jmath ({\textsf{x}}_\jmath ,\check{{\textsf{x}}}^\jmath ), \end{aligned}$$

on the overlaps ${{\mathcal {U}}}_{\imath \jmath }={{\mathcal {U}}}_\imath \cap {{\mathcal {U}}}_\jmath$. In order to ensure that both ${{\mathcal {T}}}_\imath$ and ${{\mathcal {T}}}_\jmath$ satisfy relations of the form (9.3) the function $F_{\imath \jmath }({\textsf{x}}_\jmath ,{\textsf{x}}_\jmath )$ must satisfy the two relations

9.6a $$\begin{aligned}&F_{\imath \jmath }({\textsf{x}}_\imath +\delta _r,{\textsf{x}}_\jmath )= e^{-2\pi \textrm{i}\,{{\check{x}}}^\imath _r} F_{\imath \jmath }({\textsf{x}}_\imath ,{\textsf{x}}_\jmath ), \end{aligned}$$

9.6b $$\begin{aligned}&F_{\imath \jmath }({\textsf{x}}_\imath ,{\textsf{x}}_\jmath +\delta _r)= e^{+2\pi \textrm{i}\,{{\check{x}}}^\jmath _r} F_{\imath \jmath }({\textsf{x}}_\imath ,{\textsf{x}}_\jmath ). \end{aligned}$$

We will call functions $F_{\imath \jmath }({\textsf{x}}_\jmath ,{\textsf{x}}_\jmath )$ associated with a change of coordinates $x_{\imath }=x_{\imath }(x_{\jmath })$ satisfying the relations (9.6) *difference generating functions*.

The relations between the function ${{\mathcal {T}}}_{\imath }$ and ${{\mathcal {T}}}_{\jmath }$ associated with different charts ${{\mathcal {U}}}_\imath$ and ${{\mathcal {U}}}_\jmath$ takes a slightly different form in the cases where ${\textsf{x}}_\imath ={\textsf{x}}_\jmath$, $\check{{\textsf{x}}}_\imath =\check{{\textsf{x}}}_\jmath +f_{\imath \jmath }({\textsf{x}}_{\imath })$. In this case one needs to modify (9.5) to

9.7 $$\begin{aligned} {{\mathcal {T}}}_\imath ({\textsf{x}}_\imath ,\check{{\textsf{x}}}^\imath )=F_{\imath \jmath }({\textsf{x}}_\imath ) {{\mathcal {T}}}_\jmath ({\textsf{x}}_\jmath ,\check{{\textsf{x}}}^\jmath ), \end{aligned}$$

with a function $F_{\imath \jmath }({\textsf{x}}_\imath )$ satisfying

9.8 $$\begin{aligned} F_{\imath \jmath }({\textsf{x}}_\imath +\delta _r)= e^{-2\pi \textrm{i}\,(\check{{\textsf{x}}}_\imath -\check{{\textsf{x}}}_\jmath )} F_{\imath \jmath }({\textsf{x}}_\imath )= e^{-2\pi \textrm{i}\,f_{\imath \jmath }({\textsf{x}}_{\imath })} F_{\imath \jmath }({\textsf{x}}_\imath ). \end{aligned}$$

Examples for relations of the form (9.7) have been presented in [36].

### Computing the Difference Generating Functions

We propose that there exists a cover $\{{{\mathcal {U}}}_{\imath };\imath \in {{\mathcal {I}}}\}$ of ${{\mathcal {M}}}_{{\textrm{ch}}}(C)$ having coordinates $x_\imath =({\textsf{x}}_\imath ,\check{{\textsf{x}}}^\imath )$ associated with the subsets ${{\mathcal {U}}}_{\imath }$ for $\imath \in {{\mathcal {I}}}$, together with a set of difference generating functions $F_{\imath \jmath }$ defined on the intersections ${{\mathcal {U}}}_{\imath \jmath }={{\mathcal {U}}}_\imath \cap {{\mathcal {U}}}_\jmath$ as introduced above. In Sect. 4 we discussed several examples of this structure, and computed the corresponding difference generating functions explicitly. We will now outline an approach to compute these in general.

We plan to give a detailed proof elsewhere. For the moment, let us note that a related problem has been encountered in the context of quantum Teichmüller theory. This theory was defined using coordinates of FG type in [34]. In order to give an unambiguous definition of the unitary operators representing the changes of coordinates associated with different triangulations one needs to choose polarisations. An elegant formalism for doing this was the basis of Kashaev’s approach to the quantisation of the Teichmüller spaces [88]. In order to establish the relation to conformal field theory it was necessary to construct quantised analogs of the changes of coordinates between coordinates of FG and FN types [110]. The operators representing the changes between different systems of FN coordinates defined thereby are the characteristic data of the generalised modular functor associated with the quantum Teichmüller theory [110]. The computation of explicit integral representations for these operators was completed in [112].

One may in our case proceed similarly. Instead of infinite-dimensional Hilbert spaces associated with systems of FN-coordinates, one will here find one-dimensional spaces, with tau-functions representing local bases. The Moore-Seiberg groupoid characterising the modular functor has vertices associated with pants decompositions, and edges corresponding to changes of pants decompositions. It will be represented by the difference generating functions associated with the changes of FN-coordinates. The use of a formalism like the one used in [110, 112] reduces the computation of the difference generating functions associated with the changes of coordinates to a small number of basic cases. For the passage from FG- to FN-type coordinates one may find^37^ the result with the help of [82, Proposition 4.3]. The difference generating functions for changes between two systems of FN-type coordinates can be reduced to the cases where $C=C_{0,4}$ and $C=C_{1,1}$, respectively. It has been found for $C=C_{0,4}$ in [79, 81]. The case $C=C_{1,1}$ can be reduced to $C=C_{0,4}$ by using the main idea from [71].

### Partition Functions Associated with Solutions of the Riemann–Hilbert Problem

Let ${{\mathcal {V}}}_w\subset {{\mathcal {Z}}}'$ be the set of all points in ${{\mathcal {Z}}}'$ such that the WKB network ${{\mathcal {W}}}_{q,\hbar }$ has topological type *w*. Let $x_w=({\textsf{x}}_w,\check{{\textsf{x}}}_w)$ be coordinates on ${{\mathcal {M}}}_{{\textrm{ch}}}(C)$ associated with ${{\mathcal {W}}}_{q,\hbar }$ by abelianisation. One should keep in mind that the definition of $x_w$ involves an additional choice not canonically determined by the topological type *w*, the choice of a polarisation represented by the splitting as $x_w=({\textsf{x}}_w,\check{{\textsf{x}}}_w)$. The corresponding coordinates on ${{\mathcal {V}}}_w\subset {{\mathcal {Z}}}'$ are denoted by ${{\mathcal {X}}}_w$.

Whenever the WKB network has FN type, it defines both a polarisation and a pants decomposition. It is then possible to use the gluing construction from CFT in order to define fully normalised tau-function ${{\mathcal {T}}}_{x_w}(\mu )$ for this case. A review of this construction n adapted to the cases of interest here can be found in the supplementary material in Appendix G of the arXiv-version [35] of this article. The key point to note is that, in many cases, there should also exist natural choices of polarisation for the FG coordinates associated with WKB triangulations, allowing us to define fully normalised tau-function ${{\mathcal {T}}}_{x_w}(\mu )$ for such cases as well. The Painlevé III case discussed in this paper is such a case.

Having associated a fully normalised tau-function ${{\mathcal {T}}}_{x_w}(\mu )$ to the coordinate system $x_w$ on ${{\mathcal {M}}}_{{\textrm{ch}}}(C)$, one may define a function $\Theta _w:{{\mathcal {V}}}_w\rightarrow {{\mathbb {C}}}$ such that

9.9 $$\begin{aligned} \Theta _w(\hbar ,{{\mathcal {E}}},\nabla _\hbar )={{\mathcal {T}}}_{x_w}\big (\textsf{Hol}[\hbar ,{{\mathcal {E}}},\nabla _\hbar ];{\textbf{z}}\big ). \end{aligned}$$

We conjecture that the coordinates ${{\mathcal {X}}}_w$ and the corresponding functions $\Theta _w$ can be analytically continued to coordinates defined in larger regions ${\hat{{{\mathcal {V}}}}}_w$ such that the collection of ${\hat{{{\mathcal {V}}}}}_w$ associated with all possible topological types *w* of spectral networks provides a cover of ${{\mathcal {Z}}}$.

One may then define the topological string partition function $Z_w^{\textrm{top}}$ on ${{\mathcal {B}}}_C\times {{\mathbb {C}}}^\times$ from the coefficients $Z_w({\textsf{x}})$ appearing in the theta-series expansions

9.10 $$\begin{aligned} {{\mathcal {T}}}_{x_w}({\textsf{x}},\check{{\textsf{x}}})= \sum _{{\textsf{n}}\in {{\mathbb {Z}}}^d}e^{2\pi \textrm{i}({\textsf{n}},\check{{\textsf{x}}})} Z_{w}({\textsf{x}}+{\textsf{n}}). \end{aligned}$$

To this aim let us recall that the coordinates ${{\mathcal {X}}}$ are related to a system of homological coordinates $({\textsf{a}},\check{{\textsf{a}}})$ via (8.2). We may then define

9.11 $$\begin{aligned} Z_w^{\textrm{top}}({{\textsf{a}}},\hbar )=Z_w ({\textstyle \frac{1}{\hbar } }{{\textsf{a}}}_w). \end{aligned}$$

We are here taking advantage of the fact that there is a natural one-to-one correspondence between the homological coordinates ${{\textsf{a}}}$ and the coordinates ${{\textsf{x}}}$ parameterising Lagrangian subspaces of ${{\mathcal {Z}}}$ defined by fixing $\check{{{\textsf{x}}}}$.

### A Holomorphic Line Bundle over ${{\mathcal {Z}}}'$

Let $\{{\hat{{{\mathcal {V}}}}}_{\imath };\imath \in {{\mathcal {I}}}\}$ be a cover ${{\mathcal {Z}}}'$ having charts ${\hat{{{\mathcal {V}}}}}_\imath \equiv {{\hat{{{\mathcal {V}}}}}}_{w_\imath }$ equipped with Darboux coordinates ${{\mathcal {X}}}_\imath ={{\mathcal {X}}}_{w_\imath }$, projecting to subsets $U_\imath \subset {{\mathcal {M}}}(C)$ equipped with reference projective connections $\partial _x^2-t_\imath (x)$, and let the corresponding partition functions be $\Theta _{\imath }\equiv \Theta _{w_\imath }$ for $\imath \in {{\mathcal {I}}}$. Whenever there are charts ${\hat{{{\mathcal {V}}}}}_\imath$, ${\hat{{{\mathcal {V}}}}}_\jmath$ such that ${\hat{{{\mathcal {V}}}}}_{\imath \jmath }={{\hat{{{\mathcal {V}}}}}}_{\imath }\cap {{\hat{{{\mathcal {V}}}}}}_{\jmath }\ne \emptyset$ one may define on ${\hat{{{\mathcal {V}}}}}_{\imath \jmath }$ the function $\Phi _{\imath \jmath }=\Theta _{\imath }/\Theta _{\jmath }$. The function $\Phi _{\imath \jmath }$ factorises as

9.12 $$\begin{aligned} \Phi _{\imath \jmath }=f_{\imath \jmath } (\textbf{z})F_{\imath \jmath }(\textsf{Hol}[\hbar ,{{\mathcal {E}}},\nabla _{\hbar }]), \end{aligned}$$

where $f_{\imath \jmath }({\textbf{z}})\equiv f_{U_\imath U_\jmath } ({\textbf{z}})$, with $f_{UV} ({\textbf{z}})$ being defined in (9.2), and $F_{\imath \jmath }(\textsf{Hol}[\hbar ,{{\mathcal {E}}},\nabla _{\hbar }])$ being the difference generating function associated with the change of coordinates between the spectral coordinates associated with ${\hat{{{\mathcal {V}}}}}_\imath$ and ${\hat{{{\mathcal {V}}}}}_\jmath$, respectively. We claim that the collection of all such functions $\Phi _{\imath \jmath }$ defines a holomorphic line bundle ${{\mathcal {L}}}_\Theta$ on ${{\mathcal {Z}}}'$. This line bundle comes with a collection of preferred holomorphic sections $\{\Theta _{\imath },\imath \in {{\mathcal {I}}}\}$, defining a connection $\nabla _\Theta$ on ${{\mathcal {L}}}_\Theta$.

One should note that this claim is realized in a somewhat subtle way. The relation between tau-functions and free fermion conformal blocks described in Sect. 2 and in Appendix allows us to take advantage of some insights and results from conformal field theory (CFT). Changes of pants decompositions defining FN-type coordinates represent the Moore-Seiberg groupoid. The representations of this groupoid coming from CFT have changes of pants decompositions realized by the products of two operations. One of them is independent of the complex structure, in our case represented by the functions $F_{\imath \jmath }$ in (9.12). The definition of conformal blocks, on the other hand, involves choices of projective structures on Riemann surfaces, leading to complex-structure dependent factors [54, 111, 112], in (9.12) represented by the functions $f_{\imath \jmath }$. A discussion quite similar to the one in [54, 112] shows that neither $f_{\imath \jmath }$ nor $F_{\imath \jmath }$ would define line bundles by themselves, as the respective cocycle conditions will be violated on Riemann surfaces of higher genus. The violation of the cocycle conditions by the complex structure independent factors $F_{\imath \jmath }$ is universal and completely determined by the central charge [54]. It must therefore coincide with the one calculated in [112], see equations (6.34e) and (8.22). However, the existence of local sections defined by the gluing construction of conformal blocks implies that the violations of the cocycle conditions for $f_{\imath \jmath }$ and $F_{\imath \jmath }$ must exactly cancel each other.

We now see why it was important to complete the collection of FG-type coordinates defined by Borel summation of the WKB expansion by including FN- and hybrid-type coordinates. Without this completion there would be no natural way to assign topological string partition functions to the regions in ${{\mathcal {Z}}}$ associated with spectral networks of FN type. In the application to topological string theory, these regions appear to be of particular interest, corresponding to regions admitting weak-coupling expansions. Let us furthermore recall that networks of FN type determine a canonical polarisation for the coordinates associated with them. This is important for having an unambiguous definition of the corresponding partition functions $\Theta _w$. For FG-type networks, one does not have a canonical polarisation, forcing us to make additional choices.

## Summary and Relations to Other Lines of Research

We shall here offer a brief summary and a discussion of relations to other lines of research.

### Summary

We observed a direct correspondence between choices of coordinates and possible definitions for fully normalised free fermion partition functions. When applied to the FN-type coordinates associated with weak coupling regions by exact WKB, we recovered the partition functions computed by the topological vertex. Weak coupling regions in the space of quadratic differentials are characterised by the appearance of ring domains for certain critical phases $\theta _*$ of the parameter $\hbar$. Given a quadratic differential *q* in the weak coupling region, there exists a finite neighbourhood of the phase $\theta _*$ within which we can use the ring domains defined by $(q,\rho e^{i\theta _*})$, $\rho \in {{\mathbb {R}}}_+$, to define coordinates of FN type with the help of the decomposition of the surface *C* defined by the ring domains appearing at $\textrm{arg}(\hbar )=\theta _*$. It may then seem natural to start by defining the partition functions in a given weak coupling region, and extend the definition to a larger domain using the changes of normalisation induced by the coordinate transformations associated with changes of the Stokes graph.

One may note, however, that there are regions in the space of quadratic differentials where no ring domain occurs. Such regions are called strong coupling regions. The results of [82] give a candidate for the definition of the partition functions in this region. We had seen that there exist changes of Fock–Goncharov coordinates preserving the feature that there exist expansions of generalised theta-series type. In this way, we can generate other candidates for partition functions in the strong coupling region. A general issue to be addressed in any attempt to assign partition functions to coordinates of Fock–Goncharov type is the need to choose a polarisation.

The changes of normalisation induced by the changes of coordinates have been used to define the line bundle ${{\mathcal {L}}}_{\Theta }$. Having defined a partition function in a weak coupling region gives a distinguished holomorphic section $\Theta$ of the line bundle ${{\mathcal {L}}}_{\Theta }$. It does not matter which weak coupling region is used since the relations between the partition functions assigned to different weak coupling regions are defining the corresponding transition functions of ${{\mathcal {L}}}_{\Theta }$. The functions $\Theta _\imath$ representing the section $\Theta$ in the weak coupling region with label $\imath$ are identified with the partition functions associated with the same region.

In order to define actual partition *functions* in strong coupling regions, one would need to make additional choices. For each topological type of Stokes graph one would need to choose a polarisation for the Fock–Goncharov coordinates associated with it. It is not clear to us at the moment if there is a natural way to do this.

There are, in the end of the day, always certain ambiguities in the definition of partition functions in quantum field theory and (topological) string theory. These ambiguities include, on the one hand, choices entering the definition of the classical part represented by the prepotential, in our case concretely represented by the necessity to choose a canonical basis for the homology of the spectral curve. Other choices can be related to the choice of a UV duality frame, here represented by the choice of a pants decomposition. Physically relevant information appears to be encoded in the transition functions between different regions in the moduli space. These transition functions in particular encode the spectrum of BPS states, as will be discussed more in the following. From this point of view it seems tempting to view the partition functions associated with FN- and FG-type coordinates as different ways to package the same physical information.

In view of the intended application to topological string theory one should keep in mind that the A-model topological string (Gromov–Witten (GW)) theory comes with preferred reality conditions, given by the reality of the Kähler parameters. The topological string partition functions should certainly be defined on this real slice, possibly allowing analytic continuations to the complexified Kähler cones. This real slice gets represented by the JS-differentials in the cases studied in this paper. Our discussions indicate that the FN-type coordinates are relevant in this case. It is furthermore important in this context that there is an analytic continuation away from the real slice represented by JS differentials allowing us to extend the definitions of the partition functions to the complexified Kähler cones.

The role of the FG coordinates needs to be better understood from this point of view. This type of coordinates might play a role in dual representations of the topological string partition functions related to the counting of BPS degeneracies. Some of these connections will be discussed next.

### Relations to the Spectrum of BPS States

A key role in our paper is played by the changes of coordinates associated with non-generic topological types of the Stokes graphs. The relevant non-generic types of Stokes graphs include the appearance of saddle trajectories, and the appearance of closed trajectories. Work initiated by [64] has revealed a profound relation between Stokes graphs of these types and BPS states in the class ${{\mathcal {S}}}$-theories associated with the Riemann surface *C* and the Lie algebra $A_1$.

A general framework for the description of the spectrum of BPS states in $d=4$, ${\mathcal {N}}=2$ supersymmetric field theories has previously been proposed in [63]. A key role in the approach of [63] is played by certain Darboux coordinates for the moduli space of vacua of the three-dimensional field theories obtained by circle compactification of the $d=4$, ${\mathcal {N}}=2$ supersymmetric field theory of interest. These Darboux coordinates can be characterised as solutions of a problem of Riemann–Hilbert (RH) type, the key data in the formulation of the relevant RH-type problem being the changes of coordinates generated by changes of the phase of the hyperkähler parameter $\zeta$.

The work [64] offers a large class of interesting examples for the description of the spectrum of BPS states previously proposed in [63]. The Darboux coordinates considered in [63] have been identified with Fock–Goncharov coordinates for the case of class ${{\mathcal {S}}}$-theories considered in [64]. These coordinates are related to the coordinates considered in our paper in the conformal limit [37, 40, 62]. This induces a direct relation between the data defining the RH-type problems arising in the study of BPS spectra with the data defining the line bundle ${{\mathcal {L}}}_{\Theta }$ introduced in our paper.

The relation between the spectrum of BPS states and Darboux coordinates can be made particularly transparent using the nonlinear integral equations of TBA type satisfied by the Darboux coordinates [63]. The conformal limit of these integral equations first discussed in [62] should be relevant for the cases studied in this paper. For the case $C=C_{0,2}$ considered above such integral equations have been studied in particular in [55, 62, 75]. Both [75] and [55] propose nonlinear integral equations for FN-type coordinates.^38^

One may expect that the generalisations of Donaldson–Thomas (DT) theory developed by Kontsevich, Soibelman, Joyce and others give a widely applicable framework for the mathematical description of BPS states in string theory which should be related to the one in SUSY field theory by geometric engineering [32]. RH-type problems solved by Darboux coordinates on the space of stability conditions have been formulated in this context in [24]. Projective connections on Riemann surfaces provide large families of examples for the solutions to these RH-type problems, as conjectured in [24], and more recently proven in [6]. Exact WKB relates the transitions between coordinate systems representing solutions to these RH-type problems to the DT invariants associated with spaces of quadratic differentials on Riemann surfaces *C* [23]. The quantum curves studied in this paper generalise the projective connections considered in [6, 24] by allowing apparent singularities. We conjecture that the results of [6, 24] can be generalised to this case. This would represent important groundwork for realising some of the proposals made in this paper in the case of general Riemann surfaces *C*.

In most of the above-mentioned studies of the spectrum of BPS states, the Fock–Goncharov coordinates have played a key role. Such coordinates are assigned to certain wedges in the $\hbar$-plane, in the solution provided by projective connections represented by the Borel summations of Voros symbols. A variant of the Stokes phenomenon makes the Borel summations jump across the rays in the $\hbar$-plane separating the wedges [39]. The coordinate transformations representing these jumps represent key data entering the formulation of the RH-type problems introduced in [24, 63]. Our results suggest that there exist natural completions of the solutions to the RH-type problems proposed in [24, 64] assigning FN-type coordinates to the accumulation rays. The transformations between coordinates of FN and FG types can be thought of as infinite products of the coordinate transformations associated with the infinite sequence of rays surrounding an accumulation ray.

Bridgeland has recently proposed geometric structures encoding DT invariants called Joyce structures [25]. Joyce structures can be expressed in terms of complex hyperkähler structures on the total spaces of the tangent bundles on the spaces of stability conditions [27]. There exist natural generating functions for these geometric structures called tau-functions in [29].

The stability conditions on spaces of quadratic differentials defined in [23] have been shown in [28] to define Joyce structures closely related to isomonodromic deformations of bundles with connections. The tau-functions associated with these Joyce structures coincide with the isomonodromic tau-functions [29]. These results exhibit the theory of Joyce structures as a natural geometric framework for the relations between spectra of BPS states and the objects discussed in this paper. The relation between Joyce structures and the generalized theta series expansions underlying the approach discussed in this paper should be clarified.

String theory suggests that the topological string partition functions studied in this paper are related to generating functions for the degeneracies of bound states of D0, D2 and D6-branes [46, 98]. It seems natural to expect that such degeneracies should display wall-crossing phenomena somewhat analogous to the phenomena studied in the case of toric CY in [87], and to the wall-crossing of framed BPS states studied in [32, 65]. From this point of view one might suspect that the relations predicted in [42] only hold in weak coupling chambers of the space of stability data, being replaced by more subtle relations in other chambers. However, in view of the results presented in this paper we feel tempted to speculate that the jumping of the free fermion partition functions $Z_{\textrm{ff}}$ discussed here represents the framed wall-crossing behaviour for the cases at hand in all chambers.

Of particular interest in this regard seem to be the transitions between partition functions associated with FG- and FN-type coordinates, respectively. While it is not clear if FG-type expansions exist, and would then be related to topological string partition functions in the sense of GW theory, it still seems reasonable to suspect that the coefficients of the strong coupling expansions might have an interpretation as generating functions for degeneracies of BPS states.

Among the ingredients in our proposal, it seems to us that the characterisation of the partition functions with the help of RH-type problems solved by distinguished coordinates on the spaces of stability data proposed above has a particularly high potential for generalisations beyond the family $Y_\Sigma$ of local CY, and possibly even beyond the case of local CY.

### Relation to Spectral Determinants

Another approach to the definition of a non-perturbative completion of the topological string partition function is based on the spectral determinants of the quantum curve [56]. This approach has been successfully applied to various toric CY. The geometric engineering of SUSY field theories in string theory suggests that the resulting partition functions might be related to the partition functions studied in this paper. In [20], it has indeed been shown that the limit used in the geometric engineering of pure SU(2) SYM relates the spectral determinant associated with the relevant toric CY to the tau-function of Painleve III at $\eta =0$.

The spectral determinant associated with the quantum curve relevant for the case $C=C_{0,2}$ has recently been studied in [55]. The result is given by an explicit expression (formula (5.6) in [55]) in terms of quantum periods denoted $\Pi ^{\textrm{ex}}_{A,B}$ in [55]. It is possible to show that these quantum periods are closely related to the FN-type coordinates introduced for the case $C=C_{0,2}$ in our paper. It seems intriguing to observe that the formula for the spectral determinant proposed in [55] resembles strongly our formula (3.45) expressing the Fock–Goncharov coordinates in terms of FN-type coordinates.

In this regard, it seems interesting to observe that the function $D(U,\hbar )=e^{\pi \nu (U,\hbar )}$ defined by evaluating the FG coordinate ${Y}^{-1}$ introduced in Sect. 3.6 on the restriction of the monodromy map to the submanifold represented by opers coincides with the spectral determinant of the differential operator ${{\mathcal {D}}}_{\mathrm{\scriptscriptstyle M}}=\hbar ^2\partial _w^2-2\cosh (w)$. This differential operator is related to the quantum curve considered above for the case $C=C_{0,2}$ by dropping the apparent singularities, setting $U=0$, and performing the change of coordinate $x=e^w$. To establish the relation between $D(U,\hbar )$ and a spectral determinant one can proceed along the lines of [22], the key observation being that zeros of the function $D(U,\hbar )$ are in one-to-one correspondence with eigenfunctions of the operator ${{\mathcal {D}}}_{\mathrm{\scriptscriptstyle M}}$ which decay for $w\rightarrow \pm \infty$, as follows easily from (3.33) using equation (3.45) which relates the diagonal elements of the matrix *E* in (3.33) to $D(U,\hbar )$.

One should note, however, that the spectral determinant studied in [55] is related to the partition functions studied in our paper in a somewhat intricate way. It should be interesting to clarify the relation between spectral determinants and topological string partition functions for this class of cases.

### Relation to Topological Recursion

The topological recursion [47] offers a rather flexible scheme for defining formal series expansions of various objects arising in mathematical physics including the large *N* expansion of matrix models, and the topological string partition functions. It is known that the topological recursion can in particular be used to describe the genus expansion of the topological string partition functions associated with toric Calabi–Yau manifolds [21, 48]. One may therefore expect that the expansion of the partition functions studied in this paper in powers of $\hbar$ can be described with the help of topological recursion.

An encouraging step in this direction has recently been made in [85]. This paper studies the tau-function of the Painlevé I equation, and shows how to describe the expansion in a parameter $\hbar$ which is a direct analog of our parameter $\hbar$ with the help of the topological recursion. Given that all Painlevé equations are obtained from Painlevé VI by certain collision limits, one expects to find a similar picture for all Painlevé equations. The results from [85] suggest that the objects most straightforwardly defined by topological recursion are the partition functions playing the role of coefficient functions in the expansions of generalised theta series type considered. In [85] it is shown for the case of Painlevé I that the object *defined* by these expansions satisfies the defining equation for the corresponding tau-function. It is furthermore shown in [85] how the Stokes data of Painlevé I are related to the variables appearing in the generalised theta series. It is clear that the generalisation to the cases studied in our paper would be very interesting.

A particularly interesting question is to what extent the non-perturbatively defined partition functions defined here are determined by the formal series expansions provided by the topological recursion. We believe that our work offers a few encouraging hints in this direction. We have observed a direct correspondence between certain distinguished sets of coordinates for the space of monodromy data and non-perturbatively defined partition functions. We have furthermore seen that the Borel summation of exact WKB allows us to define distinguished coordinates for the space of quantum curves in many cases. These coordinates can be seen as a generalisation of the objects frequently called quantum periods away from the locus of opers. This means that we have given a prescription how to assign non-perturbatively defined partition functions to (generalised) quantum periods. We have furthermore confirmed by explicit computations that the partition functions associated with the quantum periods give us exactly the topological string partition functions computed with the help of the topological vertex. We take this as a piece of evidence for the conjecture that the partition functions of our interest are determined by exact WKB and/or topological recursion in a way which is “as canonical as possible”.

A general framework relating topological recursion to structures from conformal field theory was recently proposed in [17] building upon previous work [18]. Although there are some suggestive similarities to the formalism used here, it is currently not clear to us how these approaches are related in detail.

### Relations to Hypermultiplet Moduli Spaces

The geometry of hypermultiplet moduli spaces in string compactifications has been studied intensively, see [8] for a review. The main challenges are to understand the corrections to the geometry associated with D-instantons and NS5-branes. There is a systematic procedure to compute D-instanton corrections from the spectrum of stable D-branes in the given string compactification [11]. Computing NS5-brane corrections is less well-understood, but it has been suggested in [10] that the twistor space partition function associated with a single NS5-brane admits a representation in the form of a generalised theta series with expansion coefficients given by the topological string partition functions (see equation (5.11) in [8]). The similarity between the generalised theta series appearing in [10] and the ones studied in our paper is probably not an accident. It has been proposed in [8] that the above-mentioned twistor space partition function may represent a section of a pre-quantum line bundle over a cluster variety. This suggestion has been developed further in [9]. The transition functions defining this line bundle are representable in terms of the generating functions for the cluster mutations describing transitions between different systems of Darboux coordinates which can be represented explicitly with the help of the Rogers dilogarithm function. A closely related line bundle has been studied in [101].

We find it intriguing to observe that the line bundle ${{\mathcal {L}}}_{\Theta }$ defined in this paper from the difference generating functions describing changes of Darboux-coordinates of FG–FG, FG–FN, FN–FG, and FN–FN types also admits a description using transition functions constructed from the Rogers dilogarithm. To make this concrete, let us recall that the basic building block for all of these changes of variables is the change of coordinates associated with the flip discussed in Sect. 4.3. It is possible to show (see Appendix H of the preprint version [35] of this paper) that a small change of normalisations yields an alternative representation for the transition function associated with the flip which can be recognised as an example of the transition functions considered in [9]. This suggests that the line bundles ${{\mathcal {L}}}_{\Theta }$ introduced in our paper are in fact related to the line bundles denoted ${{\mathcal {L}}}_{\Theta }$ in [9].

We believe that a detailed investigation of the relations between these two lines of research should be worthwhile. This might in particular suggest how to characterise the topological string partition functions for more general Calabi–Yau manifolds within a similar framework.

### Relation to a Variant of Quantum Teichmüller Theory

The difference generating functions associated with changes of coordinates are more than just analogs of the operators representing the changes of coordinates in quantum Teichmüller theory. The observations made in [80, Section 7.2] indicate that the theta series expansions (9.10) relate a representation of the quantised algebra ${{\mathcal {A}}}$ of functions on ${{\mathcal {M}}}_{{\textrm{ch}}}(C)$ in terms of finite difference operators acting on wave functions $Z_{w}({\textsf{x}})$ to an equivalent representation on sections ${{\mathcal {T}}}_{x_w}({\textsf{x}},\check{{\textsf{x}}})$ of the line bundle on ${{\mathbb {T}}}$ defined by the relations (9.3) in which all elements of ${{\mathcal {A}}}$ are represented as multiplication operators. The difference generating functions represent the operators associated with changes of coordinates in a variant of the quantum Teichmüller theory.

The relevant version of quantum Teichmüller theory is probably related to the theory developed in unpublished work of Fock and Goncharov [51], see [8, Section 5.3] for a brief discussion. Key ingredients of the relevant variant of quantum Teichmüller theory have been proposed in [9]. The relation to the usual quantum Teichmüller theory is subtle. Quantum Teichmüller theory has a parameter *b*. The case discussed here would formally correspond to the value $b=\textrm{i}$ for which the usual quantum Teichmüller theory is no longer defined.

We would finally like to express our expectation that our results can be recognised as a concrete realisation of fairly old ideas going back to [2, 115] relating topological string partition functions to the quantisation of the moduli spaces of complex structures in string theory. One may hope that generalisations of the emerging picture may lead to a unified understanding of various aspects of topological string theory.

## Free Fermions Twisted by Flat Bundles

This appendix outlines how CFT techniques allow one to represent the tau-functions as free fermion conformal blocks twisted by flat bundles.

### Flat Bundles

We are going to define conformal blocks for a system of free fermions on a Riemann surface *C* twisted by a flat bundle. To begin with, we shall consider a Riemann surface $C=C_{g,n}$ with genus *g* and *n* punctures. Recall that the structure of a flat bundle can be described by a sufficiently fine cover $\{{{\mathcal {U}}}_\alpha ;\alpha \in {{\mathcal {A}}}\}$ of *C* and a vector bundle *E* with a preferred system of trivialisations such that the transition functions $g_{\alpha \beta }:{{\mathcal {U}}}_\alpha \cap {{\mathcal {U}}}_{\beta }\rightarrow \textrm{GL}(N,{{\mathbb {C}}})$ are constant. Flat bundles differing by changes of local trivialisations represented by constant functions $h_\alpha :{{\mathcal {U}}}_{\alpha }\rightarrow \textrm{GL}_n({{\mathbb {C}}})$ are identified with each other. Flat bundles are in a canonical one-to-one correspondence with representations $\rho :\pi _1(C)\rightarrow \textrm{GL}(N,{{\mathbb {C}}})$. We will use this correspondence to label flat bundles by the corresponding representations $\rho$ of $\pi _1(C)$.

These data define a Riemann–Hilbert problem. The solution to the Riemann–Hilbert problem defined by a flat bundle on *C* is a pair $({{\mathcal {E}}},\nabla )$ of a holomorphic bundle ${{\mathcal {E}}}$ and a holomorphic connection $\nabla$. One may equivalently consider the row vector $\Psi =(\psi _1,\dots ,\psi _N)$ formed out of *N* flat sections of $\nabla$ forming a frame. In a local trivialization of ${{\mathcal {E}}}$ one may represent $\Psi$ by a matrix $\Psi (x)$ of holomorphic functions such that the analytic continuation $\Psi (\gamma .x)$ of $\Psi (x)$ along a closed curve $\gamma$ starting and ending at *x* satisfies $\Psi (\gamma .x)=\Psi (x)\rho (\gamma )$. The solution to this Riemann–Hilbert problem becomes unique after imposing additional conditions on the behaviour of $\Psi (x)$ at the *n* punctures.

We will mostly consider the case $N=2$ in this paper. In this case, one can always solve the Riemann–Hilbert problem by meromorphic opers, pairs $({{\mathcal {E}}}_{\textrm{op}},\nabla _{\textrm{op}})$, where ${{\mathcal {E}}}_{\textrm{op}}$ is the unique (up to isomorphism) extension

A.1 $$\begin{aligned} 0 \rightarrow K^{\frac{1}{2}}\rightarrow {{\mathcal {E}}}_{\textrm{op}}\rightarrow K^{-\frac{1}{2}}\rightarrow 0, \end{aligned}$$

and $\nabla _{\textrm{op}}$ can be represented with respect to the frame associated with the representation as

A.2 $$\begin{aligned} \nabla _{\textrm{op}}=\partial +\left( \begin{matrix} 0 & \quad -t \\ 1 & \quad 0\end{matrix}\right) dx. \end{aligned}$$

The matrix element *t* is allowed to have apparent singularities. Generically one needs a minimum of $3g-3+n$ apparent singularities in order to solve the Riemann–Hilbert problem defined on a Riemann surface of genus *g* and *n* punctures at which *t* has poles of second order. The matrix $\Psi$ formed out of the flat sections of $\nabla _{\textrm{op}}$ takes the form $$\Psi =\left( {\begin{smallmatrix} \eta _1' &  \eta _2' \\ \eta _1 &  \eta _2\end{smallmatrix}}\right) $$, where $\eta _1$, $\eta _2$ are two linearly independent solutions of $(\partial _x^2+t(x))\eta =0$.

### Free Fermions

The free fermion super VOA is generated by fields $\psi _s(z)$, $\bar{\psi }_s(z)$, $s=1,\dots ,N$, The fields $\psi _s(z)$ will be arranged into a row vector $\psi (z)=(\psi _1(z),\dots ,\psi _N(z))$, while ${\bar{\psi }}(z)$ will be our notation for the column vector with components $\bar{\psi }_s(z)$. The modes of $\psi (z)$ and ${\bar{\psi }}(z)$, introduced as

A.3 $$\begin{aligned} \psi (z)=\sum _{n\in {{\mathbb {Z}}}}\psi _{n} z^{-n-1}\,,\quad {\bar{\psi }}(z)=\sum _{n\in {{\mathbb {Z}}}} {\bar{\psi }}_{n} z^{-n}\,, \end{aligned}$$

are row and column vectors with components $\psi _{s,n}$ and $\bar{\psi }_{s,n}$, respectively, satisfying

A.4 $$\begin{aligned} \{\,\psi _{s,n}\,,\,{\bar{\psi }}_{t,m}\,\}=\delta _{s,t}\delta _{n,-m}\,,\qquad \{\,\psi _{s,n}\,,\,\psi _{t,m}\,\}=0\,,\qquad \{\,{\bar{\psi }}_{s,n}\,,\,{\bar{\psi }}_{t,m}\,\}=0. \end{aligned}$$

The Fock space ${{\mathcal {F}}}$ is a representation generated from a highest weight vector ${\mathfrak {f}}_{0}$ satisfying

A.5 $$\begin{aligned} \psi _{s,n}\cdot {\mathfrak {f}}_{0} =0\,,\quad n\ge 0\,,\qquad {\bar{\psi }}_{s,n}\cdot {\mathfrak {f}}_{0} =0\,,\quad n>0\,. \end{aligned}$$

${{\mathcal {F}}}$ is generated from ${\mathfrak {f}}_{0}$ by the action of the modes $\psi _{s,n}$, $n<0$, and ${\bar{\psi }}_{s,m}$, $m\le 0$.

We will also consider the conjugate representation ${{\mathcal {F}}}^*$, a *right* module generated from a highest weight vector ${\mathfrak {f}}_{0}^{*}$ satisfying

A.6 $$\begin{aligned} {\mathfrak {f}}_{0}^{*}\cdot \psi _{s,n}=0\,,\quad n< 0\,,\qquad {\mathfrak {f}}_{0}^{*}\cdot {\bar{\psi }}_{s,n}\,=0\,,\quad n\le 0\,. \end{aligned}$$

The Fock space ${{\mathcal {F}}}^*$ is generated from ${\mathfrak {f}}_{0}^{*}$ by the right action of the modes $\psi _{s,n}$, $n\ge 0$, and ${\bar{\psi }}_{s,m}$, $m> 0$. A natural bilinear form ${{\mathcal {F}}}^*\otimes {{\mathcal {F}}}\rightarrow {{\mathbb {C}}}$ is defined by the expectation value,

A.7 $$\begin{aligned} \langle \,{\mathfrak {f}}_0^*\cdot {\textsf{O}}_{{\mathfrak {f}}^*}\,,\,{\textsf{O}}_{{\mathfrak {f}}}\cdot {\mathfrak {f}}_0\,\rangle = \Omega ({\textsf{O}}_{{\mathfrak {f}}^*}{\textsf{O}}_{{\mathfrak {f}}}\cdot {\mathfrak {f}}_0), \end{aligned}$$

where $$\Omega ({\mathfrak {f}})=c$$ if $${\mathfrak {f}}= c\,{\mathfrak {f}}_{0} +\sum _{s=1}^N(\sum _{n<0} \psi _{s,n}{\mathfrak {f}}_{s,n} + \sum _{m\le 0}\bar{\psi }_{s,m}{\mathfrak {f}}_{s,m})$$.

### Free Fermion States from the Riemann–Hilbert Correspondence

We are now going to outline how to modify the formalism used in [36] to the higher genus situation, generalizing the approach of [3] appropriate for the case $N=1$.

A simple and natural way to characterise a state ${\mathfrak {f}}\equiv {\mathfrak {f}}_G\in {{\mathcal {F}}}$ is through the matrix $G(x,y)\equiv G_{\mathfrak {f}}(x,y)$ of two-point functions having matrix elements

A.8 $$\begin{aligned} G_{\mathfrak {f}}(x,y)_{st}=\big \langle \,\bar{\psi }_s(x)\psi _t(y) \,\big \rangle _{{\mathfrak {f}}} \equiv \frac{\langle \,{\mathfrak {f}}_{0}^{*}\,,\,\bar{\psi }_s(x)\psi _t(y)\,{\mathfrak {f}}\,\rangle }{\langle \, {\mathfrak {f}}_{0}^{*}\,,\,{\mathfrak {f}}\,\rangle }. \end{aligned}$$

Indeed, given a function *G*(*x*, *y*) such that

A.9 $$\begin{aligned} G(x,y)=\frac{{1}}{x-y}+A(x,y)\,, \qquad A(x,y)=\sum _{l\ge 0} y^{-l-1}\sum _{k>0}x^{-k}A_{kl}, \end{aligned}$$

there exists a state ${\mathfrak {f}}_G$ such that its two-point function $G_{{\mathfrak {f}}_G}$ is given by *G*(*x*, *y*). States ${\mathfrak {f}}_G$ having this property can be constructed as

A.10 $$\begin{aligned} {\mathfrak {f}}_G  =N_G \exp \bigg (\!-\sum _{k>0}\sum _{l\ge 0} \psi _{-k}\cdot A_{kl}\cdot \bar{\psi }_{-l}\bigg )\, {\mathfrak {f}}_{0} \,, \end{aligned}$$

with $N_G\in {{\mathbb {C}}}$ being a normalisation constant.

We are interested in two-point functions *G*(*x*, *y*) having a multivalued analytic continuation to the Riemann surfaces *C* with given monodromies with respect to both *x* and *y*. The monodromies describing the analytic continuation in *x* are required to act on *G*(*x*, *y*) from the left, while the analytic continuation in *y* generates monodromies acting from the right,

A.11 $$\begin{aligned} G(x,\gamma _r.y)=G(x,y)\cdot M_r,\qquad G(\gamma _r.x,y)=M_r^{-1}\cdot G(x,y). \end{aligned}$$

This characterizes *G*(*x*, *y*) as a solution to a variant of the Riemann–Hilbert problem formulated above where one allows a first-order pole at $y=x$.

In order to construct solutions to this problem, let us choose a reference connection $\nabla _0$ on the holomorphic bundle ${{\mathcal {E}}}$ furnished by the solution to the Riemann–Hilbert problem discussed in Sect. A.1. Choosing flat sections $\Psi$ and $\Psi _0$ of $\nabla$ and $\nabla _0$, respectively, one can form the combination ${\hat{\Psi }}(x)=(\Psi _0(x))^{-1}\Psi (x)$. *G*(*x*, *y*) can then be constructed in the following form

A.12 $$\begin{aligned} G_{\Psi }(x,y)=({\hat{\Psi }}(x))^{-1}S(x,y){\hat{\Psi }}(y), \end{aligned}$$

with *S*(*x*, *y*) being the Szegö-kernel [49].^39^ One may, in particular, choose ${{\mathcal {E}}}={{\mathcal {E}}}_{\textrm{op}}$ as above, relating the choice of $\nabla _0$ to the choice of a reference oper defining a projective structure on *C*.

The construction of the fermionic states ${\mathfrak {f}}_{G}$ described above therefore gives us a natural way to assign fermionic states ${\mathfrak {f}}_\Psi  \equiv {\mathfrak {f}}_{G_\Psi }$ to solutions $\Psi$ of the Riemann–Hilbert problem.

### Conformal Blocks

We may associate a free fermion conformal block to a Riemann surface *C* with a flat bundle as follows. Let *P* be a point on *C*, and ${{\mathcal {U}}}_P$ be an open neighbourhood of *P* which is part of the cover defining the given flat bundle. Let *x* be a local coordinate in ${{\mathcal {U}}}_P$ vanishing at *P*, and let $\Psi _P(x)$ be the function of a complex variable *x* representing the solution to the Riemann–Hilbert problem on ${{\mathcal {U}}}_P$. From these data one may then define states ${\mathfrak {f}}_\Psi  \in {{\mathcal {F}}}$ and ${\mathfrak {f}}_\Psi ^*\in {{\mathcal {F}}}^*$ characterised by infinite sets of linear equations of the form

A.13 $$\begin{aligned} \psi [g]\cdot {\mathfrak {f}}_\Psi  =0,\qquad \bar{\psi }[\bar{f}]\cdot {\mathfrak {f}}_\Psi  =0,\qquad {\mathfrak {f}}_\Psi ^*\cdot \psi [g]=0,\qquad {\mathfrak {f}}_\Psi ^*\cdot \bar{\psi }[\bar{f}]=0, \end{aligned}$$

where

A.14 $$\begin{aligned} \psi [{g}]\,=\,\frac{1}{2\pi \textrm{i}}\int _{{{\mathcal {C}}}} dx\; \psi (x)\cdot {g}(x)\,,\qquad \bar{\psi }[\bar{f}\,]\,=\,\frac{1}{2\pi \textrm{i}}\int _{{{\mathcal {C}}}} \textrm{d}x\; \bar{f}(x)\cdot \bar{\psi }(x)\,, \end{aligned}$$

with ${{\mathcal {C}}}\subset {{\mathcal {U}}}_P$ being a sufficiently small circle around *P*, and $g\in {{W}}_{P}(\Psi )$, $\bar{f}\in \bar{W}_{P}(\Psi )$, with

A.15 $$\begin{aligned} \begin{aligned}&g\in {{W}}_{P}(\Psi )=\Big \{\,{\hat{\Psi }}^{-1}(x)\cdot v(x);\;\,{v}(x)(dx)^{\frac{1}{2}}\in {{\mathbb {C}}}^N\otimes H^0\big (C\!\setminus \!\{P\},K^{\frac{1}{2}}\big ) \,\Big \}\,,\\&\bar{f}\in \bar{W}_{P}(\Psi )=\Big \{\,\bar{v}(x)^t\cdot {\hat{\Psi }}(x);\;\,\bar{v}(x)(dx)^{\frac{1}{2}} \in {{\mathbb {C}}}^N\otimes H^0\big (C\!\setminus \!\{P\},K^{\frac{1}{2}}\big )\,\Big \}\,. \end{aligned} \end{aligned}$$

The linear equations (A.13), in the following referred to as free fermion Ward identities, determine ${\mathfrak {f}}_\Psi$ and ${\mathfrak {f}}_\Psi ^*$ up to multiplicative constants. The normalisation is determined by fixing the corresponding partition functions $Z_C(\Psi )=\langle {\mathfrak {f}}_0^*,{\mathfrak {f}}_\Psi  \rangle$ and $Z_C^*(\Psi )=\langle {\mathfrak {f}}_\Psi ^*,{\mathfrak {f}}_0 \rangle$. Normalising ${\mathfrak {f}}_\Psi$ and ${\mathfrak {f}}_\Psi ^*$ such that $Z_C (\Psi )=Z_C^{*}(\Psi )$, one sees that ${\mathfrak {f}}_\Psi$ and ${\mathfrak {f}}_\Psi ^*$ are two different ways to package the same information.

There is a generalisation of this construction assigning vectors ${\mathfrak {f}}_{\Psi ,n}\in {{\mathcal {F}}}^{\otimes n}$ to *n*-tupels of points $P_1,\dots ,P_n$. However, in the same way as in the case $n=1$ considered before one finds that the vectors ${\mathfrak {f}}_{\Psi ,n}$ are uniquely characterised by the multipoint analogs of (A.13) together with the corresponding partition function $Z_C(\Psi )=\langle {\mathfrak {f}}_0^{\otimes n},{\mathfrak {f}}_{\Psi ,n} \rangle$, a function which can be chosen to be *n*-independent. This means that the information defining a conformal block, in our case represented by $\Psi$ and $Z_C(\Psi )$, can be “localised” at arbitrary *n*-tuples of points. This is a special case of a phenomenon often referred to as “propagation of vacua” in the CFT literature.

Infinitesimal reparameterisations of the coordinate *x* are generated by the free fermion energy–momentum tensor *T*(*x*) constructed in the standard way as a bilinear expression in the fermions. Conformal blocks of the free fermion algebra are automatically conformal blocks for the Virasoro algebra. This means that the identities

A.16 $$\begin{aligned} T[v]\,{\mathfrak {f}}_{\Psi }=0,\qquad T[v]=\int _{{{\mathcal {C}}}} \textrm{d}x \,v(x)T(x), \end{aligned}$$

hold for all vector fields $v=v(x)\partial _x$ on ${{\mathcal {U}}}_P\!\setminus \!\{P\}$ that extend holomorphically to the rest of *C*, and vanish at the punctures.

There is a natural connection on the line bundle of free fermion conformal blocks over the moduli space ${{\mathcal {P}}}_{g,n}$ of triples (*C*, *P*, *x*). In order to define it, let us note that the tangent space $T{{\mathcal {P}}}_{g,n}$ can be represented as the space of equivalence classes [*v*] of vector fields *v* on the annulus modulo vector fields that extend holomorphically to the rest of *C*. The connection on the line-bundle of conformal blocks over ${{\mathcal {P}}}_{g,n}$ is then defined by having flat sections ${\mathfrak {f}}_\Psi$ satisfying

A.17 $$\begin{aligned} \delta _{[v]} {\mathfrak {f}}_{\Psi }=T[v]\,{\mathfrak {f}}_{\Psi }, \end{aligned}$$

where *v* is a vector field on the annulus representing an element of ${{\mathcal {P}}}_{g,n}$. The partition functions $Z_C(\Psi )=\langle {\mathfrak {f}}_0^*, {\mathfrak {f}}_{\Psi } \rangle$ associated with ${\mathfrak {f}}_{\Psi }$ will then be a section of the corresponding line bundle over ${{\mathcal {M}}}_{g,n}$ which is flat with respect to the connection defined by

A.18 $$\begin{aligned} \delta _{[\![v]\!]} Z_{C}(\Psi )=t[v]\,Z_C(\Psi ), \qquad t[v]:=\frac{\langle {\mathfrak {f}}_0^*, T[v]{\mathfrak {f}}_{\Psi } \rangle }{\langle {\mathfrak {f}}_0^*, {\mathfrak {f}}_{\Psi } \rangle }, \end{aligned}$$

where $[\![v]\!]$ is an equivalence class in the double quotient defined by considering vector fields *v* on the annulus modulo vector fields that extend holomorphically to the rest of *C* on the one hand, and modulo vector fields that extend holomorphically to the disc $D_P$, on the other hand. This double quotient is according to the Virasoro uniformisation theorem isomorphic to the Teichmüller space $T{{\mathcal {M}}}_{g,n}$, see [50, Section 17] or [45, Section 2.4] for useful reviews.

An observation going back to [97] identifies *t*[*v*] as the Hamiltonian generating the isomonodromic deformation of the connection $\nabla =\partial +A(x)dx$ induced by a variation of the complex structure of *C* in the direction $[\![v]\!]$. One thereby identifies the flat section $Z_C(\Psi )$ of the connection defined by (A.18) with the isomonodromic tau-function.

The connection defined in (A.18) is not flat, but only projectively flat. A concise discussion of this issue can be found in [111, Section 1.8]. The curvature can be trivialised locally. The data one needs to pick in order to achieve this are equivalent to a family of reference opers varying holomorphically over a given subset of the moduli space ${{\mathcal {M}}}_{g,n}$ of complex structures of $C_{g,n}$.

### Flat Bundles with Regular Singularities

We will say that the solution $\Psi$ of the Riemann–Hilbert problem has regular singular behaviour at $P\in C$ if the preferred system of local trivialisations can be chosen such that the corresponding solution $\Psi$ of the Riemann–Hilbert problem is on ${{\mathcal {A}}}_P\equiv {{\mathcal {U}}}_P{\setminus }\{P\}$ represented by a function $\Psi _P(x)$ having the form $\Psi (x)=\Phi (x)x^{{{\textsf{d}}}}$, with $\Phi (x)$ regular at $x=0$, and ${{\textsf{d}}}=\textrm{diag}(\sigma _1,\dots ,\sigma _N)$. As explained in [36] one may represent the conformal block in terms of a vector ${\mathfrak {f}}_{\Psi ,\sigma }^*$ in a twisted representation ${{\mathcal {F}}}^*_\sigma$ of the free fermion algebra in which the fermions $\psi (x)$, $\bar{\psi }(x)$ have diagonal monodromy around $x=0$. The vector ${\mathfrak {f}}_{\Psi ,\sigma }^*$ is characterised by a system of equations very similar to (A.13). The case where $\Psi$ is regular at *P* is the special case ${{\textsf{d}}}=0$.

When $\Psi$ has two regular singular points $P_i$, $i=1,2$, corresponding coordinates $x_i$, and choices of frames on $D_i$ such that $\Psi (x_i)=\Phi (x_i)x^{{{\textsf{d}}}_i}$, one can also define an operator $Y_{\Psi }:{{\mathcal {F}}}_{\sigma _1}\rightarrow {{\mathcal {F}}}_{\sigma _{2}}$ satisfying

A.19 $$\begin{aligned} \psi _2[g]\, Y_{\Psi }=Y_{\Psi } \, \psi _1[g], \qquad \bar{\psi }_2[\bar{f}]\,Y_{\Psi }=Y_{\Psi } \, \bar{\psi }_1[\bar{f}]. \end{aligned}$$

where

A.20 $$\begin{aligned} \psi _i[{g}]\,=\,\frac{1}{2\pi \textrm{i}}\int _{{{\mathcal {C}}}_i} \textrm{d}x\; \psi (x)\cdot {g}(x)\,,\qquad \bar{\psi }_i[\bar{f}\,]\,=\,\frac{1}{2\pi \textrm{i}}\int _{{{\mathcal {C}}}_i} \textrm{d}x\; \bar{f}(x)\cdot \bar{\psi }(x)\,, \end{aligned}$$

for $i=1,2$, with ${{\mathcal {C}}}_i$ being a small circles around $P_i$, for $i=1,2$, respectively, and

A.21 $$\begin{aligned} \begin{aligned}&g\in {{W}}_{P_2P_1}(\Psi )=\Big \{\,{\hat{\Psi }}^{-1}(x)\cdot v(x);\;\,{v}(x)(dx)^{\frac{1}{2}}\in {{\mathbb {C}}}^N\otimes H^0\big (C\!\setminus \!\{P_2,P_1\},K^{\frac{1}{2}}\big ) \,\Big \}\,,\\&\bar{f}\in \bar{W}_{P_2P_1}(\Psi )=\Big \{\,\bar{v}(x)^t\cdot {\hat{\Psi }}(x);\;\,\bar{v}(x)(dx)^{\frac{1}{2}} \in {{\mathbb {C}}}^N\otimes H^0\big (C\!\setminus \!\{P_2,P_1\},K^{\frac{1}{2}}\big )\,\Big \}\,. \end{aligned} \end{aligned}$$

Acting with the operator $Y_{\Psi }$ on ${\mathfrak {f}}_{\sigma _1}$ defines a vector ${\mathfrak {f}}_{\sigma _2}\in {{\mathcal {F}}}_{\sigma _2}$. This vector satisfies the same system of linear equations as ${\mathfrak {f}}_{\Psi ,\sigma _2}$. As the solution to this system of equations is unique up to normalisation, we can normalise $Y_{\Psi }$ such that we have $Y_{\Psi } {\mathfrak {f}}_{\sigma _1} ={\mathfrak {f}}_{\Psi ,\sigma _2}$. The operator $Y_{\Psi }$ may in this sense be regarded as a repackaging of the information contained in ${\mathfrak {f}}_{\Psi ,\sigma _2}$. For $\sigma _1=\sigma _2=0$ one recovers ${\mathfrak {f}}_{\Psi }$ and gets another manifestation of the propagation of vacua phenomenon.

The natural generalisation of (A.18) represents variations of $(C,P_2,x_2,P_1,x_1)$ in the form

A.22 $$\begin{aligned} \delta _{[v]}Y_{\Psi }=T_2[v_2]\,Y_{\Psi }-Y_{\Psi }\,T_1[v_1], \end{aligned}$$

with $(v_2,v_1)$ being a pair of vector fields on ${{\mathcal {U}}}_{P_i}\!\setminus \!\{P_i\}$ representing the variation [*v*] of $(C,P_2,x_2,P_1,x_1)$ by a generalisation of the Virasoro uniformisation theorem.

One may note that the fermionic Ward identities defining the states ${\mathfrak {f}}_{\Psi }$, ${\mathfrak {f}}_{\Psi }^{*}$ do not depend on the type of singular behaviour imposed at the *n* punctures of $C_{g,n}$. Given that irregular singularities can be defined by suitable collision limits from collections of regular singularities [67] one may therefore use the same formalism in order to define conformal blocks for free fermion systems twisted by flat bundles having irregular singularities at some points.

### Gluing

There are well-known operations on the conformal blocks associated with the geometric operations of cutting a Riemann surface *C* along a simple closed curve $\gamma$, and gluing of bordered Riemann surfaces by identifying annuli around boundary components.

#### Gluing of Riemann Surfaces

Let $C_i$, $i=1,2$, be two Riemann surfaces with punctures $P_i$ having neighbourhoods with coordinates $x_i$ vanishing at $P_i$, for $i=1,2$, respectively. Out of these data one may define families of Riemann surface *C* by considering the annuli $A_i=\{Q_i\in D_i;r_i<|x_i(Q_i)|<R_i\}$, and identifying pairs of points $(Q_1,Q_2)$ satisfying $x_1(Q_1)x_2(Q_2)=q$ to get an annulus $A\subset C$.

One may note that a change of the parameter *q* is equivalent to a rescaling of either $x_1$ or $x_2$. It is a matter of convenience if one wants to use the parameter *q*, or prefers to set $q=1$ using one of the parameters $r_i$ or $R_i$, for $i=1,2$ instead. This is a simple example for a more general issue. Gluing two surfaces $C_i$ with choices of local coordinates around $P_i$ yields a surface *C* having a complex structure which depends only very little on the choices we had made. Most of the possible changes of the local coordinates around the points $P_i$ will not change the complex structure of the glued surface *C*. Some of them will, as could be parameterised either by the parameter *q*, or left implicit in the choices of the radii $r_i$, $R_i$, for $i=1,2$.

This construction is easily extended to surfaces with flat bundles. If $\rho _i:\pi _1(C_i)\rightarrow \textrm{GL}(N,{{\mathbb {C}}})$ are the representations of $\pi _1(C_i)$ characterising flat bundles on $C_i$ for $i=1,2$, respectively, we may construct a representation $\rho :\pi _1(C)\rightarrow \textrm{GL}(N,{{\mathbb {C}}})$ as follows. Let us pick a base point in the annulus *A*, and pick systems of generators for $\pi _1(C_i)$, $i=1,2$. The union of the sets of generators for $\pi _1(C_i)$ forms a set of generators for $\pi _1(C)$. Without essential loss of generality we may assume that the sets of generators for $\pi _1(C_i)$ contain curves $\gamma _i$ contained in $A_i$ which get identified to the non-contractible curve $\gamma$ around *A* by gluing. The given representations $\rho _i$ will allow us to define a representation $\rho :\pi _1(C)\rightarrow \textrm{GL}(N,{{\mathbb {C}}})$ whenever the elements $\rho _1(\gamma _1)$ and $\rho _2(\gamma _2)$ coincide, defining $\rho (\gamma )=\rho _1(\gamma _1)=\rho _2(\gamma _2)$.

The resulting representation will, of course, not only depend on the conjugacy class of representations $\rho _i$, but on the choice of the representations $\rho _i$ itself. Restricting attention to representations $\rho _i$ for which $\rho (\gamma )\equiv \rho _i(\gamma _i)$, is diagonal for $i=1,2$, we get a residual freedom in the choice of $\rho _i$ represented by conjugating $\rho _i(\delta )$ for all $\delta \in \pi _1(C_i)$ with the same diagonal matrix. The resulting ambiguity can be described equivalently in terms of the solutions $\Psi _i(x)$, $x\in A$, of the Riemann–Hilbert problems defined by the flat bundles on $C_i$, $i=1,2$, by multiplying $\Psi _i(x)$ from the right by diagonal matrices. This amounts to a change of normalisation of the elements $\psi _{ik}$, $k=1,\dots ,N$, of a basis for the space of solutions to $(\partial _x+A_i(x))\psi _i(x)=0$ associated with $\Psi _i(x)=(\psi _{i1}(x),\dots ,\psi _{iN}(x))$, for $i=1,2$, respectively. One may notice that the issue of picking preferred trivialisations of the flat bundle around the points $P_i$ is somewhat analogous to the issue of choosing local coordinates around these points.

#### Gluing of Conformal Blocks

The gluing operation has a simple counterpart on the level of the conformal blocks. Instead of describing it in full generality let us focus on the case of conformal blocks associated with two surfaces $C_1$ and $C_2$ each twisted by flat bundles $\rho _1$ and $\rho _2$. As explained above, we may describe conformal blocks on *C* in terms the one-point localisation picture in terms of vectors ${\mathfrak {f}}_\Psi \in {{\mathcal {F}}}$ attached to a point $P_{\infty }\in C$ and a local coordinate $x_{\infty }$ on the disc $D_{\infty }$ around $P_\infty$. Without essential loss of generality let us consider the case where $P_{\infty }\in C_2$. As explained above, we may represent the conformal block attached to $(C_2,\rho _2)$ in a two-point localisation in terms of an operator $Y_{\Psi _2}:{{\mathcal {F}}}_\sigma \rightarrow {{\mathcal {F}}}$ satisfying the free fermion Ward identities (A.19). The conformal block attached to $(C_1,\rho _1)$ can be represented by a vector ${\mathfrak {f}}_1\in {{\mathcal {F}}}_{\sigma }$. To the surface *C* defined in the gluing construction we may then associate the states $Y_{\Psi _2}\,{\mathfrak {f}}_1\in {{\mathcal {F}}}$. It is not hard to show that $Y_{\Psi _2}\,{\mathfrak {f}}_1$ satisfies the free fermion Ward identities defining conformal blocks on *C* with flat bundle constructed by the gluing construction described above.

The behaviour under variations of $(C,P_\infty ,x_\infty )$ can be found as follows. Let *v* be a vector field on $D_{\infty }$ representing a variation of $(C,P_\infty ,x_\infty )$. Our goal is to compute $\delta _{[v]}Y_{\Psi _2}{\mathfrak {f}}_1$. Having constructed *C* by the gluing construction allows us to represent any variation of $(C,P_\infty ,x_\infty )$ as the sum of a variation $\delta _{[v],2}$ of $(C_2,P_{\infty },x_{\infty },P_2,x_2)$ and a variation $\delta _{[v],1}$ of $(C_1,P_1,x_1)$, leading to $\delta _{[v]}Y_{\Psi _2}{\mathfrak {f}}_1 = (\delta _{[v],2}Y_{\Psi _2}){\mathfrak {f}}_1 +Y_{\Psi _2}(\delta _{[v],1}{\mathfrak {f}}_1 )$. There is a pair of vector fields $(v_\infty ,v_2)$ which represents the deformation $\delta _{[v],2}$ of $(C_2,P_{\infty },x_{\infty },P_2,x_2)$, and there is a vector field $v_1$ representing the deformation $\delta _{[v],1}$ of $(C_1,P_1,x_1)$. We may assume that $v_2=v_1\equiv v_A$. Indeed, it follows from the Virasoro uniformisation that the analytic continuation of the vector field *v*, restricted to *A* can be used to define both $(v_\infty ,v_2)$ and $v_1$. This allows us to compute

A.23 $$\begin{aligned} \delta _{[v]}Y_{\Psi _2}{\mathfrak {f}}_1 &= (\delta _{[v],2}Y_{\Psi _2}){\mathfrak {f}}_1 +Y_{\Psi _2}(\delta _{[v],1}{\mathfrak {f}}_1 ) \nonumber \\&=\big (T_\infty [v]Y_{\Psi _2}-Y_{\Psi _2}T_A[v_A]\big ){\mathfrak {f}}_1+Y_{\Psi _2} \big (T_A[v_A]{\mathfrak {f}}_1\big ) =T_\infty [v]Y_{\Psi _2}\,{\mathfrak {f}}_1. \end{aligned}$$

This shows that the vector $Y_{\Psi _2}{\mathfrak {f}}_1$ is flat with respect to the canonical connection on the line bundle of conformal blocks associated with $(C,P_\infty ,x_\infty )$.

### Cutting

For an oriented simple closed curve $\gamma$ on a Riemann surface *C*, let $A\equiv A_\gamma \subset C$ be an annulus in *C* containing $\gamma$ in its interior bounded by curves $\gamma _1$, $\gamma _2$ isotopic to $\gamma$. The orientation of $\gamma$ allows us to distinguish an “incoming” boundary, w.l.o.g. represented by $\gamma _1$, from the “outgoing” boundary $\gamma _2$. We may define $C_1'$ to be the connected component of the surface obtained by cutting along $\gamma _2$ which contains *A*. $C_2'$ will be defined in the same way with $\gamma _1$ taking the role of $\gamma _2$. A suitable choice of local coordinate *x* in *A* allows us to represent *A* w.l.o.g. as $A\simeq \{x\in {{\mathbb {C}}}; \rho _2>|x|>\rho _1\}$. From $C_i'$ one may define punctured Riemann surfaces $C_i$, $i=1,2$, by gluing punctured discs to the boundary components that have been created by the cutting operation.

It is easy to extend the cutting operation to surfaces *C* with flat bundles. To this aim, one may define flat bundles on $C_i$, for $i=1,2$, from the representations of $\pi _1(C_i)$ defined from the given representation $\rho :\pi _1(C)\rightarrow \textrm{GL}(N,{{\mathbb {C}}})$ by choosing a base-point in *A*, and restricting $\rho$ to generators of $\pi _1(C)$ represented by curves in $C_i'$, for $i=1,2$, respectively. We will restrict attention to flat bundles having diagonalisable monodromy along the non-contractible cycle in *A*, with eigenvalues being $e^{2\pi \textrm{i}\sigma _k}$, $k=1,\dots ,N$. The resulting flat bundles on $C_i$, $i=1,2$, will then admit solutions of the Riemann–Hilbert problem having a regular singularity at the punctures $P_i$ contained in $D_i$, characterised by the diagonal matrix ${\textsf{d}}=\textrm{diag}(\sigma _1,\dots ,\sigma _N)$, with $0\le \textrm{Re}(\sigma _k)<1$ for $k=1,\dots ,N$.

It is clear that cutting represents an inverse to the gluing operation. We may thereby represent the vector ${\mathfrak {f}}_\Psi$ as ${\mathfrak {f}}_\Psi =Y_{\Psi _2}{\mathfrak {f}}_{\Psi _1}$, leading to the following representation of the partition function

A.24 $$\begin{aligned} Z_C(\Psi )\,=\,\langle \,{\mathfrak {f}}_{\Psi _2}^{*}\,,\,{\mathfrak {f}}_{\Psi _1} \rangle \,. \end{aligned}$$

As explained in [36], one may represent the right hand side of (A.24) as a Fredholm determinant of the following form

A.25 $$\begin{aligned} Z_C(\Psi )\,=\,\langle \,{\mathfrak {f}}_{\Psi _2}^{*}\,,\,{\mathfrak {f}}_{\Psi _1} \rangle =\textrm{det}_{{{\mathcal {H}}}_+} (1+{\textsf{B}}_{\Psi _2}{\textsf{A}}_{\Psi _1}), \end{aligned}$$

where ${\textsf{A}}_{\Psi _1}$ and ${\textsf{B}}_{\Psi _2}$ are integral operators constructed from solutions $\Psi _1$ and $\Psi _2$ to the Riemann Hilbert problems defined by flat bundles on the surfaces $C_1$ and $C_2$ used in the gluing construction, respectively. It is in this way becoming clear that the compositions appearing in the gluing construction of free fermion conformal blocks are analytically well-defined. Whenever there is an explicit solution to the Riemann Hilbert problems on $C_1$ and $C_2$, one may use (A.25) to get combinatorial expansions of the isomonodromic tau-function including the expansions computed explicitly in [60, 61].

Recursive use of this construction yields Fredholm determinant representations for the free fermion conformal blocks associated with any pants decomposition. Conformal blocks associated with different pants decompositions are related by analytic functions of the complex structure moduli of *C* and of the flat bundle $\rho$ defining the conformal block.

## Footnotes

1 We started preparing a short note elaborating on this point.

2 On $C_{0,4}$ just adding one apparent singularity is sufficient, since the complex dimension of the oper locus on $C_{0,n}$ is $(n-3)$, and because each apparent singularity introduces one new complex modulus (more precisely, two moduli subject to one constraint).

3 In fact the two are related by a confluence limit, as will be illustrated in the following.

4 The relative inversion of the signs is related to the choice of conventions in Sect. 3.1.1.

5 This can be seen by observing that $\frac{1}{{\textsf{n}}^{(2)}{\textsf{n}}^{(1)}} = \frac{t_0^+(\sigma )}{t^+(\sigma )}$ by combining (3.7) and (3.4), and using the explicit expressions for $t_0^{+}, t^+$ given in (3.11)–(3.12) and for ${{\mathcal {N}}}^{(\eta )}$ given in (3.14a).

6 An equivalent way is to use the general theory of Stokes phenomena, which identifies the small section with that multiplying the Stokes factor featuring in the jump. By declaring a choice of small flat section near $z=0$, we implicitly fix a trivialization for the universal covering of the cylinder. Then, the small flat section corresponding to the clockwise image of $v_0$ will be $Y^{(0)}(z\, e^{-2\pi \textrm{i}})\cdot e_2 = Y^{(0)}(z)\cdot M_0^{-1} \cdot e_2$.

7 Here $s_1$ is associated with the bottom-left vertex, $s_2$ to the bottom-right vertex, $s_3$ to the upper-right vertex, and $s_4$ to the top-left vertex in Fig. 1. All four are evaluated at $z_*$ near the bottom-left vertex.

8 Here $s_1$ corresponds to the bottom-left vertex, $s_2$ to the top-right vertex, $s_3$ with to the top-left vertex, and $s_4$ is the image of $s_1$ after transporting clockwise around $z=0$.

9 It is understood that, just like for the case of the *s*-channel, there should be more than one choice of $\eta _u$. Each of these induces a different expansion of the tau function, but all will be related as discussed in Sect. 3.2, *mutatis mutandis*.

10 The relation between $(\sigma ,\eta )$ and $(\sigma ',\eta ')$ was given in Sect. 3.4.

11 To be fully precise, there exists a relative normalisation factor between the coordinates $\eta _{\ {\textrm{CPT}}}$ and $\eta '$ here, $e^{2\pi \textrm{i}\eta _{\ {\textrm{CPT}}}}=e^{2\pi \textrm{i}\eta '} \frac{{{\mathcal {E}}}(2(\sigma +1))}{{{\mathcal {E}}}(\sigma )}$, like in equation (3.19), with ${{\mathcal {E}}}(x)=(2\pi )^{-x} e^{\frac{\pi \textrm{i}}{2}x^2}\frac{G(1+x)}{G(1-x)}$. This corresponds to a change of normalisation of the corresponding normalised tau-functions ${{\mathcal {T}}}^{(\eta ')}$ and ${{\mathcal {T}}}^{(\eta _{\ {\textrm{CPT}}})}$ like in equation (3.20).

12 These graphs have been plotted using the Mathematica package [Npl].

13 The range of $\epsilon$ must be taken sufficiently small, for the pants decomposition to hold. This is because in some cases another non-degenerate ring domain may appear at finite $\epsilon \ne 0$, inducing a different pants decomposition.

14 There are precisely eight saddles, corresponding to BPS states of the $T_2$ trinion theory, the free theory of a half-hypermultiplet in the tri-fundamental representation of $SU(2)^{\times 3}$.

15 The relevant results are in the literature attributed to a paper in preparation by Koike and Schäfke. N. Nikolaev has announced an alternative proof in upcoming work [103].

16 For example, this is the case if $q_0$ is a Strebel differential (this is the case considered in [74] to construct Fenchel–Nielsen coordinates). However, one should keep in mind that the Strebel condition is sufficient but not necessary: having a maximal number of ring domains is a weaker condition.

17 More precisely, Borel resummation is being invoked separately within $C_i$, $i=1,2$. Then, by ‘Stokes lines traversing the annulus’ we mean the Stokes lines emanating from $b_i$ which flow into the puncture on $C_i$ at which the annulus is glued.

18 It may also happen that a single branch point occurs more than once along a boundary component of *A*. An example of this can be found in Fig. 4b), with *A* glued into the left-most puncture. As the bottom frame shows, the boundary of *A* features two branch points, each of them occurs twice as one travels along the boundary. This kind of degeneracy introduces a finite degree of additional freedom of choice.

19 Here and in (5.18), the appearance of opposite signs is related to the convention adopted in (3.3).

20 In the context of class ${{{\mathcal {S}}}}$ theories, these correspond to chambers of the Coulomb branch where the BPS spectrum includes a vectormultiplet. Due to wall-crossing, ring domains may be present only in certain chambers.

21 For example, if *C* is a sphere with only one irregular singularity there cannot be a ring domain because $\pi _1(C)$ is trivial.

22 There may be cases in which one has “accumulation of accumulation rays” (or higher iterations thereof, measured by the *Cantor-Bendixson rank* of the differential) where this does not always hold. Examples of this include the torus with one puncture, and compact surfaces.

23 Standard FN coordinates have an ambiguity by arbitrary integer shifts $V\rightarrow V U^k$ with $k\in {\mathbb {Z}}$, which is fixed in the definition of FN-type coordinates via the Exact WKB approach. One may ask whether this way of fixing *k* is compatible with the definition of (*U*, *V*) via (5.20) and the construction of (*X*, *Y*) corresponding to the WKB triangulation. In the following we argue that this is the case.

24 Active rays are rays from the origin to infinity in the plane ${\mathbb {C}}^\times$ (parametrised by $\hbar$), which separate wedges where the spectral network has a fixed topological type (see also Sect. 8).

25 The local form of *T* that we consider here is not fully generic, since in general *A* may be tessellated by more than two triangles. On the one hand, the present discussion can be suitably generalized to such cases. On the other hand, if one is merely interested in a topological ideal triangulation (as opposed to a WKB triangulation), it should be possible to reduce any case to the one we consider here, by a finite sequence of flips. The change of FG coordinates induced by these additional flips is known to be a rational transformation, which should be pre-composed with the change of coordinates we derive.

26 The function $a(u,\hbar )$ in [55] is almost (up to normalisation) equal to the function $\sigma (U,\hbar )$ obtained by restricting the coordinate function $\sigma$ on the space of quantum curves to the subspace represented by opers, assuming simple relations between *u* and *U*, and $\hbar$ and $\hbar$, respectively.

27 Yet another perspective on this is the observation that both FG- and FN-type coordinates are special types of ‘spectral coordinates’ [74]. Spectral coordinates are labelled by homology cycles on $\Sigma$. It was argued in [74] that fixing the cycle to correspond to a purely electric charge (as is the case for the BPS vectormultiplet), and varying $\hbar$ across a sequence of wedges leading up to the real positive axis, one finds that the corresponding coordinate is FG type away from the real axis, but eventually approaches the (exponentiated) FN coordinate $\sigma$.

28 Comparing with [15] one should note that [15] has fixed a prescription selecting one of the two possible cases.

29 This is an instance of the Dillinger–Delabaere–Pham formula, see, for example, [84] for details.

30 To avoid potential confusion, we stress that this *differs* from $$V^{(b_- b_+)} = V^{(0 b_+)} - V^{( 0b_-)} $$.

31 This corresponds to the minimal choice for the number of apparent singularities that is necessary to cover a patch of ${{\mathcal {M}}}_{\mathrm{{ch}}}$ around the oper submanifold parametrized by the quantum curve with $\hbar =0$.

32 One should note that the definition of $Q_1$ requires choosing preferred coordinates around $u_r$, for $r=1,\dots ,d$.

33 An important special case is given by the conformal limit of the non-abelian Hodge correspondence [37, 40]. It establishes bi-holomorphisms between the strata attached to different values of $\hbar$ of the stratification of ${{\mathcal {Z}}}$ obtained by gathering the triples having limits $\lim _{\hbar \rightarrow 0}[\xi \hbar ,{\mathcal {E}},\xi \nabla _\hbar ]$ in the same connected component [108].

34 With ${{\mathcal {T}}}(C)$ represented as a connected component of the character variety $\textrm{Hom}(\pi _1,\textrm{PSL}(2,{{\mathbb {R}}}))/\textrm{PSL}(2,{{\mathbb {R}}})$.

35 The inverse map will not be rational, in general. This means that the torus ${{\mathbb {T}}}_{\mathrm{\scriptscriptstyle FN}}$ containing the image of the FN-type coordinates is a finite cover of the torus ${{\mathbb {T}}}_{\mathrm{\scriptscriptstyle FG}}$ to which the FG-coordinates map.

36 The exists no trajectory connecting branch points. Closed trajectories are allowed.

37 In many cases there is a triangulation of *C* admitting a restriction to a ring domain isotopic to the triangulation of the twice punctured sphere defining the FG-type coordinates relevant for the Painlevé III case. The remaining cases can be covered with the trick used in [105].

38 This is not explicitly formulated in this way in [55], but this follows from the identification of the quantum periods $\Pi ^{\textrm{ex}}_{A,B}$ with FN-type coordinates mentioned in Sect. 10.3 together with the results of [55, Section 4.2].
